# Application of a 3D pseudocontinuous arterial spin-labeled perfusion MRI scan combined with a postlabeling delay value in the diagnosis of neonatal hypoxic-ischemic encephalopathy

**DOI:** 10.1371/journal.pone.0219284

**Published:** 2019-07-08

**Authors:** Shilong Tang, Xianfan Liu, Ling He, Bo Liu, Bin Qin, Chuan Feng

**Affiliations:** 1 Department of Radiology, Children's Hospital of Chongqing Medical University, Chongqing, China; 2 Ministry of Education Key Laboratory of Child Development and Disorders, Children's Hospital of Chongqing Medical University, Chongqing, China; 3 Key Laboratory of Pediatrics in Chongqing, Children's Hospital of Chongqing Medical University, Chongqing, China; 4 Chongqing International Science and Technology Cooperation Center for Child Development and Critical Disorders, Children's Hospital of Chongqing Medical University, Chongqing, China; McLean Hospital, UNITED STATES

## Abstract

**Background:**

Currently, there are many studies on the application of the 3D pseudocontinuous arterial spin-labeled (3D-pcASL) perfusion MRI technique for adult brain examinations, but few studies exist on the application of the technique for child brain examinations.

**Purpose:**

To explore the application of a 3D-pcASL perfusion MRI scan combined with postlabeling delay (PLD) for assessing neonatal hypoxic-ischemic encephalopathy (HIE).

**Materials and methods:**

Two-hundred neonates diagnosed with neonatal HIE were equally divided into five groups (40/group): 0- to <24-hour-old HIE group, 1- to <3-day-old HIE group, 3- to <7-day-old HIE group, 7- to <15-day-old HIE group and 15- to 28-day-old HIE group; 200 healthy neonates were equivalently divided. All 10 groups received a conventional and a 3D-pcASL perfusion MRI scan. For groups <3 days old, PLD values for the 3D-pcASL cerebral perfusion MRI scan were preset at 1025 ms; in all other groups, PLD values were preset at 1525 ms. CBF values for the 3D-pcASL cerebral perfusion MRI were compared between the HIE and corresponding control groups to determine the distinguishing characteristics of CBF values in HIE neonates.

**Results:**

On the 3D-pcASL cerebral perfusion MRI scan, in the 1- to <3-day-old groups, HIE neonate CBF values were higher than those of controls in all brain regions (excluding the frontal lobe); in the 0- to <24-hour-old and 3- to <7-day-old groups, HIE neonate CBF values were lower than those of corresponding controls in all brain regions; in the 7- to <15-day-old and 15- to 28-day-old groups, there were no significant differences in the CBF values between groups in any brain regions.

**Conclusions:**

The 3D-pcASL perfusion MRI scan combined with a PLD can assist in the early diagnosis of neonatal HIE, as this method more comprehensively reflects the HIE pathological process.

## Introduction

Neonatal hypoxic-ischemic encephalopathy (HIE) is a type of brain tissue injury. HIE is a common cause of childhood neurologic injury. HIE has the following clinical manifestations: patients have a good prognosis in mild cases and poor prognosis in severe cases; HIE can cause neonatal death in the short-term and result in some sequelae associated with neurologic injury in the long-term, such as delayed growth, mental retardation and epilepsy[1–4]. An early diagnosis of the severity and scope of a neonatal brain injury caused by HIE is the main difficulty and an important issue in current research because of the serious threat it poses to children's health.

In recent years, with the rapid development and clinical application of new MRI technologies, such as diffusion-weighted imaging, perfusion-weighted imaging, magnetic resonance spectroscopy, diffusion tensor imaging and magnetic susceptibility imaging, researchers can observe not only morphological changes but also the pathological process and molecular changes of HIE. In the abovementioned MRI technologies, neonatal cerebral blood flow can be accurately reflected using only a perfusion MRI scan. However, for detecting neonatal cerebral blood flow, the effect of the perfusion MRI technique is limited due to the use of a gadolinium contrast agent, lack of replicated testing (due to a cumulative effect), and other factors [5–8].

The 3D pseudocontinuous arterial spin-labeled (3D-pcASL) technique is a type of perfusion MRI technique that has several advantages, such as nonuse of a contrast agent, no radiation use, and repeatable testing. In this technique, a vascular perfusion MRI scan is performed with intra-arterial blood hydrone as an endogenous contrast agent. The mechanism of this technique is as follows: first, images are collected before and after the labeled blood hydrones reach the targeted imaging space; then, the collected images are subtracted; and finally, images with blood perfusion data are obtained [9–12]. Compared with the conventional perfusion imaging technique with an intravenous bolus injection, the 3D-pcASL perfusion MRI technique has the following advantages: a noninvasive examination, repeatable detection of the CBF values in the same sites of subjects without the use of contrast agents, good repeatability, and a fast MRI scan within only several minutes, among others [13–15].

The postlabeling delay (PLD) value refers to the time from the labeling of intra-arterial blood hydrones to the acquisition of image signals from the imaging space; the arterial transit time (ATT) refers to the time in which the intra-arterial blood hydrones reach the acquisition space from the labeling space [16–20]. As an important parameter for the 3D-pcASL perfusion MRI scan technique, the selection of the PLD value directly determines the accuracy of the CBF value. When the PLD values are less than the ATT values, the obtained CBF values are lower than the actual ones because the acquired magnetic resonance signals are low during the acquisition of image signals when the labeled intra-arterial blood hydrones do not reach the imaging space. When the PLD values are higher than the ATT values, the obtained CBF values are lower than the actual values because the acquired magnetic resonance signals are low during the acquisition of image signals when labeled intra-arterial blood shows over-relaxation. When the PLD values are equal to the ATT values, the obtained CBF values are equal to the actual values because the acquired magnetic resonance signals are high during the acquisition of image signals when the labeled intra-arterial blood just reaches the imaging space [21–23]. Therefore, in a 3D-pcASL cerebral perfusion MRI scan, the PLD values are by far the best only when the chosen PLD values are closest to the ATT values, and the obtained CBF values at this time are also closest to the true values. If the selected PLD values are too high or too low, the obtained CBF values will be lower than the true values.

Using the 3D-pcASL cerebral perfusion MRI technique, arterial blood flowing into the brain can be continuously labeled, 3D whole-brain fast imaging can be performed after labeling blood flowing into brain tissue, and finally, changes in the whole-brain blood flow can be detected. This technique has been widely used in clinical practice because of its advantages, such as being noninvasive, having repeatable functional imaging and being able to evaluate the whole brain without contrast agents [24–26]. Currently, there are many studies on the application of the 3D-pcASL perfusion MRI technique for adult brain examinations, but few studies exist on the application of the technique for child brain examinations, and very few studies exist on the application of the technique for neonatal brain examinations. However, in the abovementioned studies associated with the 3D-pcASL perfusion MRI technique, PLD values were usually chosen according to empirical values, with almost no consideration of the effect of the PLD values on the cerebral blood flow perfusion of neonates at different days of age. That is, the fact that there can be differences in the intra-arterial blood flow rate of neonates at different days of age is often not considered. In fact, improper PLD values can result in greater deviations between the detected neonatal CBF values and the actual ones, meaning the severity and scope of an HIE brain injury cannot be accurately determined.

The purposes of this study were to analyze and determine the CBF perfusion characteristics of different day-old healthy and HIE neonates and their differences under the optimal PLD values by determining the optimal PLD values that were appropriate for a 3D-pcASL cerebral perfusion MRI scan of different day-old neonates.

## Materials and methods

### Ethics statement

The study protocol was approved by the Human Ethics Committee of the Children's Hospital of Chongqing Medical University. Written informed consent was obtained from the parents or guardians of all patients before the examinations.

### Patients

#### Clinical pretest

In this study, 200 healthy full-term neonates (gestational age: 37–41 weeks) from our hospital from May 2016 to April 2017 were selected and divided into the following five control groups (40 per group): 0- to <24-hour-old control group, 1- to <3-day-old control group, 3- to <7-day-old control group, 7- to <15-day-old control group, and 15- to 28-day-old control group. In the five control groups, the ratios of boys to girls were 15:25, 21:19, 27:13, 17:23 and 23:17, respectively; the average days of age were 14.3±3.6 hours, 1.7±0.5 days, 5.1±1.2 days, 11.3±3.2 days and 22.6±3.6 days, respectively; the average gestational ages were 39.1±0.96 weeks, 39.5±1.12 weeks, 39.2±0.76 weeks, 39.2±1.05 weeks, and 39.3±0.84 weeks, respectively; and the average weights were 3.23±0.32 kilograms, 3.05±0.37 kilograms, 3.18±0.41 kilograms, 3.34±0.47 kilograms, and 3.89±0.51 kilograms, respectively. All neonates in this clinical pretest were suspected of having craniocerebral lesions and underwent magnetic resonance examination, but the results of these examinations revealed them to be normal neonates.

#### Clinical test

In this study, 200 HIE full-term neonates (gestational age: 37–41 weeks) from our hospital from May 2017 to March 2018 were selected and divided into the following five HIE groups (40 per group): 0- to <24-hour-old HIE group, 1- to <3-day-old HIE group, 3- to <7-day-old HIE group, 7- to <15-day-old HIE group and 15- to 28-day-old HIE group. In the five HIE groups, the ratios of boys to girls were 22:18, 24:16, 19:21, 18:22 and 19:21, respectively; the average days of age were 13.6±4.1 hours, 1.8±0.6 days, 5.3±1.1 days, 10.6±4.1 days, and 20.3±4.2 days, respectively; the average gestational ages were 39.3±1.07 weeks, 39±1.05 weeks, 39.7±0.89 weeks, 39.2±1.12 weeks, and 39.3±0.97 weeks, respectively; and the average weights were 3.21±0.28 kilograms, 3.07±0.37 kilograms, 3.16±0.45 kilograms, 3.40±0.39 kilograms, and 3.78±0.59 kilograms, respectively. The inclusion criteria for full-term neonates with HIE were as follows: a history of abnormal obstetrical diseases that could clearly lead to fetal intrauterine distress and serious manifestations of fetal intrauterine distress or a history of obvious asphyxia during childbirth; severe asphyxia at birth; neurological symptoms (e.g., euphoria, drowsiness, weakened Moro reflex) that occur soon after childbirth and last longer than 24 hours; and children who are clinically diagnosed with moderate HIE. The exclusion criteria were as follows: convulsions caused by intracranial bleeding, electrolyte disturbances and birth trauma, and brain injuries caused by genetic metabolic diseases, intrauterine infection and other congenital diseases.

### Imaging data collection and postprocessing

All neonates were given 10% chloral hydrate 0.5 ml/kg orally 20 minutes before the scan. Those who could not take the drug orally were given an anal injection at the same dose. After they were sedated and asleep, MRI was performed.

#### Clinical pretest

The clinical pretest was performed using the 3.0T magnetic resonance(discovery MR750; GE Medical Systems, Milwaukee, WI, USA) scanner and 8-channel head-neck combined coils. The plain MRI scans and 3D-pcASL scan of the brain were performed after the neonates had fallen asleep; the plain MRI scans included a transaxial T1FLAIR scan, T2FLAIR scan and T2WI scan; each of the 3D-pcASL sequences were acquired three times; the PLD values were chosen as 1025 ms, 1525 ms and 2025 ms. The 3D-pcASL perfusion MRI parameters were as follows: the TR values were 4387 ms (PLD 1025 ms), 4630 ms (PLD 1525 ms) and 4842 ms (PLD 2025 ms), respectively; the scanning times were 4 minutes and 15 seconds (PLD 1025 ms), 4 minutes and 29 seconds (PLD 1525 ms), and 4 minutes and 41 seconds (PLD 2025 ms), respectively; the FOV value was 21 cm × 21 cm; the TE value was 10.5 ms; the NEX value was five times; the slice thickness was 4.2 mm; and the number of acquisition slices was 30.

#### Clinical test

The clinical test was performed using a GE discovery MR750 3.0T magnetic resonance scanner and 8-channel head-neck combined coils. The 3D-pcASL sequences were collected only once. The PLD values were preset at 1025 ms for the 0- to <24-hour-old groups (HIE/control) and 1- to <3-day-old groups (HIE/control) and 1525 ms for all other groups (HIE/control). The other scanning parameters in the clinical test were the same as those of the clinical pretest.

**Fig 1:**
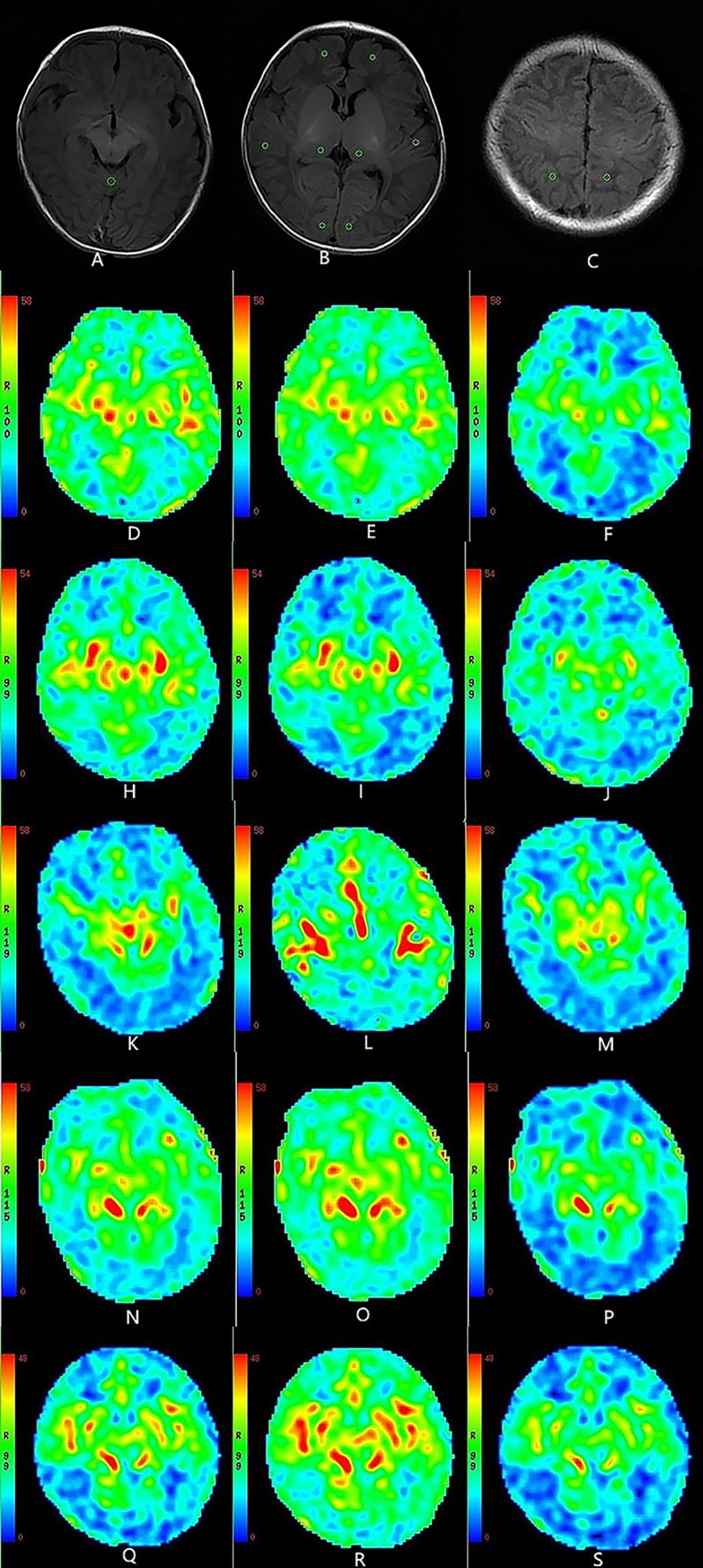
A-C. Schematic diagram of ROIs in the cerebellum, thalamus, occipital lobe, temporal lobe, frontal lobe and parietal lobe D-F. CBF map, healthy neonate, male, 11 hours old. Fig D. PLD value = 1025 ms; Fig E. PLD value = 1525 ms; Fig F. PLD value = 2025 ms. Red and green areas representing hyperperfusion in Fig D were greater than those in Fig E and F. H-J. CBF map, healthy neonate, female, 2 days old. Fig H. PLD value = 1025 ms, Fig I.PLD value = 1525 ms, Fig J. PLD value = 2025 ms. Red and green areas representing hyperperfusion in Fig H. were greater than those in Fig I and J. K-M. CBF map, healthy neonate, male, 5 days old. Fig K. PLD value = 1025 ms, Fig L.PLD value = 1525 ms, Fig M. PLD value = 2025 ms. Red and green areas representing hyperperfusion in Fig L were greater than those in Fig K and M. N-P. CBF map, healthy neonate, female, 12 days old. Fig N. PLD value = 1025 ms, Fig O.PLD value = 1525 ms, Fig P. PLD value = 2025 ms. Red and green areas representing hyperperfusion in Fig O were greater than those in Fig N and P. Q-S. CBF map, healthy neonate, male, 21 days old. Fig Q. PLD value = 1025 ms, Fig R. PLD value = 1525 ms, Fig S. PLD value = 2025 ms. Red and green areas representing hyperperfusion in Fig R were greater than those in Fig Q and S.

Data analysis: (1) Raw data were imported into the Functool software through the GE ADW 4.6 workstation; then, the CBF maps were obtained. (2) The CBF values in the regions of brain, such as the cerebellum, thalamus, occipital lobe, temporal lobe, parietal lobe and frontal lobe, were detected. (3) The round regions of interest (ROIs) with sizes of (55±2) mm^2^ were manually delineated, and the sizes, locations and levels of the ROIs were kept as consistent as possible in all cases; we tried to select the gray matter region during the selection of ROIs because in neonates with HIE, gray matter is more prone to ischemia due to hypoxia. We chose the ROIs to avoid the large blood vessel areas of the brain as much as possible[27–29] (Fig 1A–1C).

### Statistical analysis

Statistical analyses were performed using SPSS 22.0 statistical software, and the measurement data are expressed as the means ± standard deviations ($\bar{x}\pm s$).

In the 3D-pcASL cerebral perfusion MRI scan, the CBF values, which were obtained in the same brain regions of healthy neonates at the same days of age under different PLD values, were analyzed using ANOVA (meaning replicated testing was performed), and a pairwise comparison was performed using the Bonferroni method. The CBF values, which were obtained in the same brain regions of healthy and HIE neonates at the same days of age under the same PLD values, were analyzed using independent-sample t-test. The ages in days, gestational ages and body weights of HIE children and normal controls in the same age group were tested by the independent samples t-test. Differences were considered statistically significant at P<0.05.

## Results

### Clinical pretest

**Fig 2:**
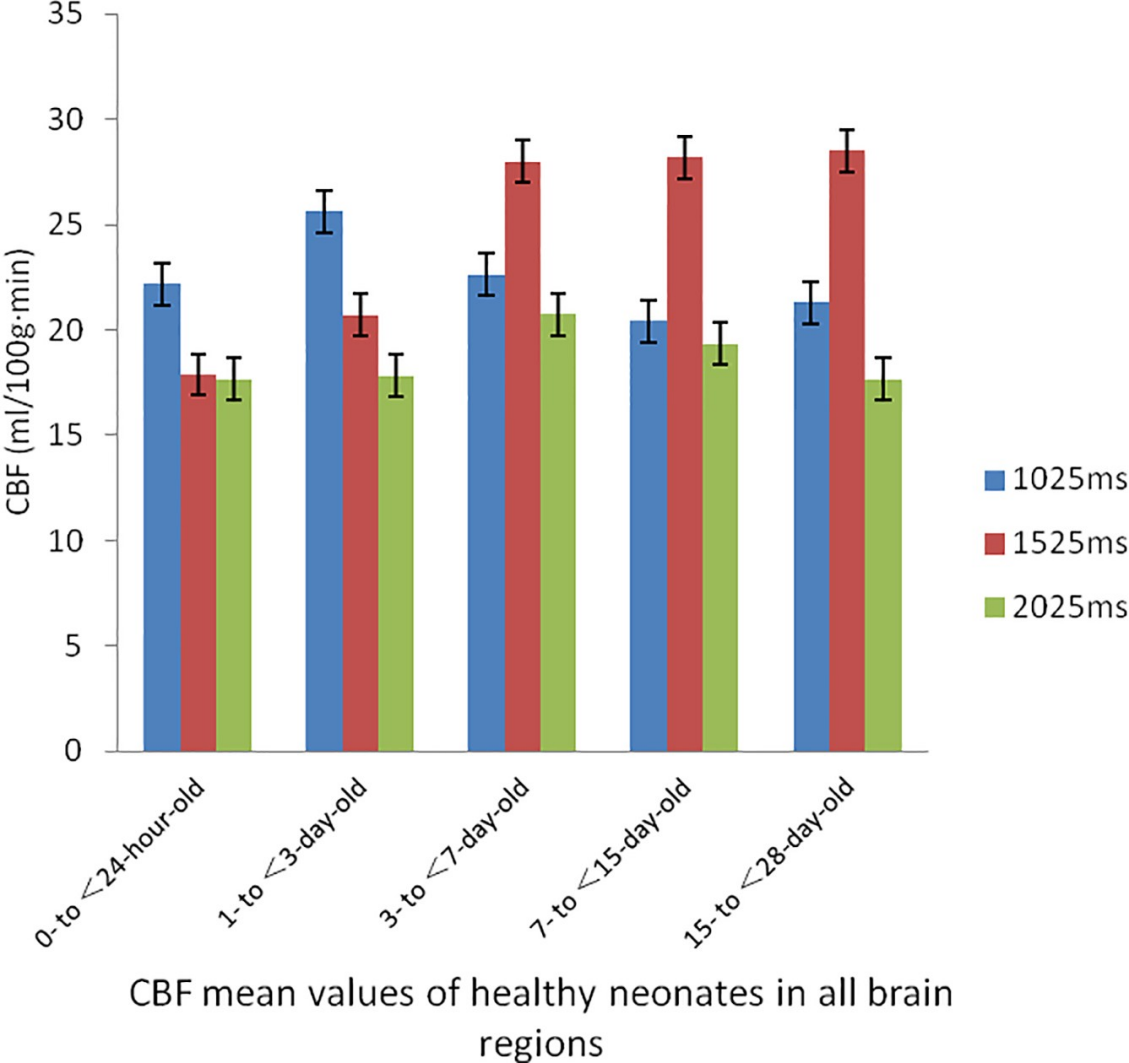
CBF mean values of neonates in all brain regions map. At the optimal PDL vaule, 0- to <24-hour-old groups and 3- to <7-day-old groups, the CBF values of the HIE neonates were lower than those of the healthy neonates in all brain regions;1- to <3-day-old groups, the CBF values of the HIE neonates were higher than those of the healthy neonates in all brain regions; 7- to <28-day-old groups, there were no significant differences in CBF values between the group of HIE neonates and healthy neonates.

The analysis results of the CBF values in the brain regions of healthy neonates at different days of age were as follows: in the 0- to <24-hour-old and 1- to <3-day-old control groups, the CBF values when the PLD value was 1025 ms were higher than those when the PLD values were 1525 ms and 2025 ms, and there were statistically significant differences between the CBF values under the different PLD values in the same brain regions of neonates at the same days of age (P<0.05). In the neonatal brain regions of all other control groups, the CBF values when the PLD value was 1525 ms were higher than those when the PLD values were 1025 ms and 2025 ms, and there were statistically significant differences between the CBF values under the different PLD values in the same brain regions of neonates at the same days of age (P<0.05) (Table 1, Fig 1D–1S, Fig 2).

**Table 1:** CBF mean values of healthy neonates in all brain regions.

group	CBF (ml/100 g·min)			F value	P value
	1025 ms	1525 ms	2025 ms		
0- to <24-hour-old	22.19±3.57	17.88±2.89	17.67±3.13	16.257	<0.001
1- to <3-day-old	25.63±3.63	20.69±4.55	17.81±3.75	9.136	<0.001
3- to <7-day-old	22.61±1.73	27.97±2.63	20.74±2.57	14.036	<0.001
7- to <15-day-old	20.43±3.52	28.19±2.39	19.33±2.87	15.127	<0.001
15- to <28-day-old	21.32±2.87	28.49±3.36	17.67±3.19	8.356	<0.001

**Note:** CBF = cerebral blood flow

### Clinical test

**Fig 3:**
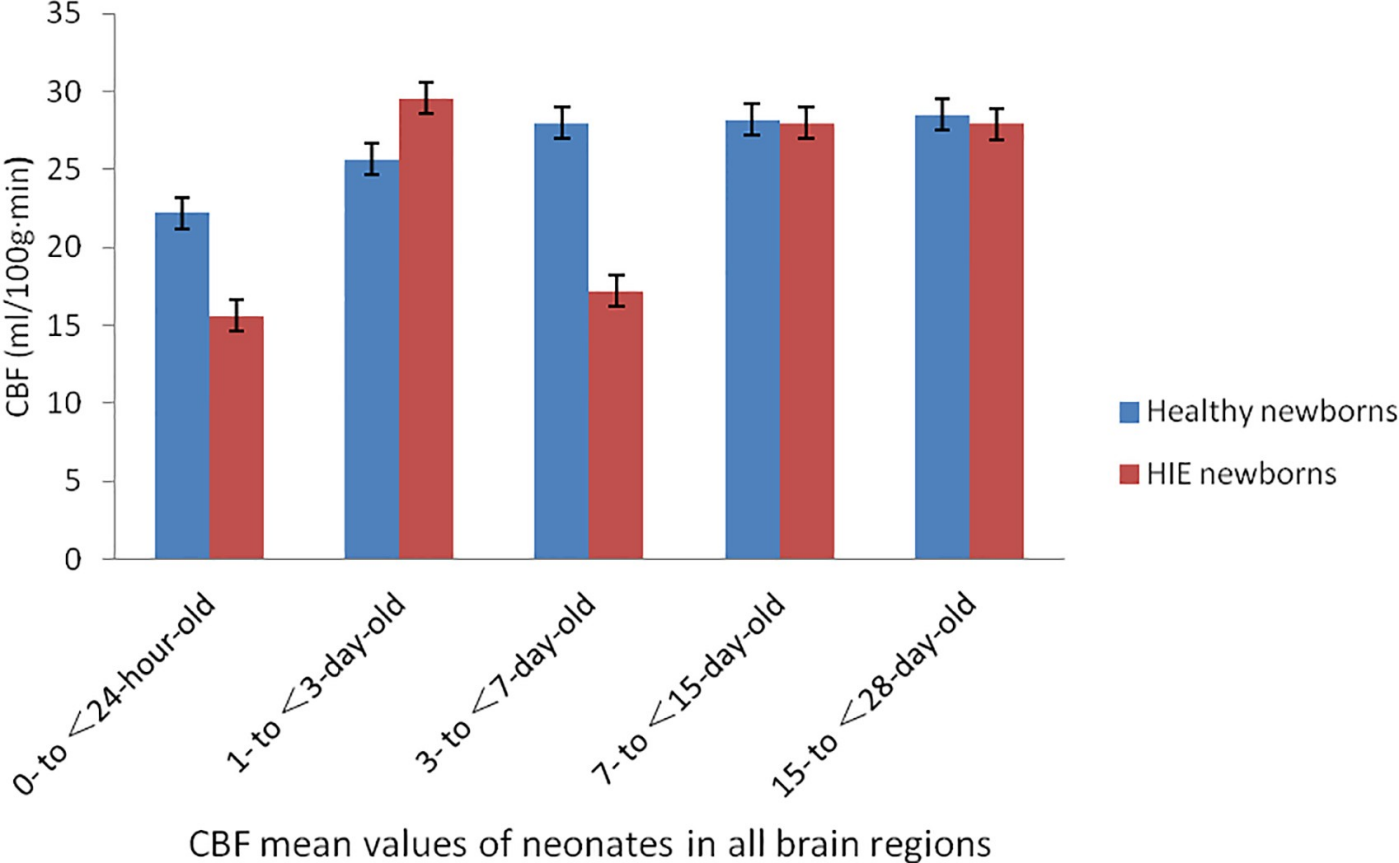
CBF mean values of neonates in all brain regions map. At the optimal PDL vaule, 0- to <24-hour-old groups and 3- to <7-day-old groups, the CBF values of the HIE neonates were lower than those of the healthy neonates in all brain regions;1- to <3-day-old groups, the CBF values of the HIE neonates were higher than those of the healthy neonates in all brain regions; 7- to <28-day-old groups, there were no significant differences in CBF values between the group of HIE neonates and healthy neonates.

**Fig 4:**
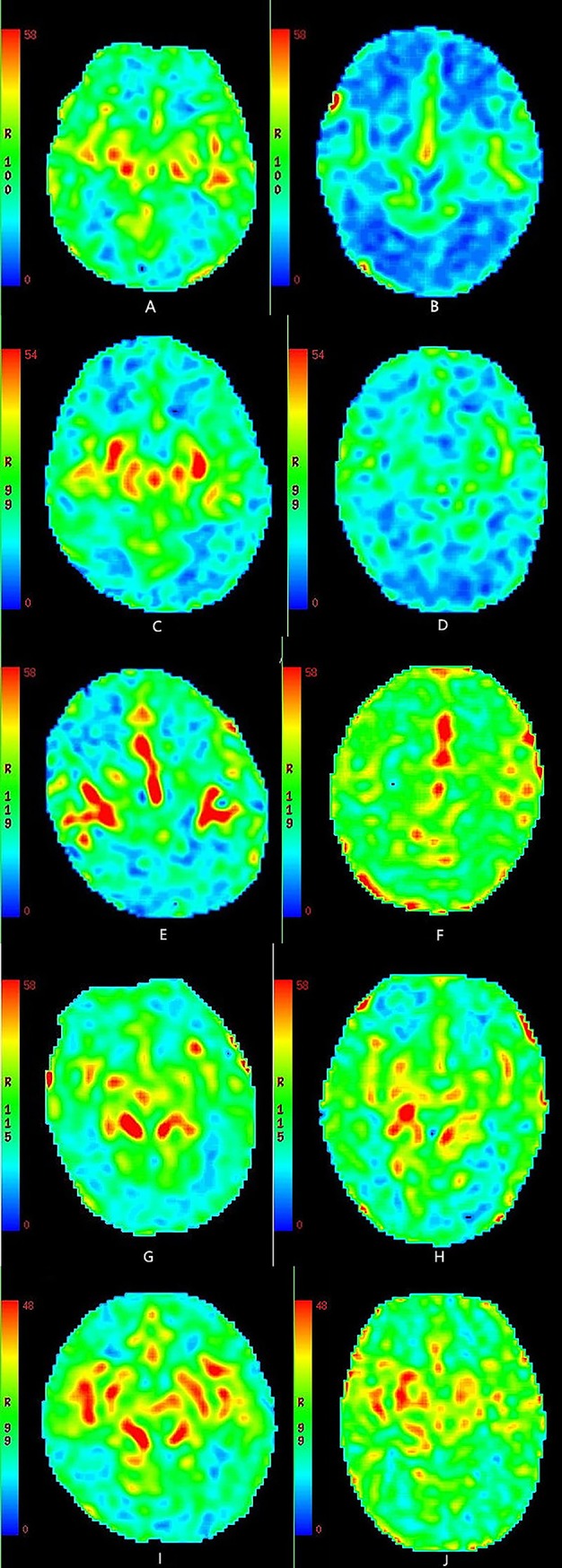
A-B CBF maps. Fig A. healthy neonate, male, 11 hours old, PLD value = 1025 ms; Fig B. HIE neonate, female, 14 hours old, PLD value = 1025 ms. Red and green areas representing hyperperfusion in Fig A were greater than those in Fig B. C-D. CBF maps. Fig C. healthy neonate, female, 2 days old, PLD value = 1025 ms; Fig D. HIE neonate, male, 2 days old, PLD value = 1025 ms. Red and green areas representing hyperperfusion in Fig C were greater than those in Fig D. E-F. CBF maps. Fig E. healthy neonate, male, 5 days old, PLD value = 1525 ms; Fig F. HIE neonate, female, 6 days old, PLD value = 1525 ms. Red and green areas representing hyperperfusion in Fig F were greater than those in Fig E. G-H. CBF maps. Fig G. healthy neonate, female, 12 days old, PLD value = 1525 ms; Fig H. HIE neonate, male, 13 days old, PLD value = 1525 ms. Red and green areas representing hyperperfusion in Fig G were almost identical to those in Fig H. I-J. CBF maps. Fig I. healthy neonate, male, 21 days old, PLD value = 1525 ms; Fig J. HIE neonate, female, 19 days old, PLD value = 1525 ms. Red and green areas representing hyperperfusion in Fig I were almost identical to those in Fig J.

In the 3D-pcASL cerebral perfusion MRI scan, when the conditions, such as brain regions, days of age and PLD values, were the same, the analysis results of the CBF values were as follows: in the 1- to <3-day-old groups (HIE/control), the CBF values of HIE neonates (not including the white matter of the frontal lobe) were higher than those of healthy neonates in the same brain regions, and the differences were statistically significant (P<0.05); in the 0- to <24-hour-old and 3- to <7-day-old groups (HIE/control), the CBF values of HIE neonates were lower than those of healthy neonates in the same brain regions, and the differences were statistically significant (P<0.05); in the 7- to <15-day-old and 15- to 28-day-old groups (HIE/control), there were no statistically significant differences in the CBF values between HIE and healthy neonates in the same brain regions (P>0.05). The ages in days, gestational ages and body weights of HIE children and normal controls in the same age group were tested by the independent samples t-test, and the difference was not statistically significant (P>0.05) (Tables 2–7, Fig 3, Fig 4A–4J).

**Table 2:** CBF values of neonates in all brain regions in the 0- to <24-hour-old group.

Encephalic region	CBF (ml/100 g·min)		T value	P value
	Healthy newborns	HIE newborns		
Cerebellum	23.60±4.12	18.25±2.78	7.487	<0.001
Thalamus	44.85±3.99	33.25±2.96	14.368	<0.001
Occipital lobe	11.97±1.89	8.03±1.27	12.731	<0.001
Temporal lobe	26.75±3.15	17.56±2.92	40.545	<0.001
Parietal lobe	13.29±2.11	8.29±2.01	11.287	<0.001
Frontal lobe	12.70±2.15	8.13±1.29	9.639	<0.001

**Note:** CBF = cerebral blood flow; HIE = hypoxic-ischemic encephalopathy

**Table 3:** CBF values of neonates in all brain regions in the 1- to <3-day-old group.

Encephalic region	CBF (ml/100 g·min)		T value	P value
	Healthy newborns	HIE newborns		
Cerebellum	25.14±3.18	30.56±4.13	12.263	<0.001
Thalamus	48.71±4.02	60.89±5.64	9.187	<0.001
Occipital lobe	13.19±1.02	19.57±3.16	21.238	<0.001
Temporal lobe	29.56±2.89	37.18±4.11	14.534	<0.001
Parietal lobe	15.67±1.73	20.57±3.76	8.271	<0.001
Frontal lobe	13.89±2.25	8.37±1.22	32.524	<0.001

**Note:** CBF = cerebral blood flow; HIE = hypoxic-ischemic encephalopathy

**Table 4:** CBF values of neonates in all brain regions in the 3- to <7-day-old group.

Encephalic region	CBF (ml/100 g·min)		T value	P value
	Healthy newborns	HIE newborns		
Cerebellum	27.61±3.96	18.23±2.37	6.936	<0.001
Thalamus	49.53±4.05	36.25±4.89	14.239	<0.001
Occipital lobe	13.18±1.72	8.23±1.63	19.368	<0.001
Temporal lobe	28.66±2.84	20.27±2.57	20.254	<0.001
Parietal lobe	15.83±3.04	9.92±1.66	17.335	<0.001
Frontal lobe	14.01±1.99	10.23±1.89	9.479	<0.001

**Note:** CBF = cerebral blood flow; HIE = hypoxic-ischemic encephalopathy

**Table 5:** CBF values of neonates in all brain regions in the 7- to <15-day-old group.

Encephalic region	CBF (ml/100 g·min)		T value	P value
	Healthy newborns	HIE newborns		
Cerebellum	28.54±2.71	29.11±3.27	0.532	1.136
Thalamus	50.75±4.16	48.97±4.21	0.791	0.673
Occipital lobe	15.33±1.64	15.21±2.36	1.369	0.493
Temporal lobe	30.12±3.15	29.22±3.23	0.256	1.235
Parietal lobe	17.56±2.89	18.01±2.15	0.795	1.027
Frontal lobe	15.13±1.74	15.22±1.29	1.569	0.784

**Note:** CBF = cerebral blood flow; HIE = hypoxic-ischemic encephalopathy

**Table 6:** CBF values of neonates in all brain regions in the 15- to 28-day-old group.

Encephalic region	CBF (ml/100 g·min)		T value	P value
	Healthy newborns	HIE newborns		
Cerebellum	28.13±2.15	27.89±3.12	0.573	0.148
Thalamus	52.38±4.51	50.87±5.13	1.636	0.927
Occipital lobe	15.21±1.67	15.66±2.39	0.459	1.143
Temporal lobe	33.76±3.03	32.18±2.97	1.782	0.925
Parietal lobe	17.33±2.36	18.11±2.12	0.267	1.039
Frontal lobe	16.28±1.39	16.59±1.37	1.783	0.672

**Note:** CBF = cerebral blood flow; HIE = hypoxic-ischemic encephalopathy

**Table 7:** CBF mean values of neonates in all brain regions.

G**roup**	CBF (ml/100 g·min)		F value	P value
	Healthy newborns	HIE newborns		
0- to <24-hour-old	22.19±3.57	15.59±2.14	13.124	<0.001
1- to <3-day-old	25.63±3.63	29.53±4.02	15.248	<0.001
3- to <7-day-old	27.97±2.63	17.19±2.12	8.192	<0.001
7- to <15-day-old	28.19±2.39	27.96±3.37	11.385	0.897
15- to <28-day-old	28.49±3.36	27.89±3.59	13.251	0.763

**Note:** CBF = cerebral blood flow

## Discussion

The cranial intra-arterial blood flow rate can change with the days of age of neonates. To minimize the influences caused by age differences, the neonates in this study were divided into five groups according to their days of age: 0- to <24-hour-old group, 1- to <3-day-old group, 3- to <7-day-old group, 7- to <15-day-old group and 15- to 28-day-old group. According to previous studies[28–30], when the 3D-pcASL sequence is applied to the adult brain, the optimal PLD value is between 1525 and 2025 ms. Because the blood flow rate in neonate brains is faster than that in adult brains, and only three PLD values of 3D-pcASL sequence on our MRI equipment were less than and equal to 2025 ms, including 1025 ms, 1525 ms and 2025 ms, only three types of PLD values were preset in the 3D-pcASL cerebral perfusion MRI scan in the clinical pretest: 1025 ms, 1525 ms and 2025 ms; the other PLD values were not added to the clinical pretest to determine the optimal PLD values of neonates at different days of age.

In the clinical pretest, the analysis results of the CBF values in all brain regions of neonates at different days of age were as follows: in the 0- to <24-hour-old and 1- to <3-day-old control groups, the PLD value of 1025 ms was closest to the cerebral ATT values, and the CBF values at this time were highest in all brain regions; the PLD values of 1525 ms and 2025 ms were more than the cerebral ATT values; and the CBF values at this time were lower in all brain regions. In the other control groups, a PLD value of 1525 ms was closest to the cerebral ATT values, and the CBF values at this time were highest in all brain regions; the PLD value of 1025 ms was lower and the PLD value of 2025 ms was higher than the cerebral ATT values, so the CBF values were lower under the two PLD values. Therefore, in the 0- to <24-hour-old and 1- to <3-day-old control groups, the optimal PLD value was 1025 ms; in all other control groups, the optimal PLD value was 1525 ms.

According to the findings from the clinical tests, in the 0- to <24-hour-old groups (HIE/control), the CBF values of the HIE neonates were lower than those of the healthy neonates in all brain regions, possibly because cerebral perfusion cannot be increased by vasodilatation because of the anoxic decrease of the cerebrovascular regulation function; the CBF values then decreased in all brain regions. In the 1- to <3-day-old groups (HIE/control), the CBF values of the HIE neonates were higher than those of the healthy neonates in all brain regions (not including the frontal lobe region), possibly because the compensatory increase of the cerebral blood flow caused by acute anoxic brain injury resulted in increases in the CBF values in all brain regions. In addition, the CBF values of HIE neonates were lower than those of healthy neonates in the frontal lobe region, possibly because the preferential redistribution of the cerebral blood flow to other regions of the brain made the ischemia of the white matter of the frontal lobe more severe. In the 3- to <7-day-old groups (HIE/control), the CBF values of HIE neonates were lower those of the healthy neonates in all brain regions. The probable pathomechanism of the above results is as follows: the cerebral arteries could not increase cerebral perfusion by vasodilatation after severe cerebral hypoxia, and an increased cerebral perfusion pressure resulted in the decompensation of the cerebrovascular self-regulation function. In the 7- to <15-day-old group (HIE/control) and 15- to 28-day-old group (HIE/control), there were no significant differences in CBF values between the group of HIE neonates and healthy neonates. This may be because the brain tissue hypoxia and cerebral blood perfusion of HIE neonates in this age group was improved and restored after treatment, respectively, thereby leading to no significant differences in CBF values between HIE neonates and healthy neonates[31–36].

Compared with previous studies published by other scholars on the application of ASL in neonatal HIE, this study shows the following advantages. First, the newborns were divided into 5 groups according to age in days, and the optimal PLD value for each age group was found to achieve a more accurate CBF value. Second, each age group had a large sample size of up to 40 cases to obtain more representative results.

This study also has the following limitations. First, only neonates who were clinically diagnosed with moderate HIE were included in this clinical trial, while those with mild and severe HIE were not included. Second, because the CBF value obtained by the ASL technology was a dynamic value of blood flow, any transient difference in blood flow can have a certain effect on the results. Third, another limitation of this study is that all neonates used sedatives during the examination. Tranquilizers are known to affect the state of intracranial blood circulation. However, the dose of sedatives used in this study was lower, in the range of ordinary sedatives. Therefore, the dose had less influence on intracranial blood circulation. However, because of the noise of magnetic resonance examination and the lengthy scanning time, the success rate of MRI scanning for neonates that did not cooperate with the examination in a natural sleeping state was low. Therefore, for neonates who did not cooperate with the examination, it was necessary to perform the MRI scanning in a sedative state. The above shortcomings need to be further improved in future research.

In summary, cerebral blood flow perfusion can be reflected by the 3D-pcASL technique, but the combination of the 3D-pcASL technique and a plain MRI scan technique can help in early diagnosis because the pathological process of HIE neonates is reflected more comprehensively.

## Supporting information

S1 File The patient CBF map.zip are within the supporting information files. (ZIP)
