# Supplementary figures and images for: Application of a 3D pseudocontinuous arterial spin-labeled perfusion MRI scan combined with a postlabeling delay value in the diagnosis of neonatal hypoxic-ischemic encephalopathy
Source: PLoS One. 2019 Jul 8;14(7):e0219284. doi: 10.1371/journal.pone.0219284 (PMC6613698; doi:10.1371/journal.pone.0219284)

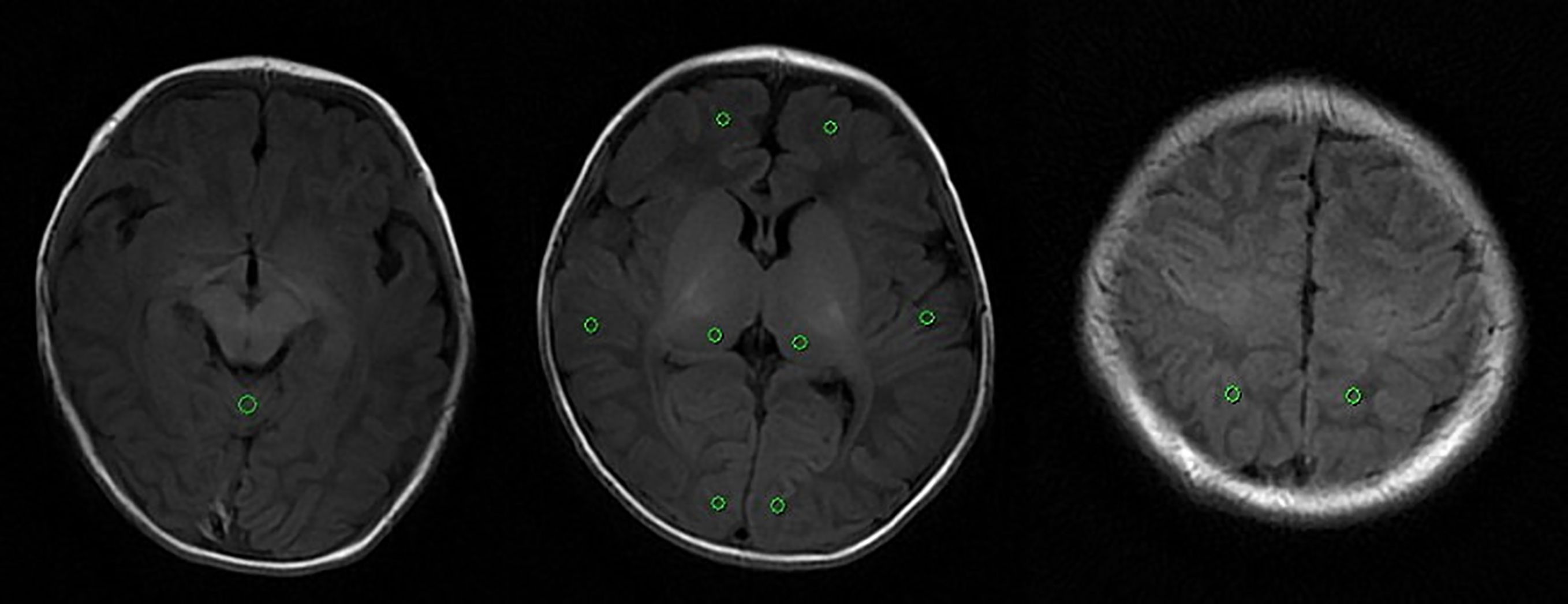

Supplement: S1 File — (ZIP) [file pone.0219284.s001.zip › patient CBF map/Figure1.tif]

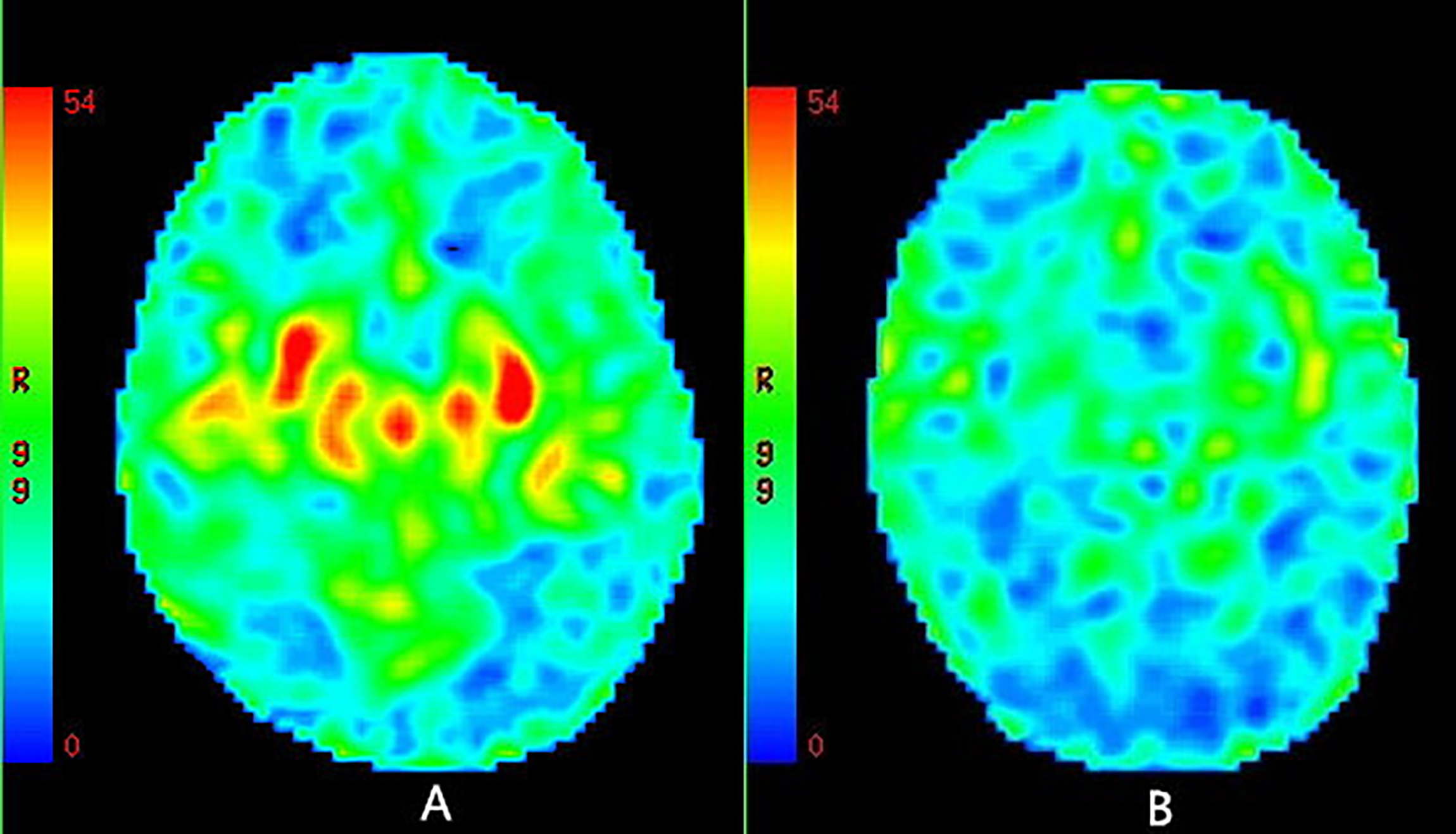

Supplement: S1 File — (ZIP) [file pone.0219284.s001.zip › patient CBF map/Figure10.tif]

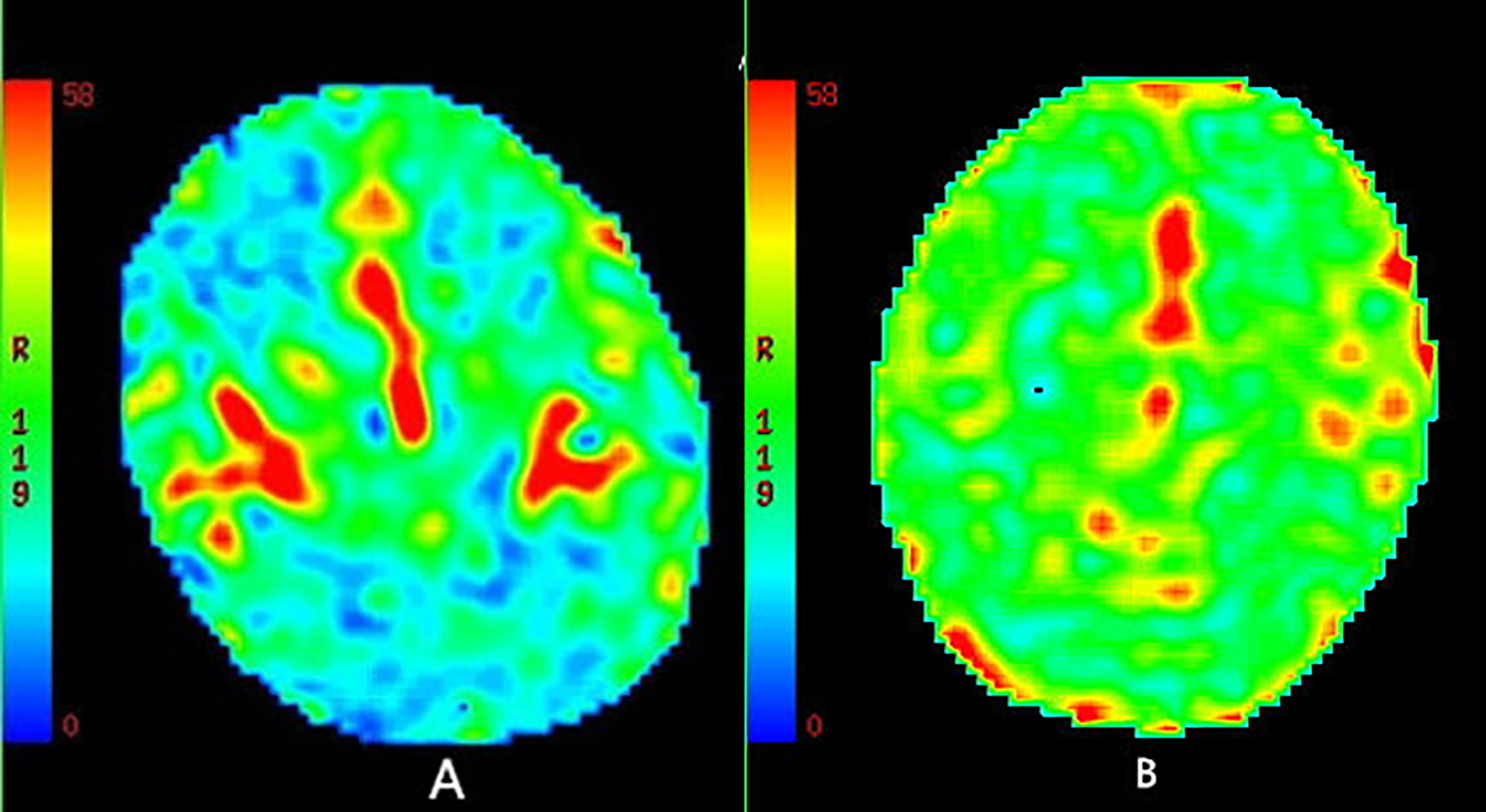

Supplement: S1 File — (ZIP) [file pone.0219284.s001.zip › patient CBF map/Figure11.tif]

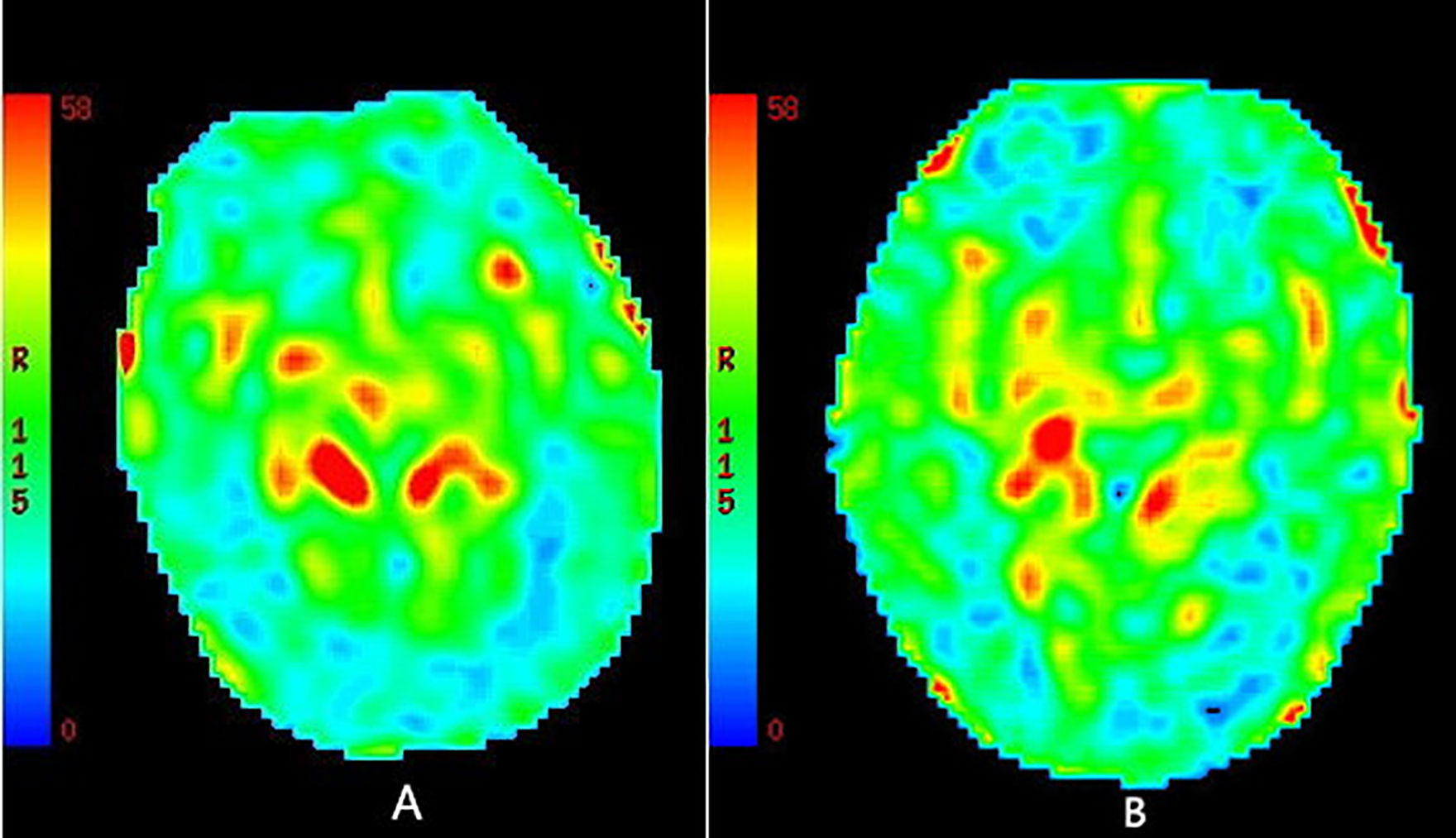

Supplement: S1 File — (ZIP) [file pone.0219284.s001.zip › patient CBF map/Figure12.tif]

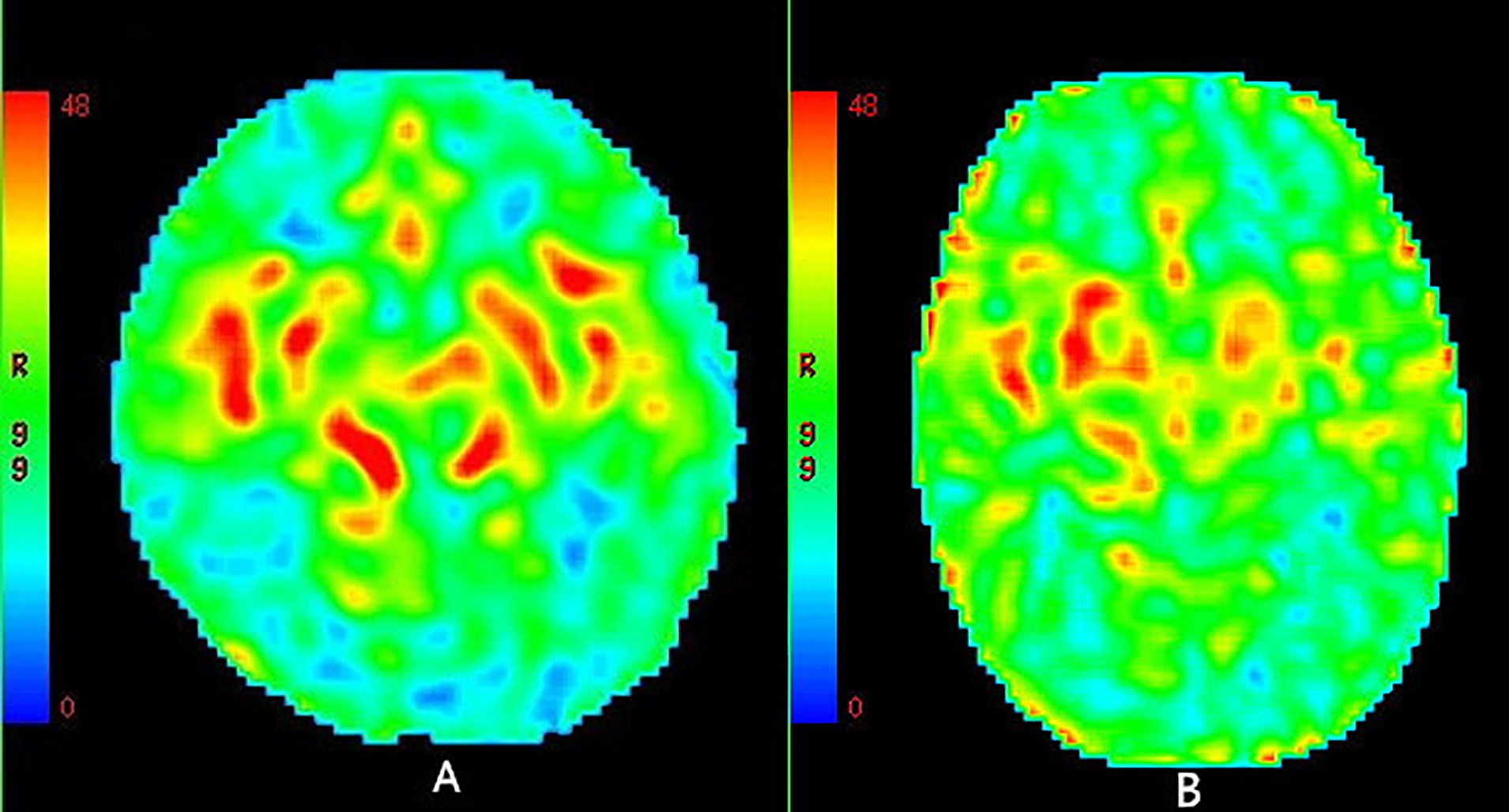

Supplement: S1 File — (ZIP) [file pone.0219284.s001.zip › patient CBF map/Figure13.tif]

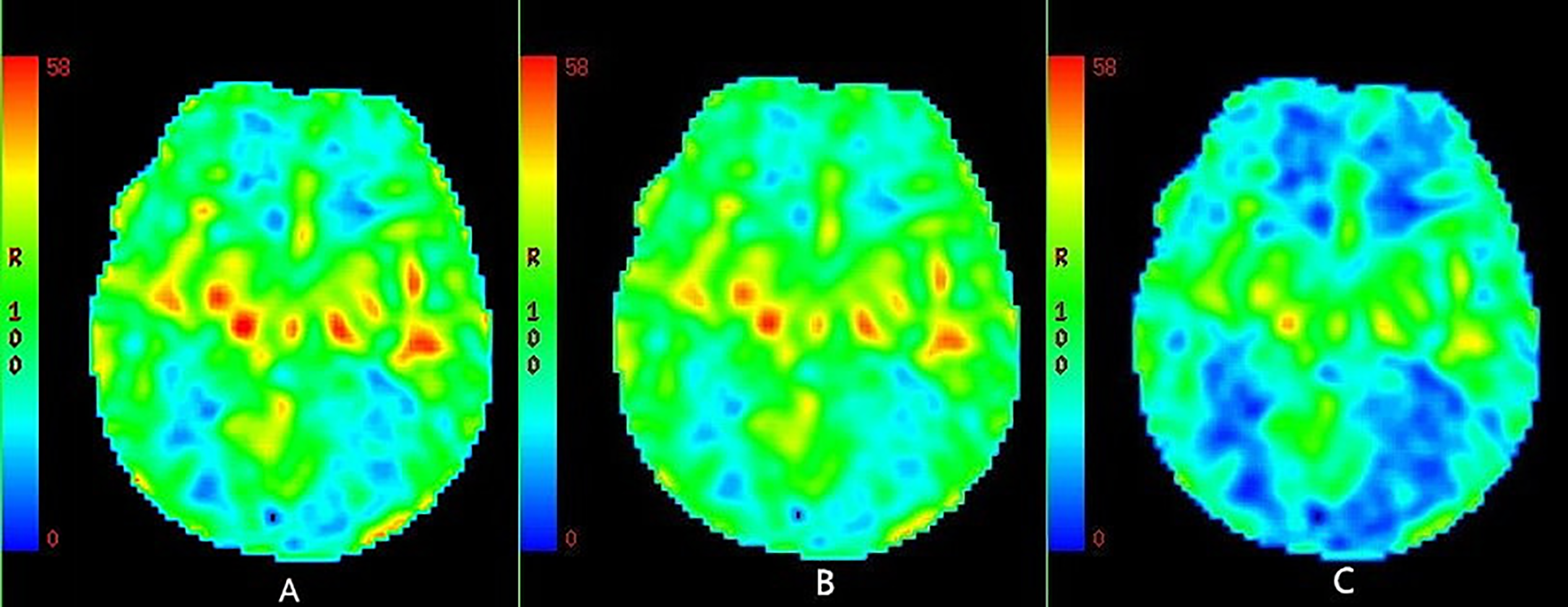

Supplement: S1 File — (ZIP) [file pone.0219284.s001.zip › patient CBF map/Figure3.tif]

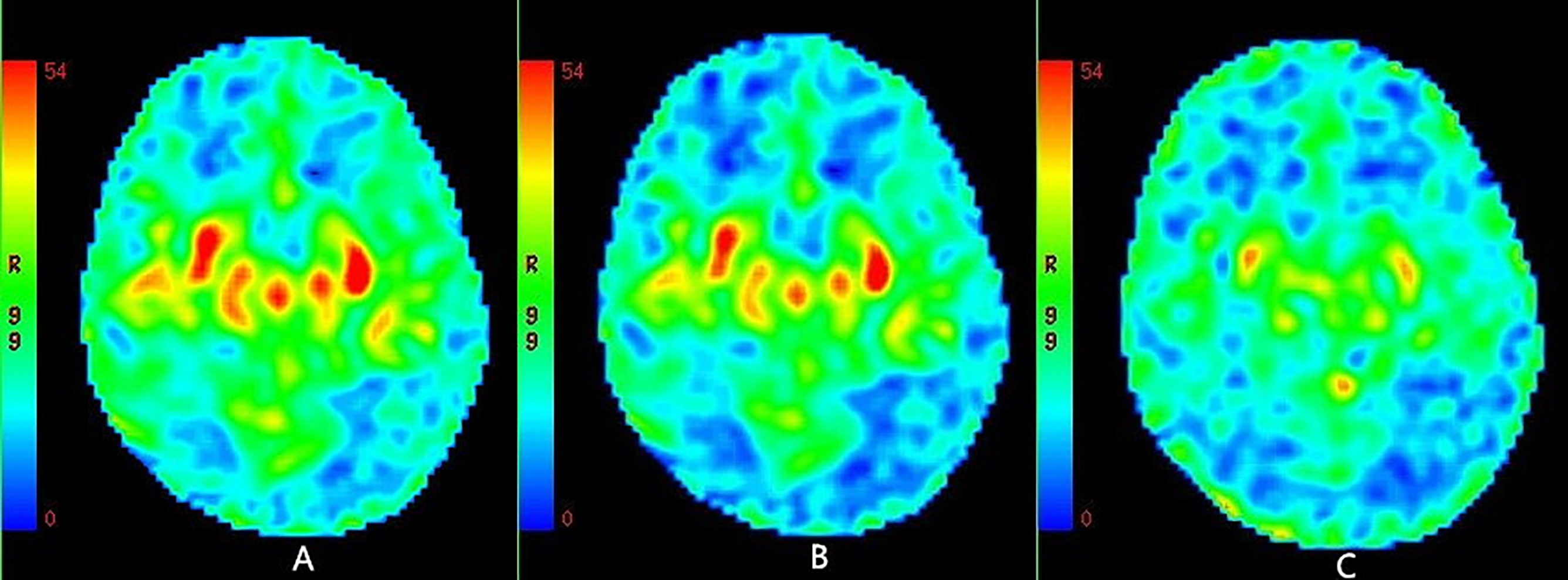

Supplement: S1 File — (ZIP) [file pone.0219284.s001.zip › patient CBF map/Figure4.tif]

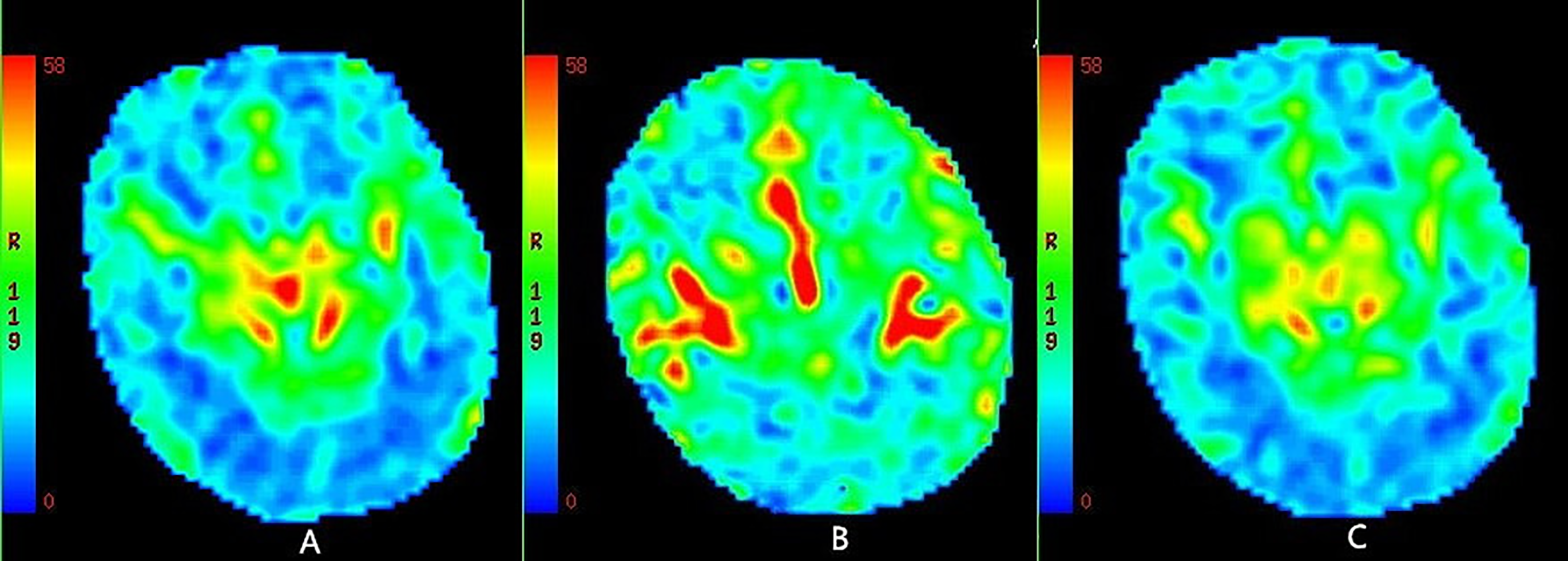

Supplement: S1 File — (ZIP) [file pone.0219284.s001.zip › patient CBF map/Figure5..tif]

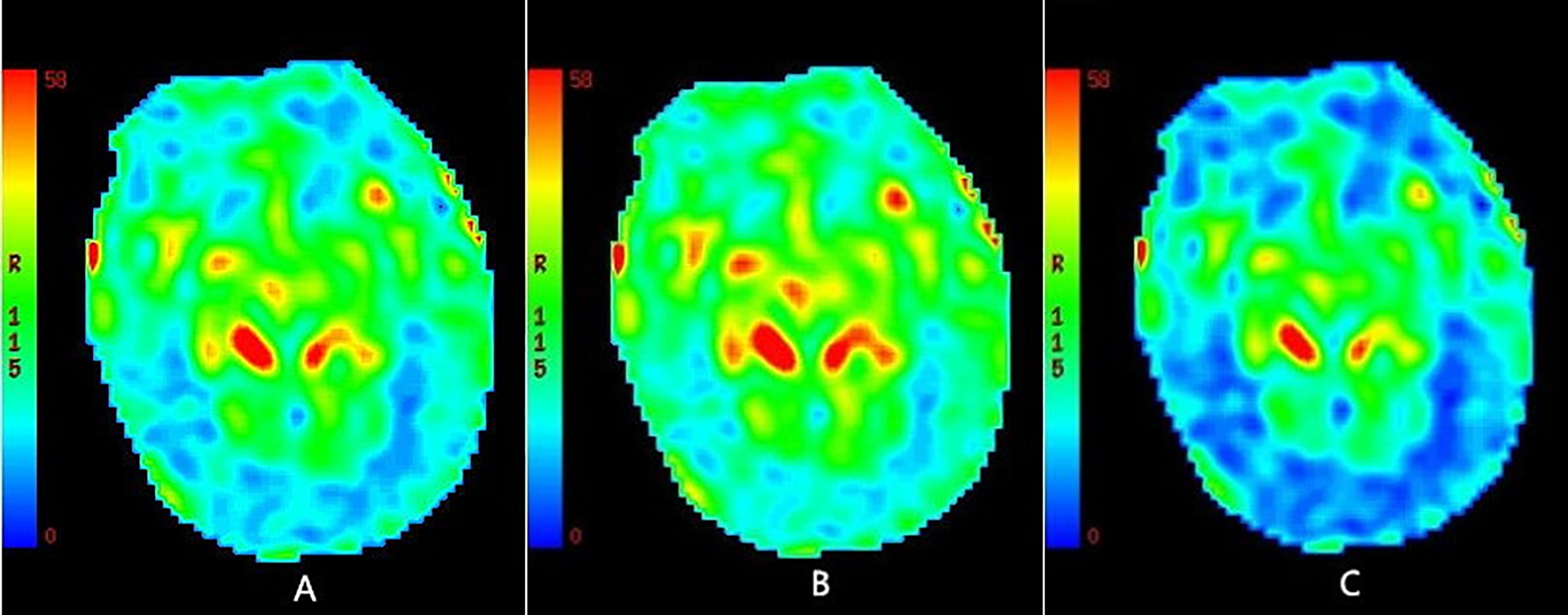

Supplement: S1 File — (ZIP) [file pone.0219284.s001.zip › patient CBF map/Figure6.tif]

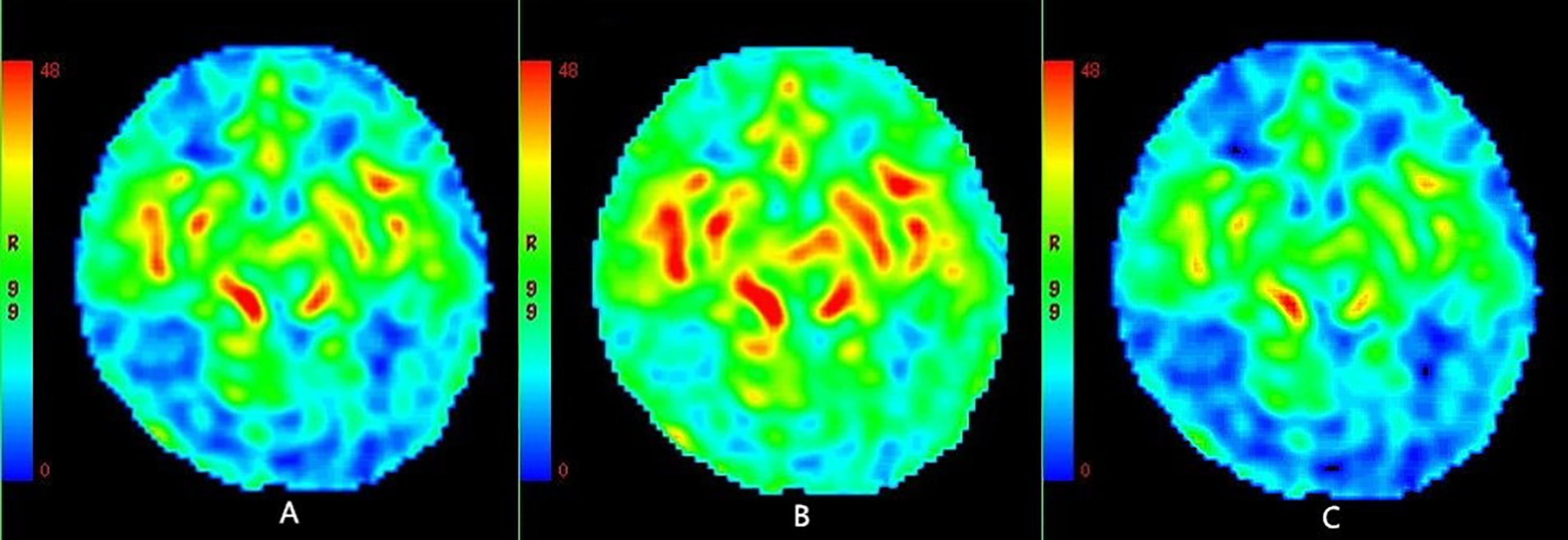

Supplement: S1 File — (ZIP) [file pone.0219284.s001.zip › patient CBF map/Figure7..tif]

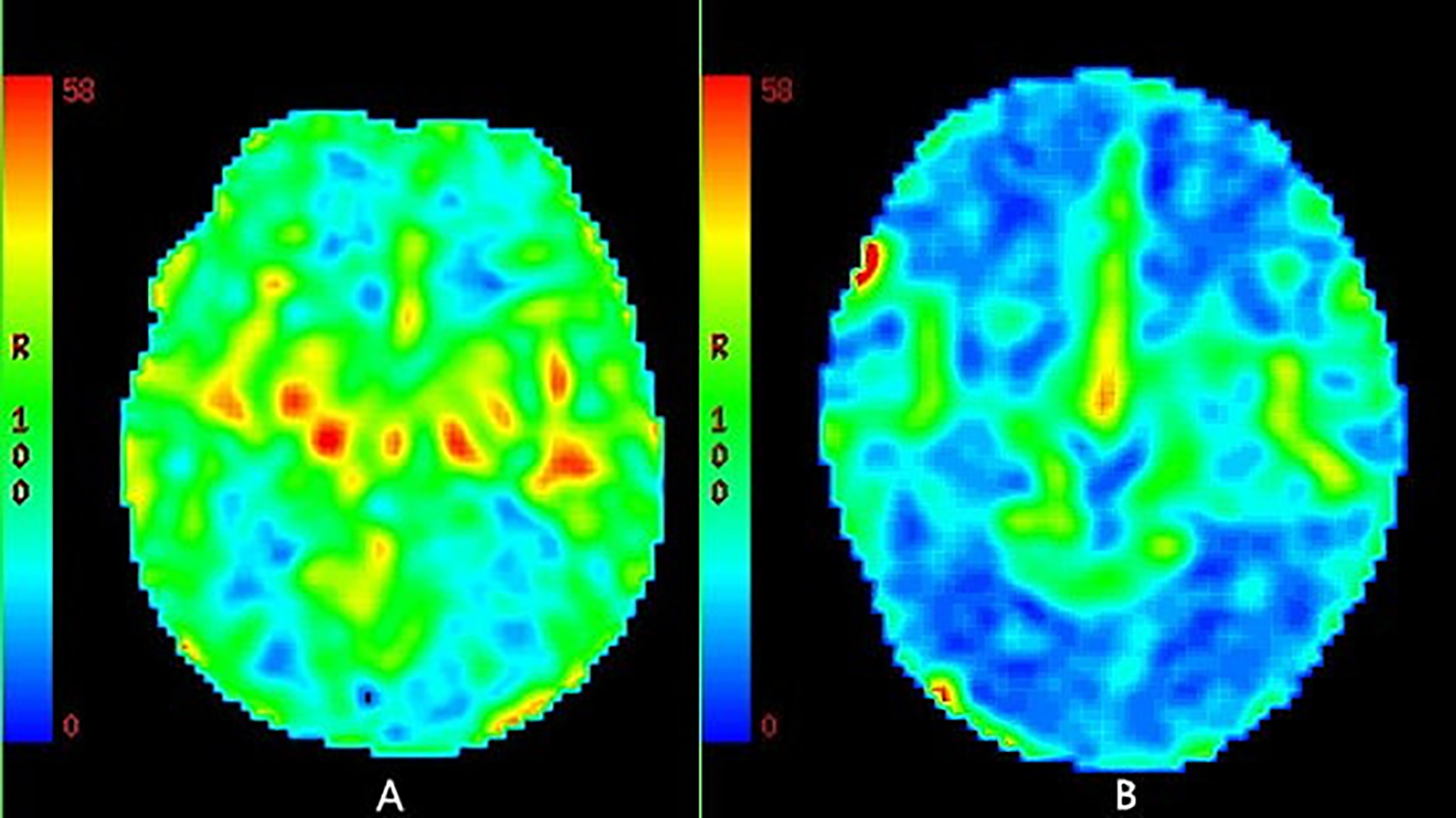

Supplement: S1 File — (ZIP) [file pone.0219284.s001.zip › patient CBF map/Figure9.tif]

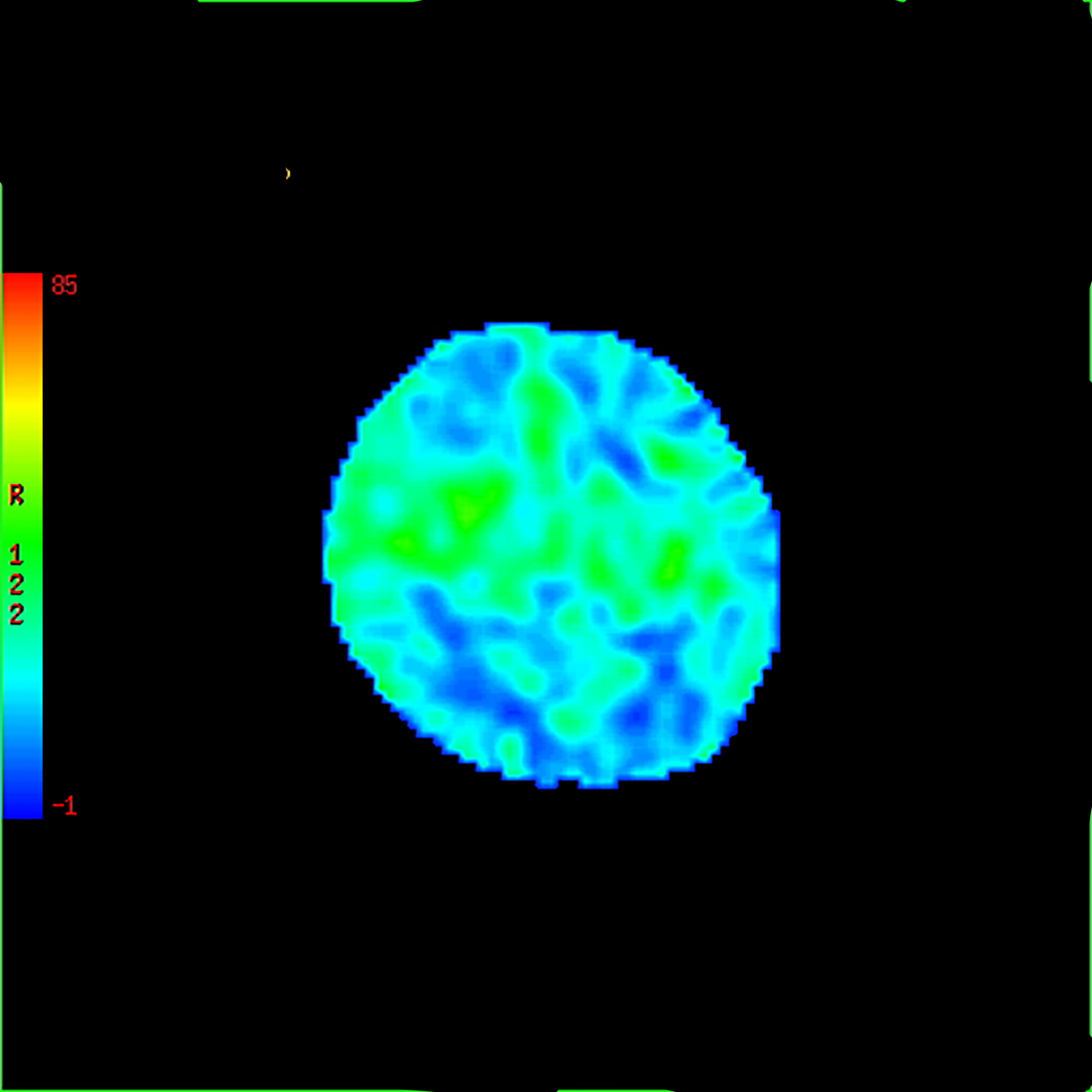

Supplement: S1 File — (ZIP) [file pone.0219284.s001.zip › patient CBF map/HIE1.tif]

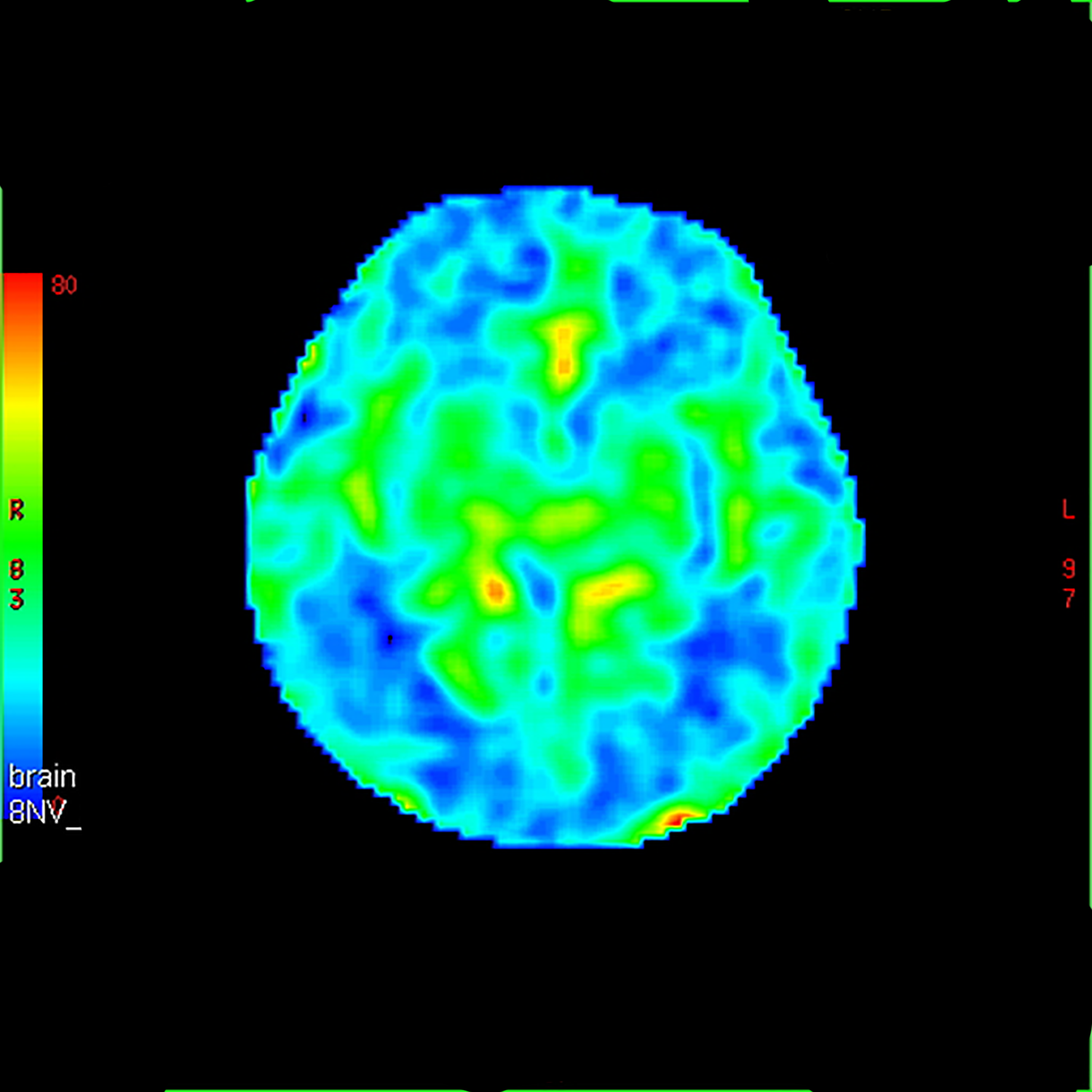

Supplement: S1 File — (ZIP) [file pone.0219284.s001.zip › patient CBF map/HIE10.tif]

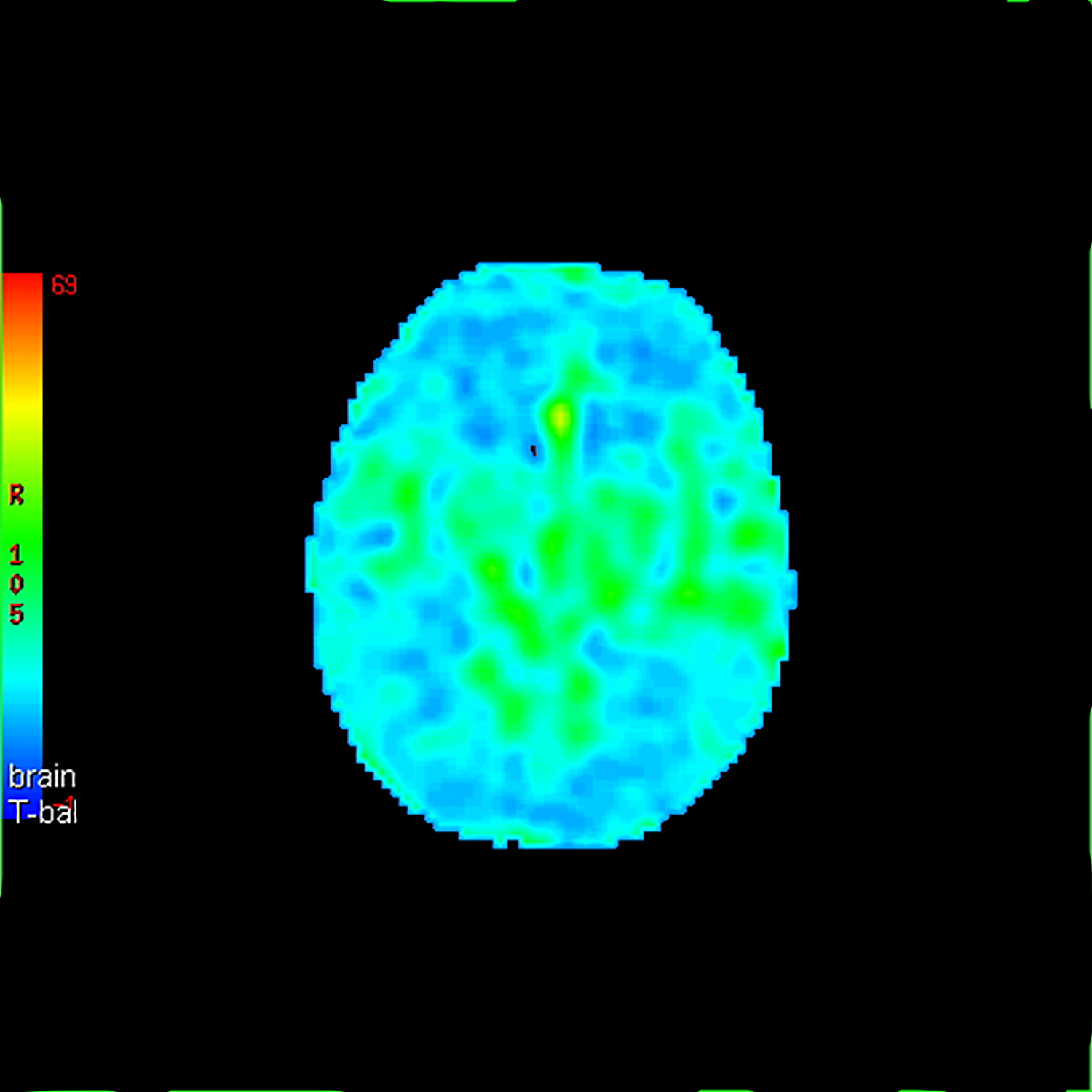

Supplement: S1 File — (ZIP) [file pone.0219284.s001.zip › patient CBF map/HIE11.tif]

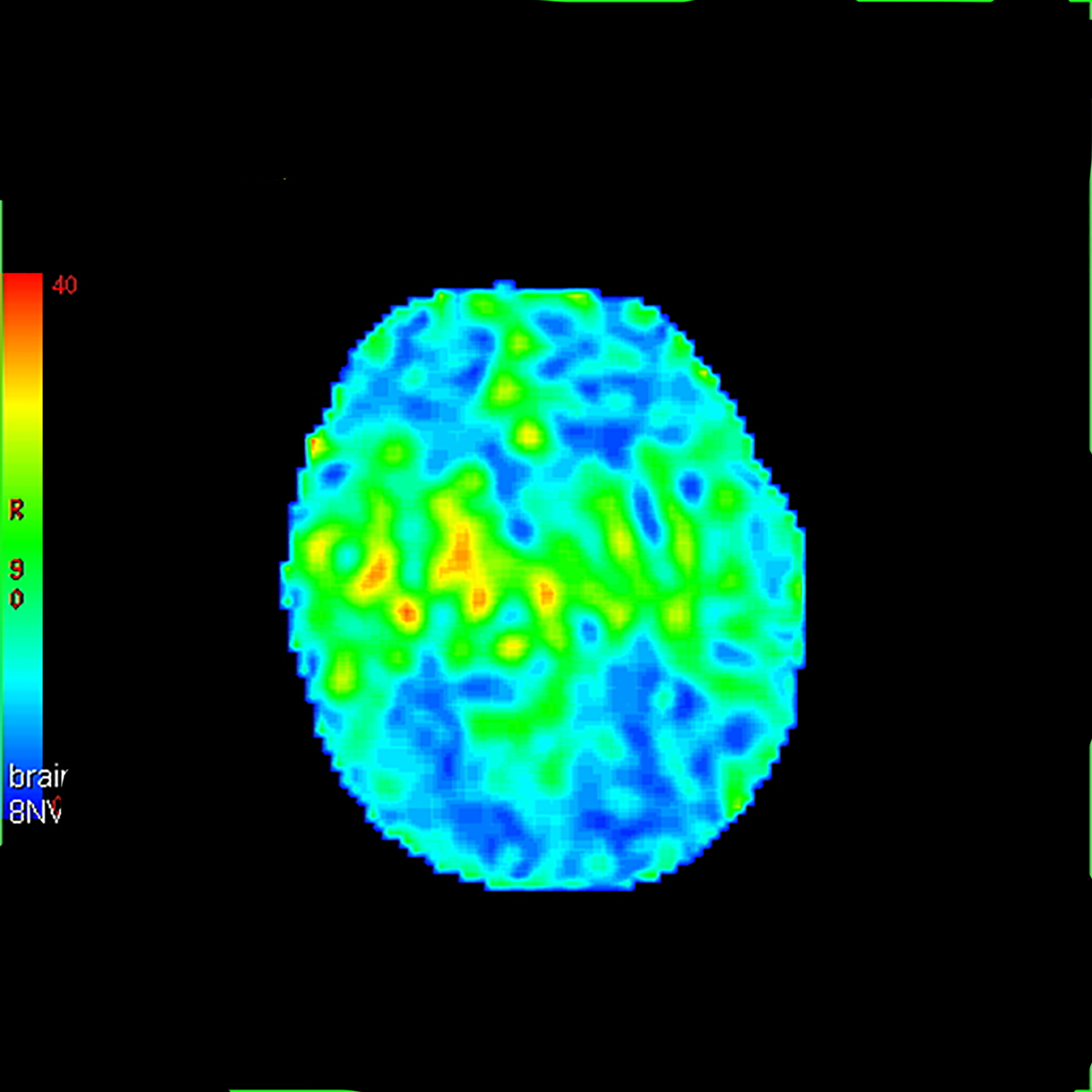

Supplement: S1 File — (ZIP) [file pone.0219284.s001.zip › patient CBF map/HIE12.tif]

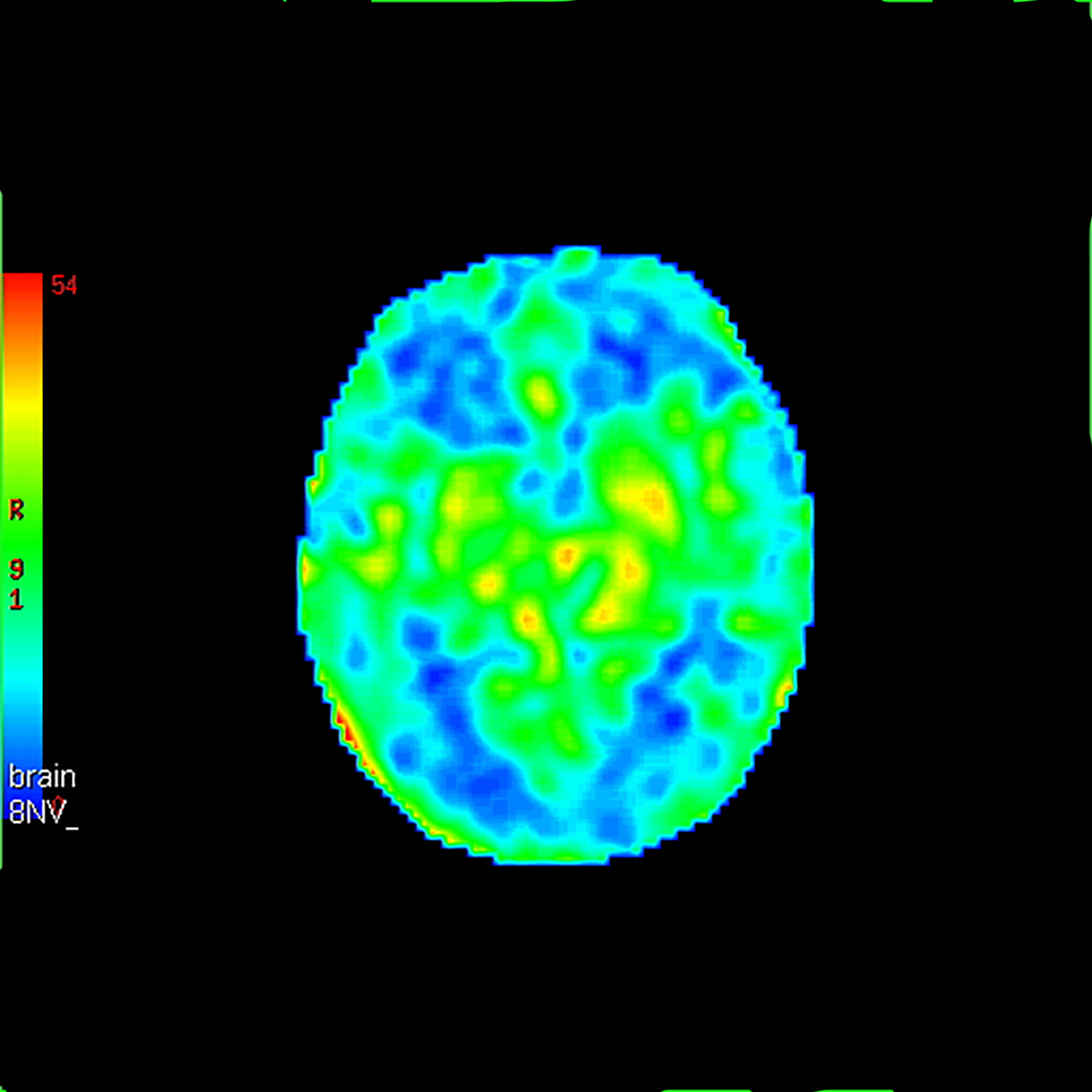

Supplement: S1 File — (ZIP) [file pone.0219284.s001.zip › patient CBF map/HIE13.tif]

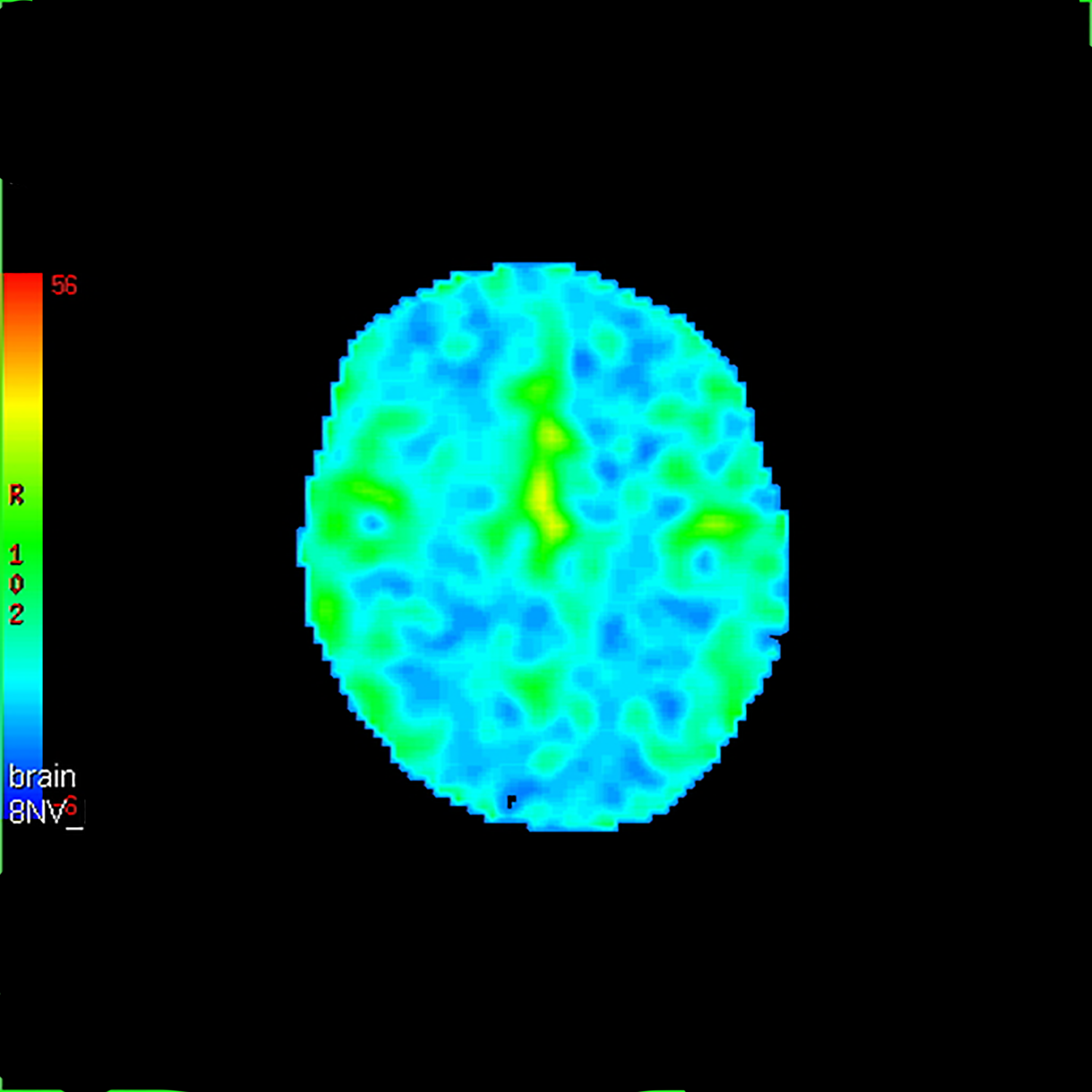

Supplement: S1 File — (ZIP) [file pone.0219284.s001.zip › patient CBF map/HIE14.tif]

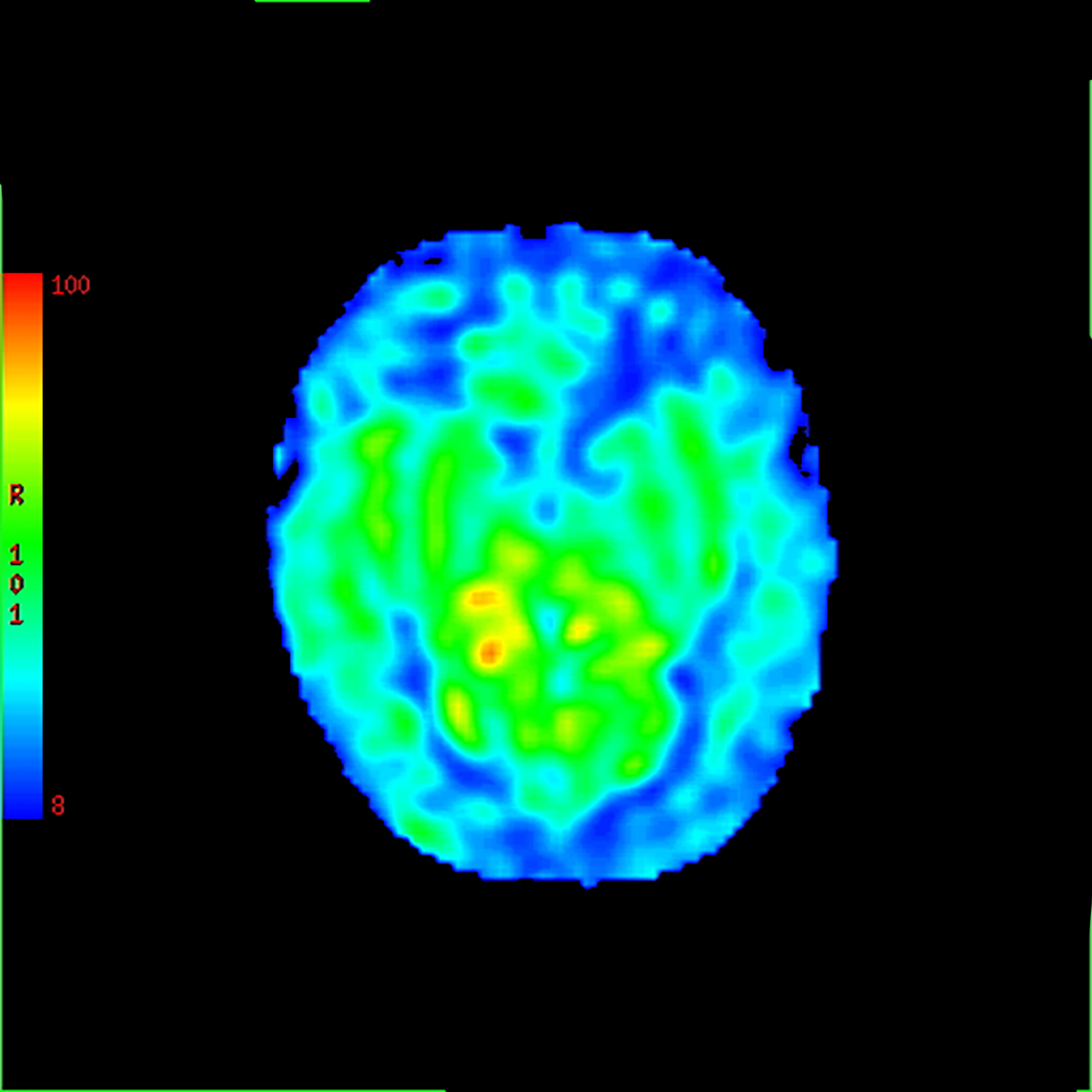

Supplement: S1 File — (ZIP) [file pone.0219284.s001.zip › patient CBF map/HIE15.tif]

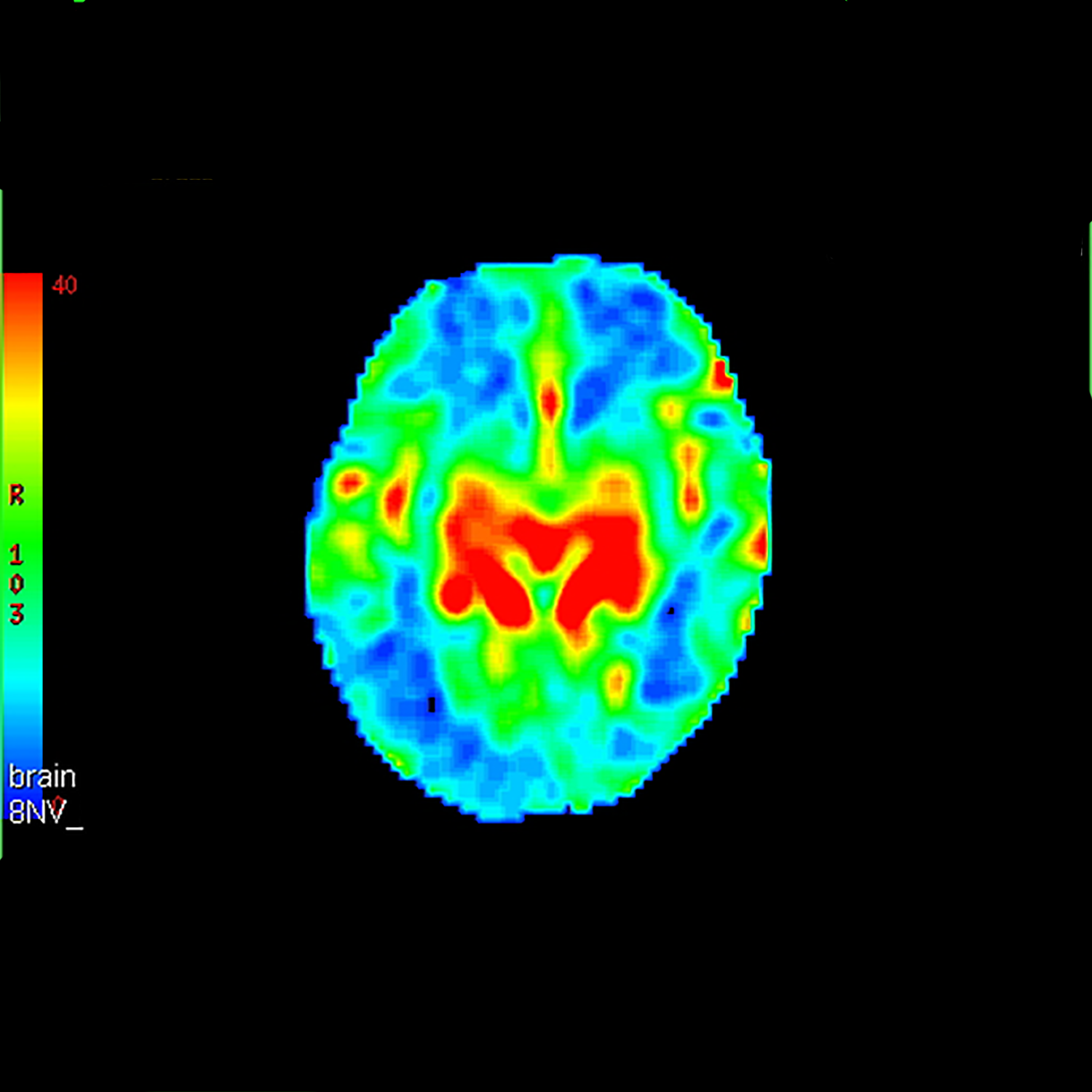

Supplement: S1 File — (ZIP) [file pone.0219284.s001.zip › patient CBF map/HIE16.tif]

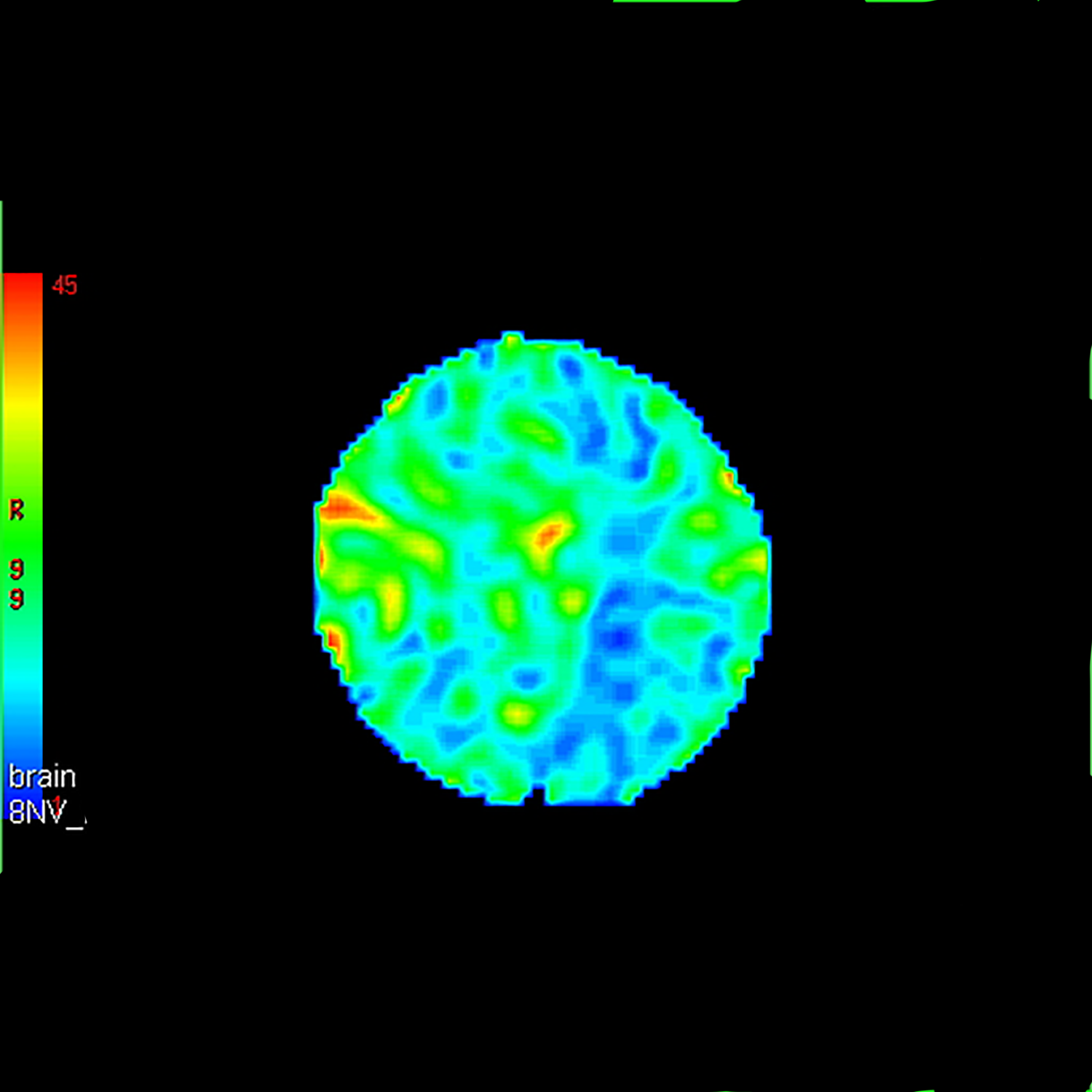

Supplement: S1 File — (ZIP) [file pone.0219284.s001.zip › patient CBF map/HIE17.tif]

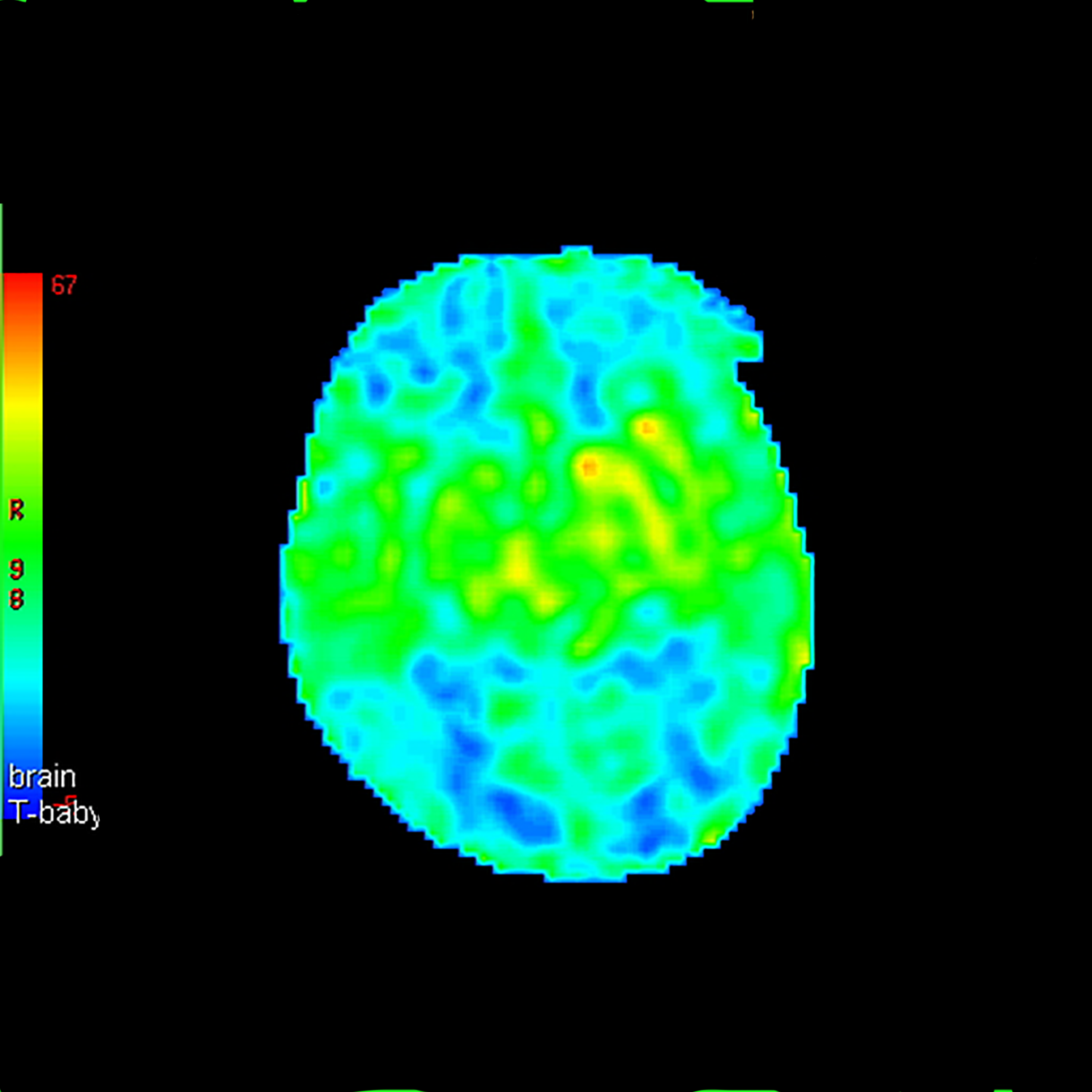

Supplement: S1 File — (ZIP) [file pone.0219284.s001.zip › patient CBF map/HIE18.tif]

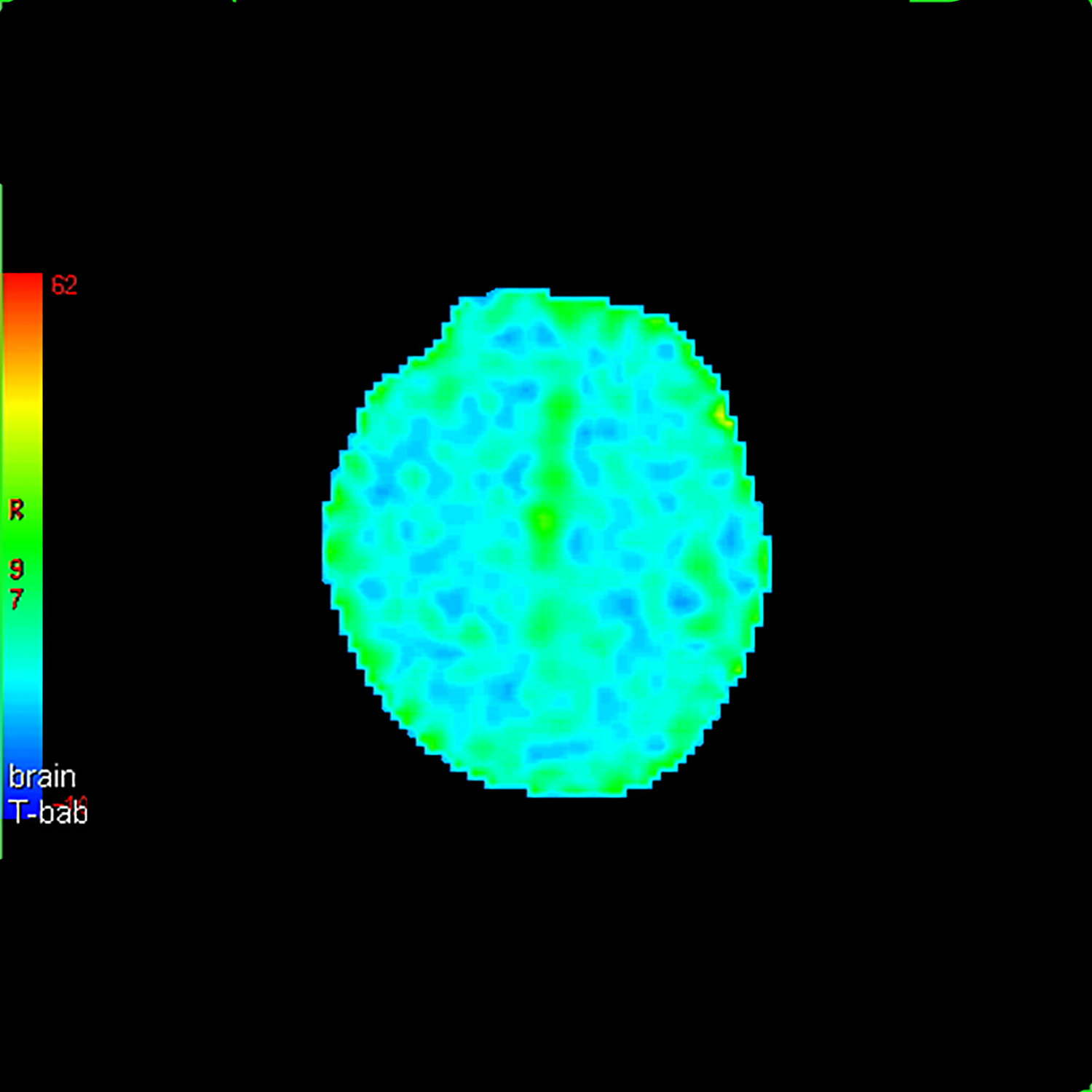

Supplement: S1 File — (ZIP) [file pone.0219284.s001.zip › patient CBF map/HIE19.tif]

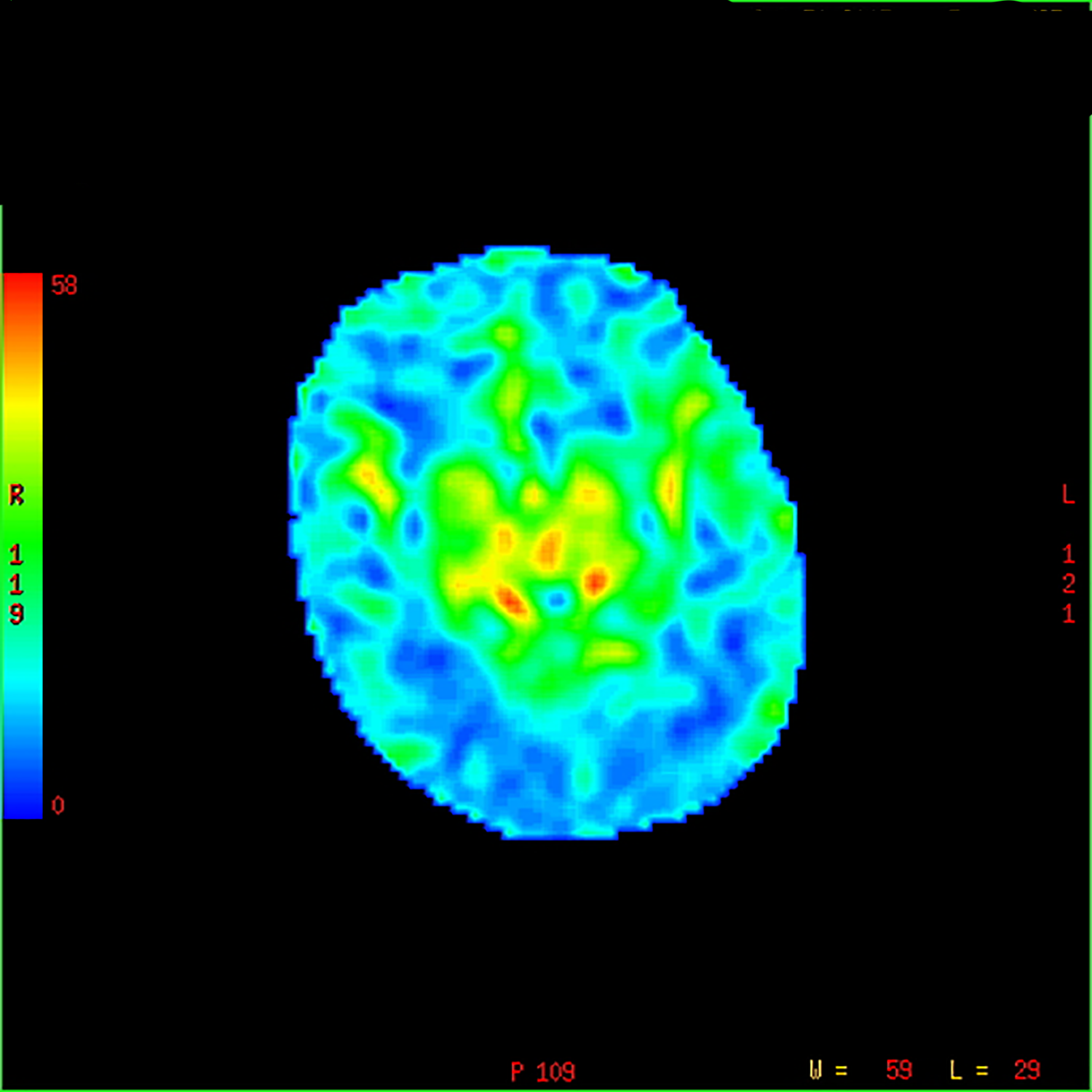

Supplement: S1 File — (ZIP) [file pone.0219284.s001.zip › patient CBF map/HIE2.tif]

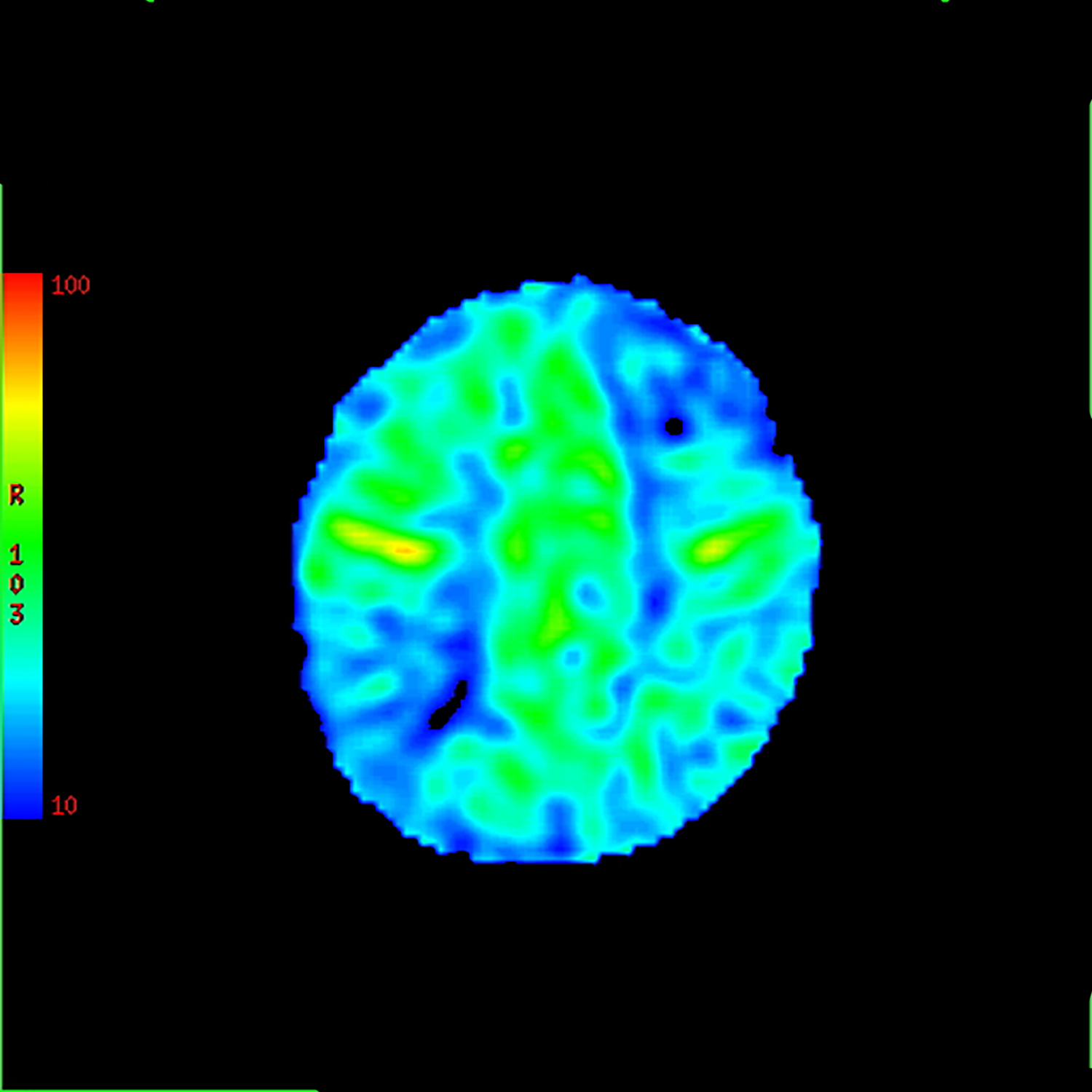

Supplement: S1 File — (ZIP) [file pone.0219284.s001.zip › patient CBF map/HIE20.tif]

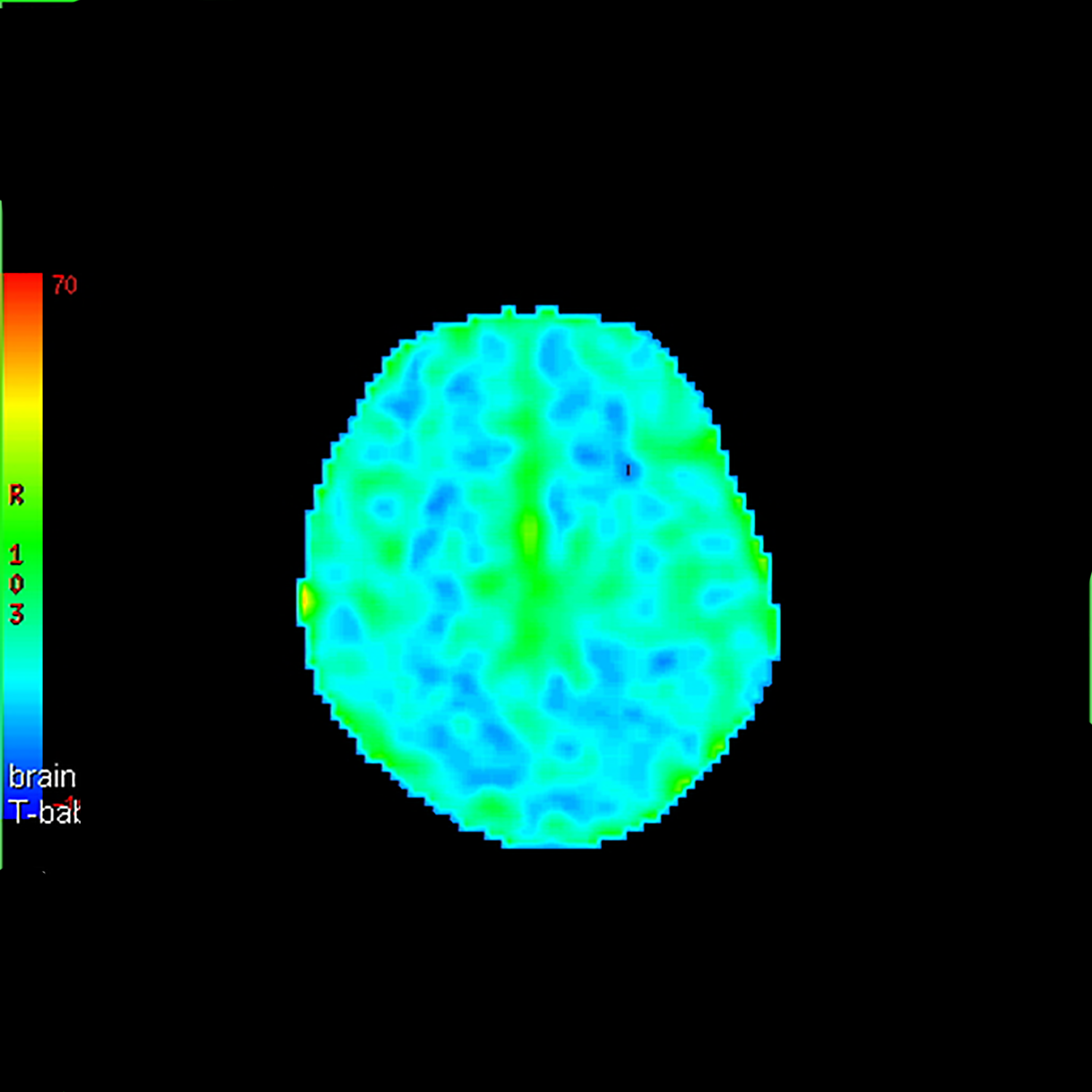

Supplement: S1 File — (ZIP) [file pone.0219284.s001.zip › patient CBF map/HIE21.tif]

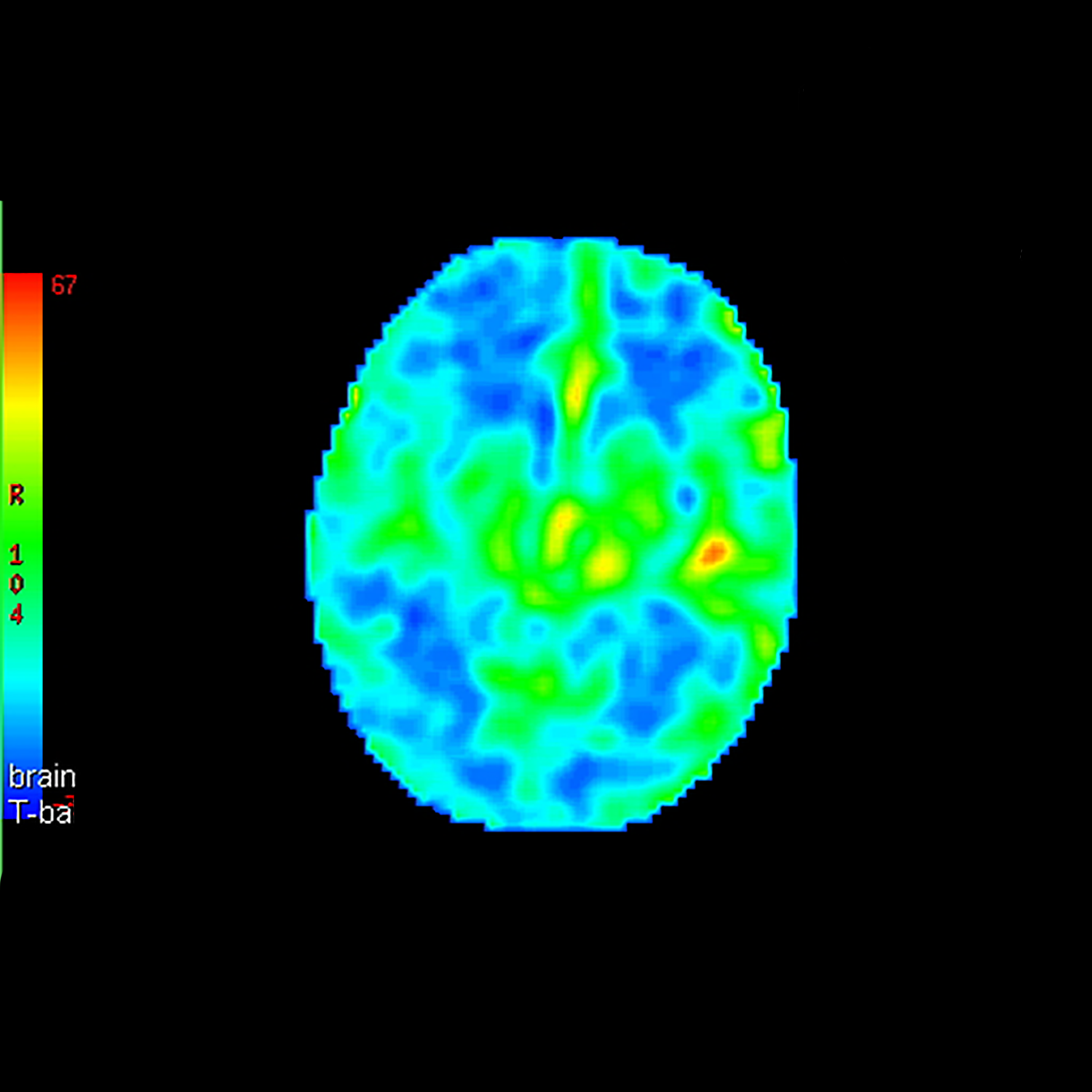

Supplement: S1 File — (ZIP) [file pone.0219284.s001.zip › patient CBF map/HIE22.tif]

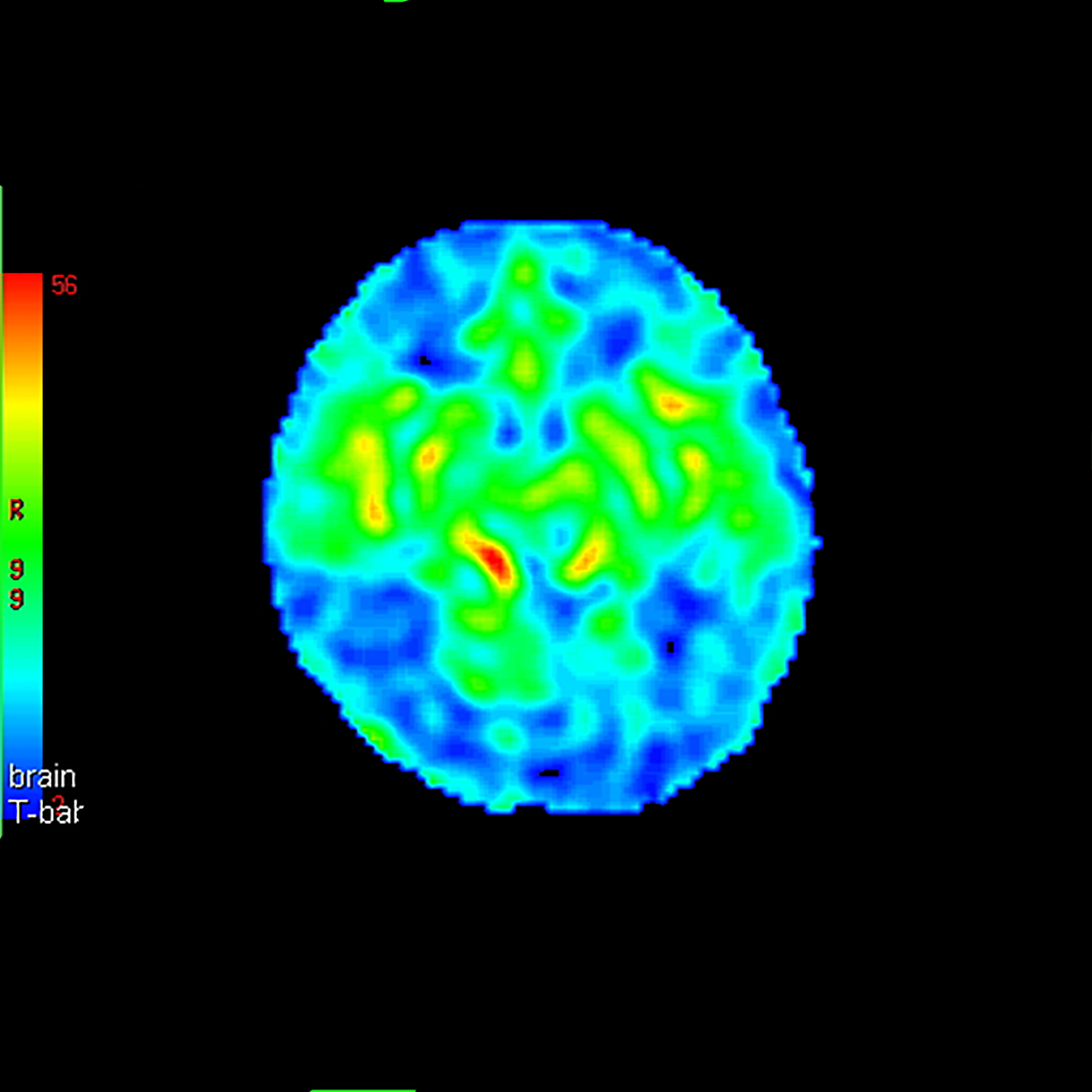

Supplement: S1 File — (ZIP) [file pone.0219284.s001.zip › patient CBF map/HIE23.tif]

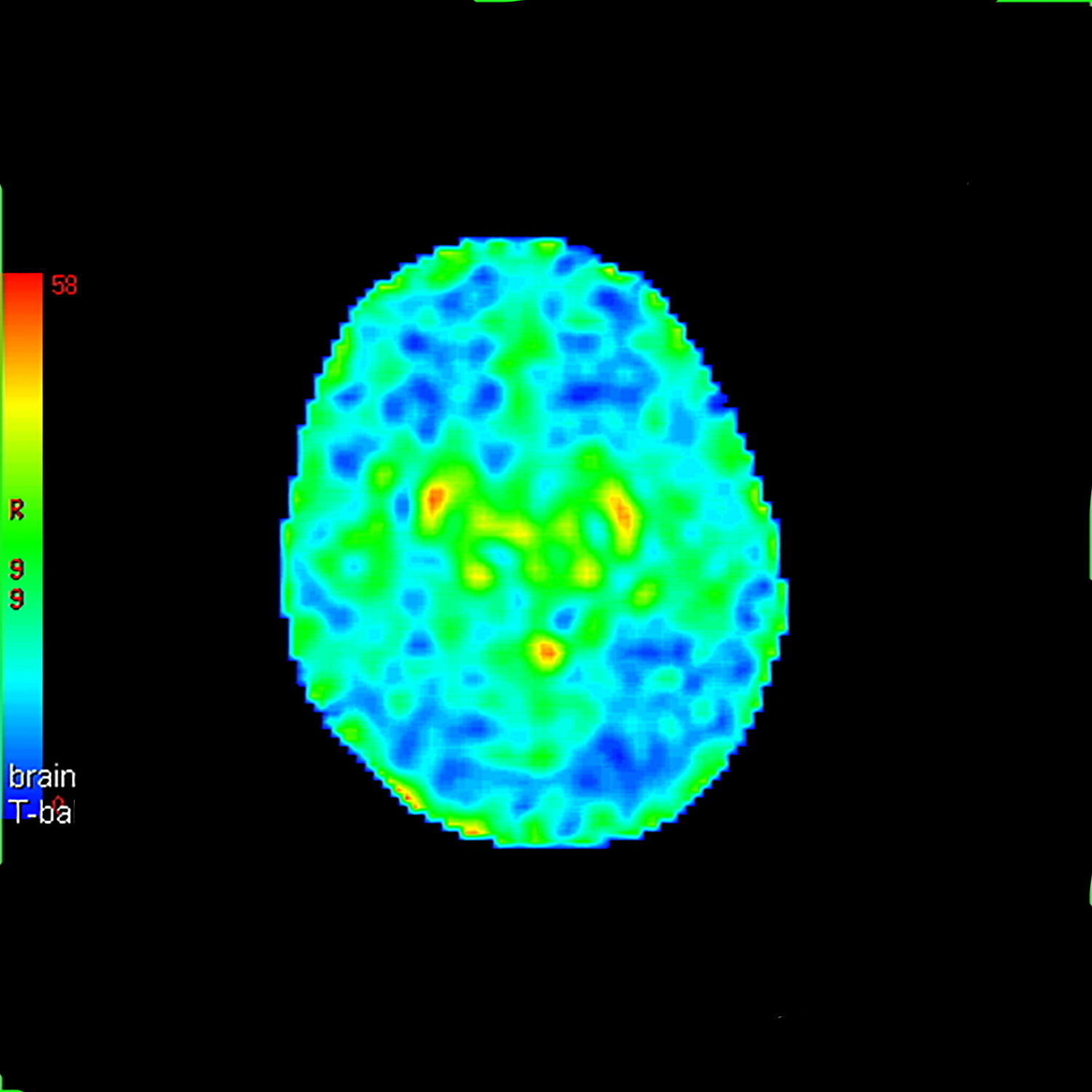

Supplement: S1 File — (ZIP) [file pone.0219284.s001.zip › patient CBF map/HIE24.tif]

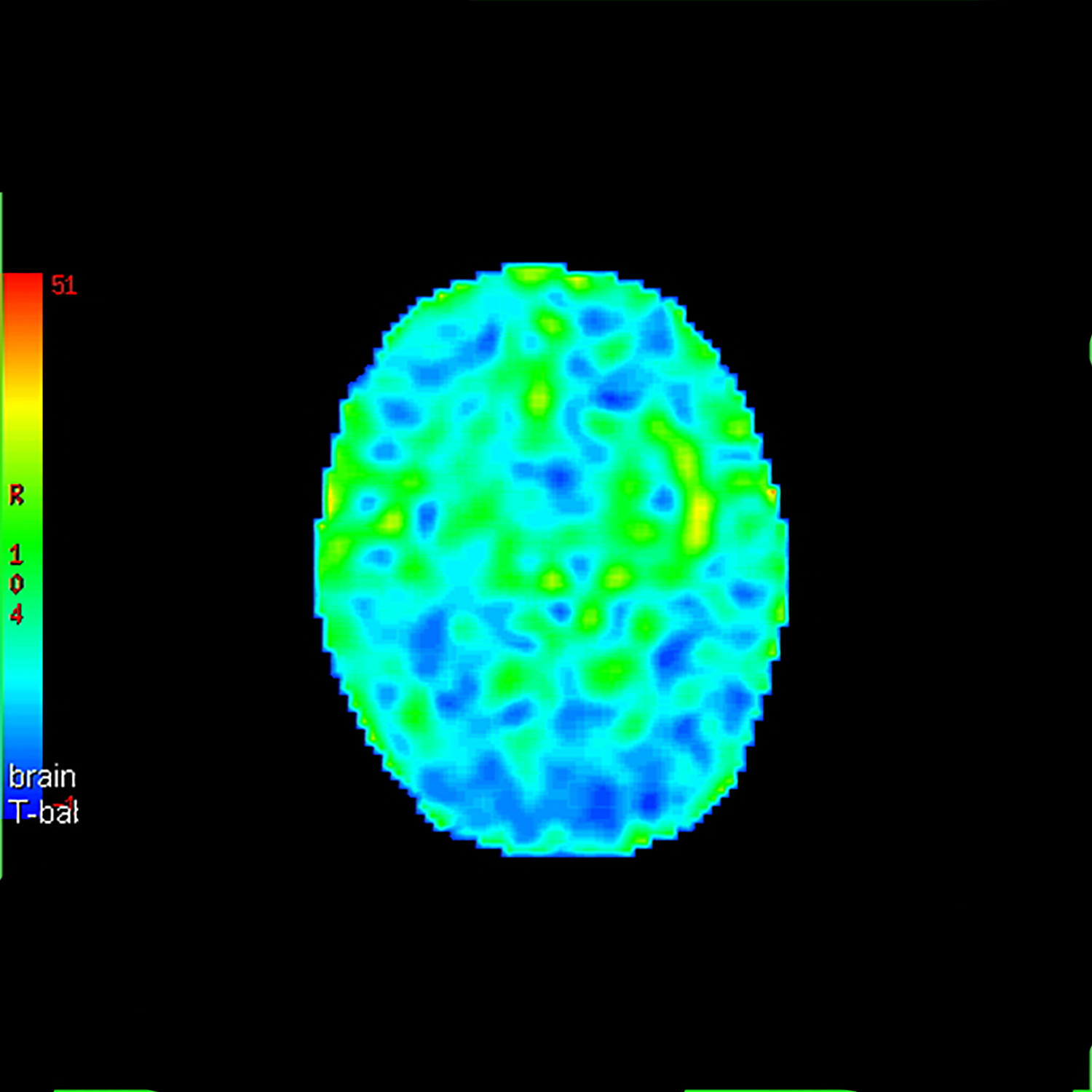

Supplement: S1 File — (ZIP) [file pone.0219284.s001.zip › patient CBF map/HIE25.tif]

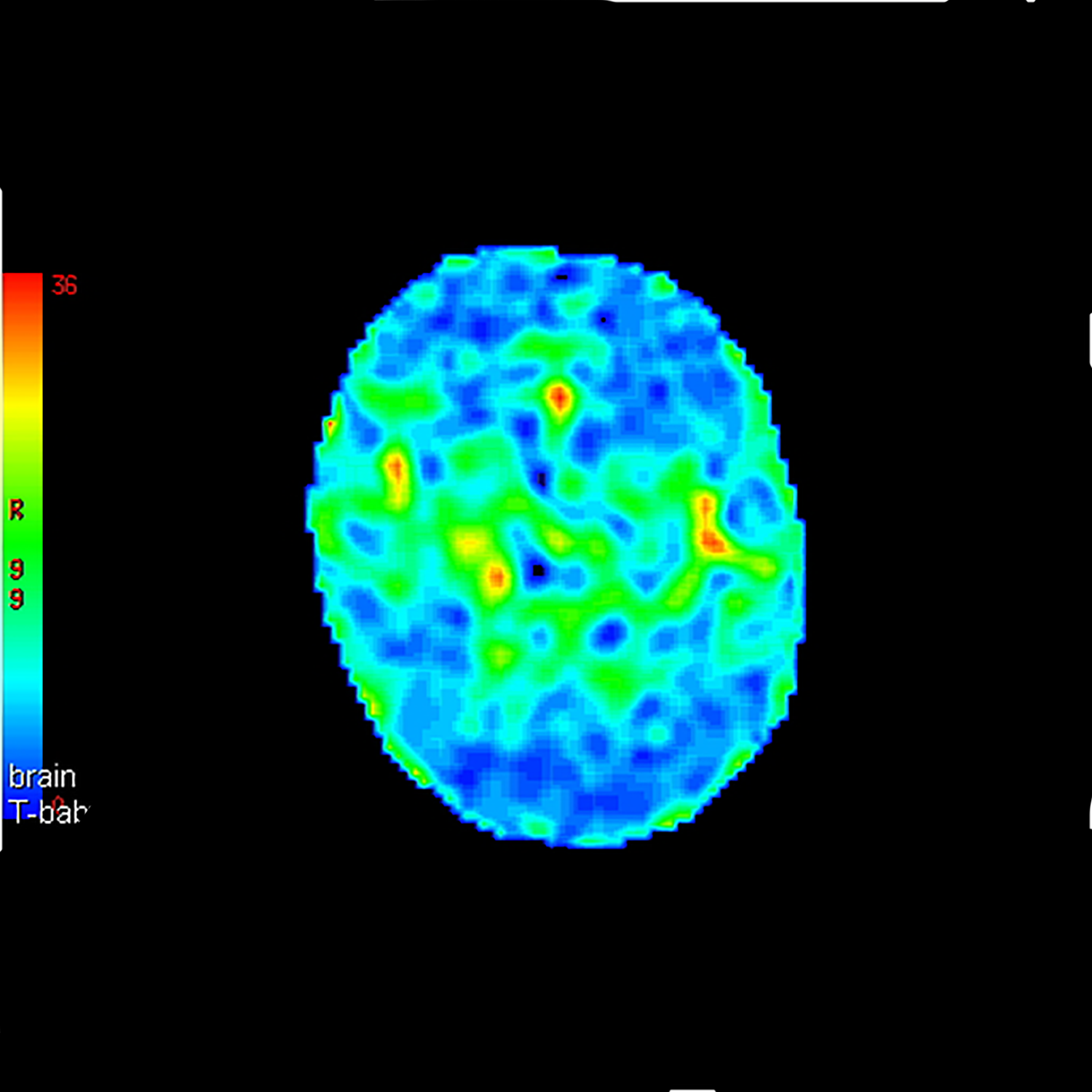

Supplement: S1 File — (ZIP) [file pone.0219284.s001.zip › patient CBF map/HIE26.tif]

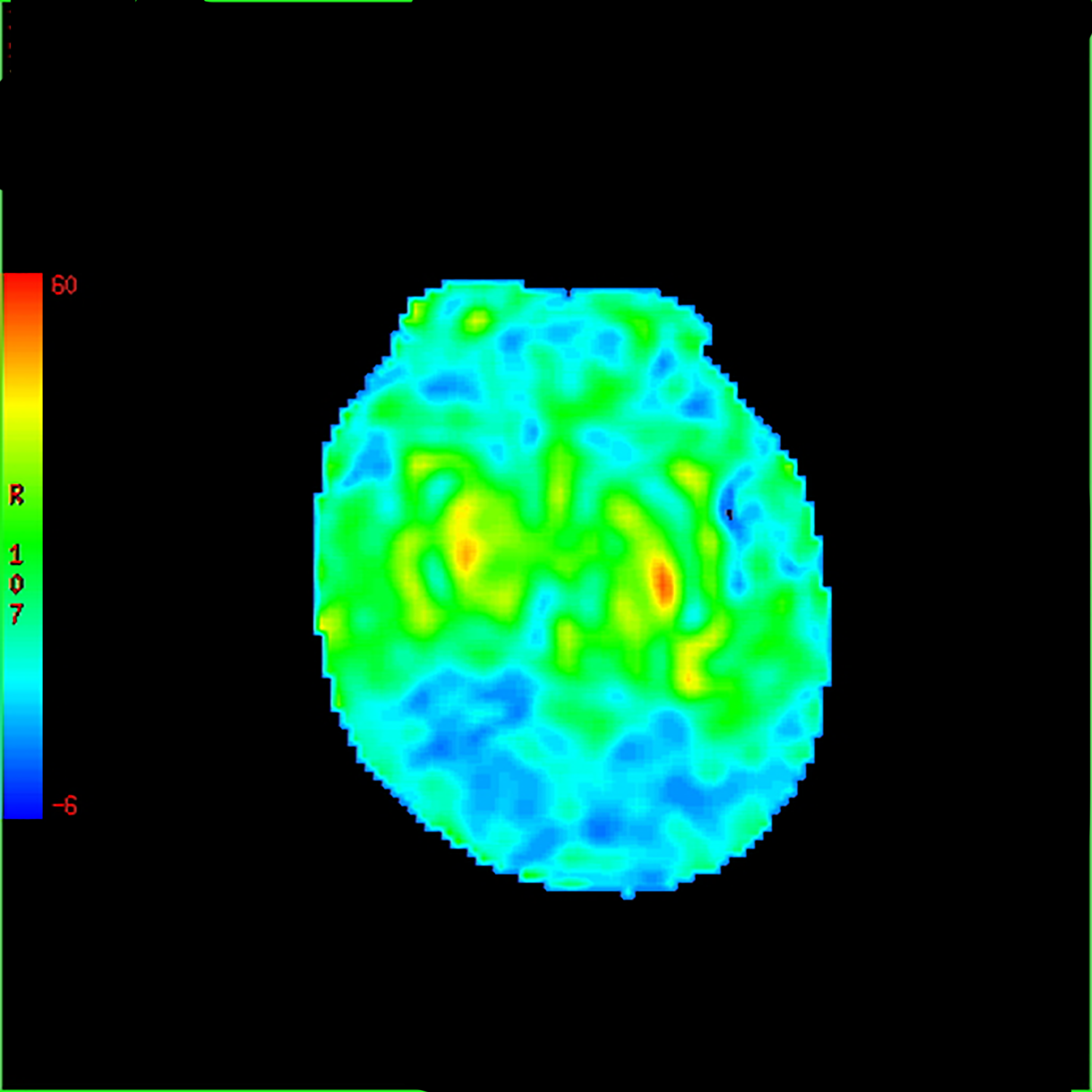

Supplement: S1 File — (ZIP) [file pone.0219284.s001.zip › patient CBF map/HIE27.tif]

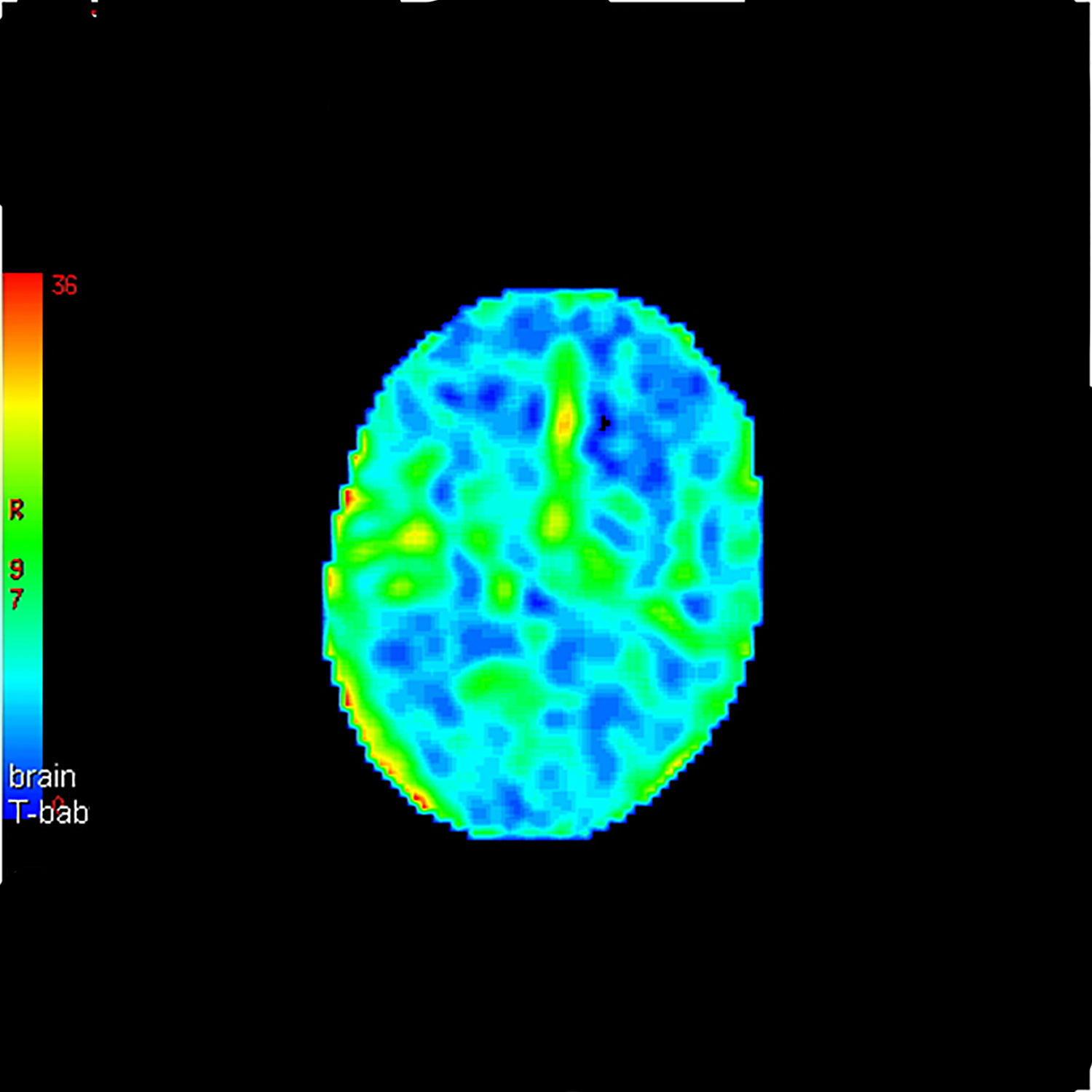

Supplement: S1 File — (ZIP) [file pone.0219284.s001.zip › patient CBF map/HIE28.tif]

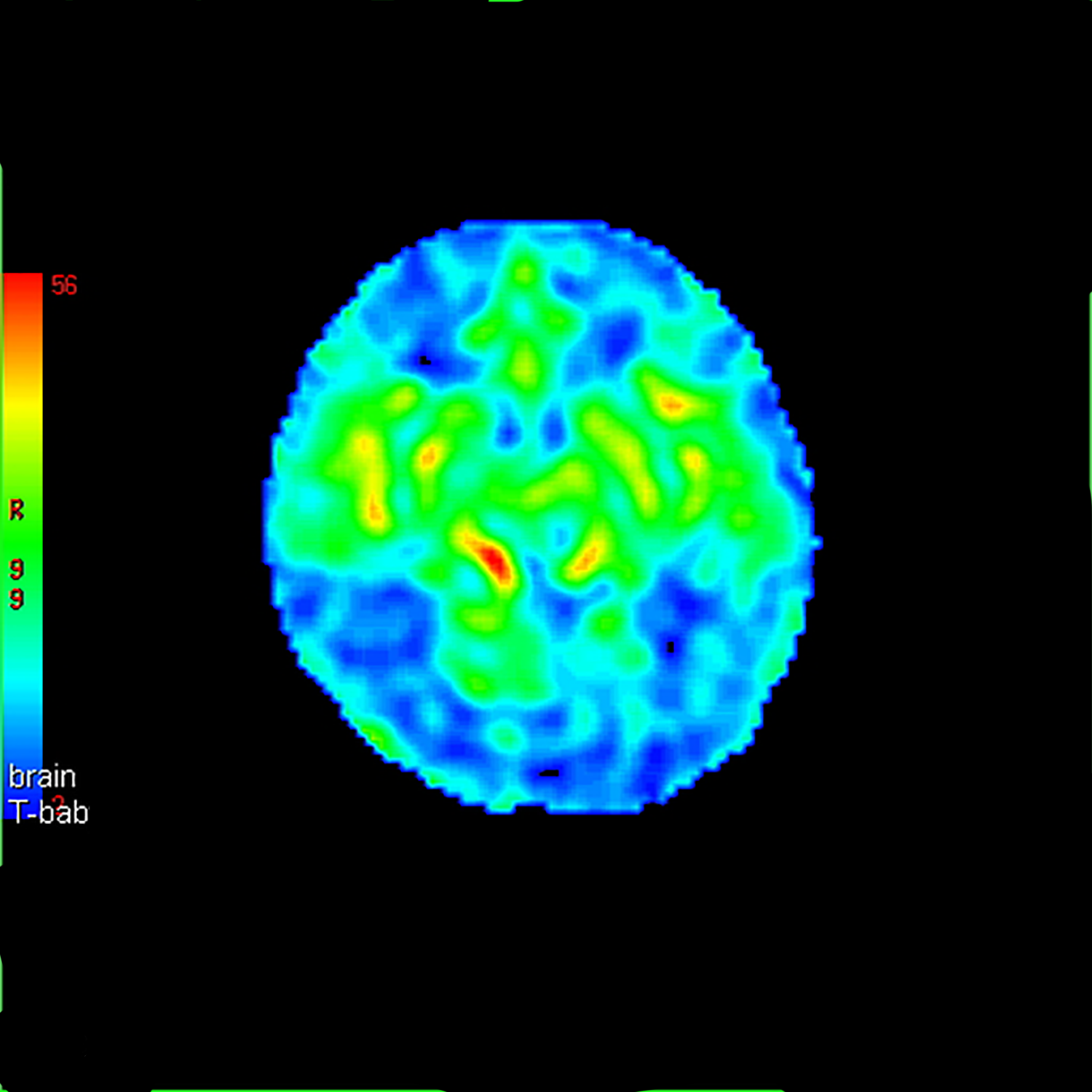

Supplement: S1 File — (ZIP) [file pone.0219284.s001.zip › patient CBF map/HIE29.tif]

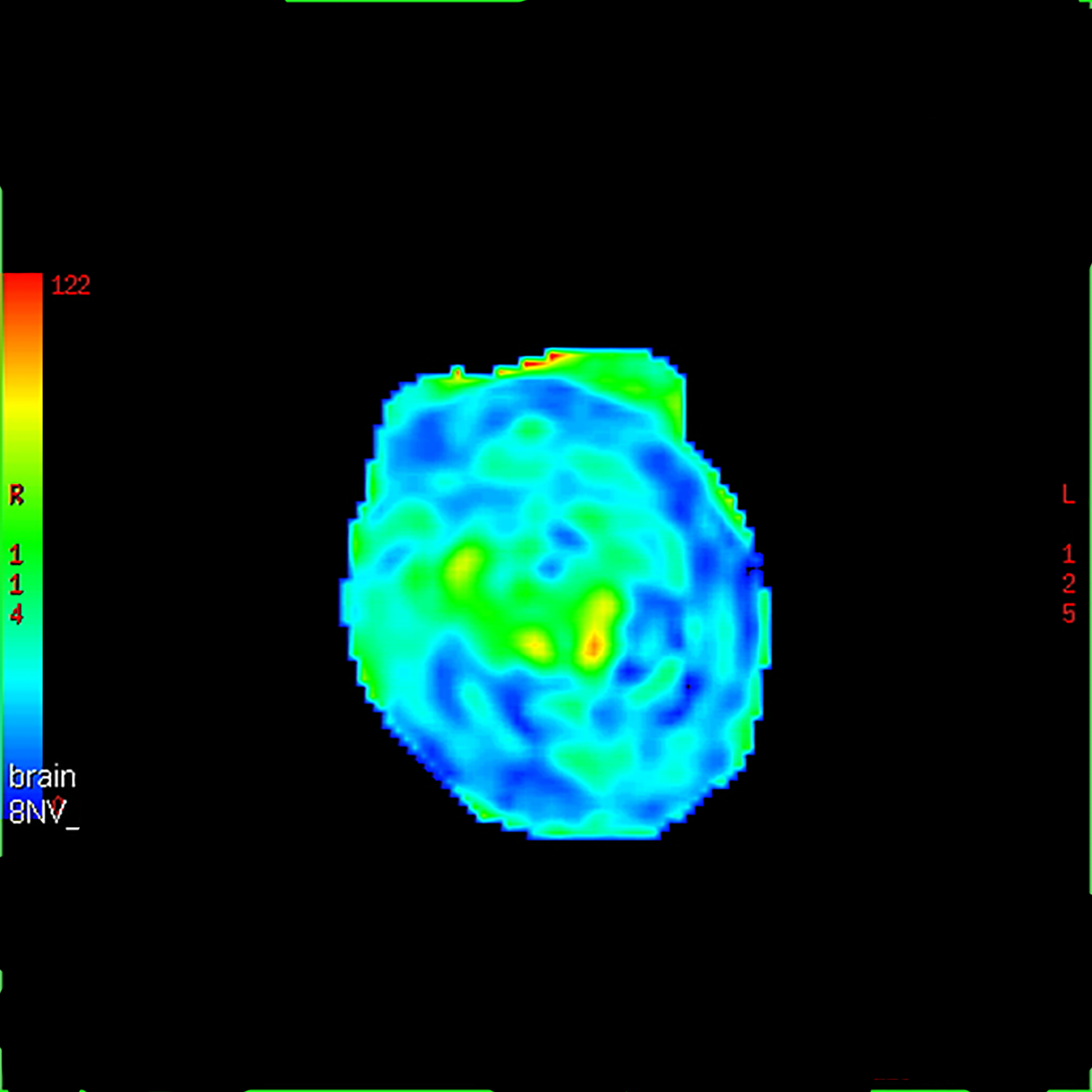

Supplement: S1 File — (ZIP) [file pone.0219284.s001.zip › patient CBF map/HIE3.tif]

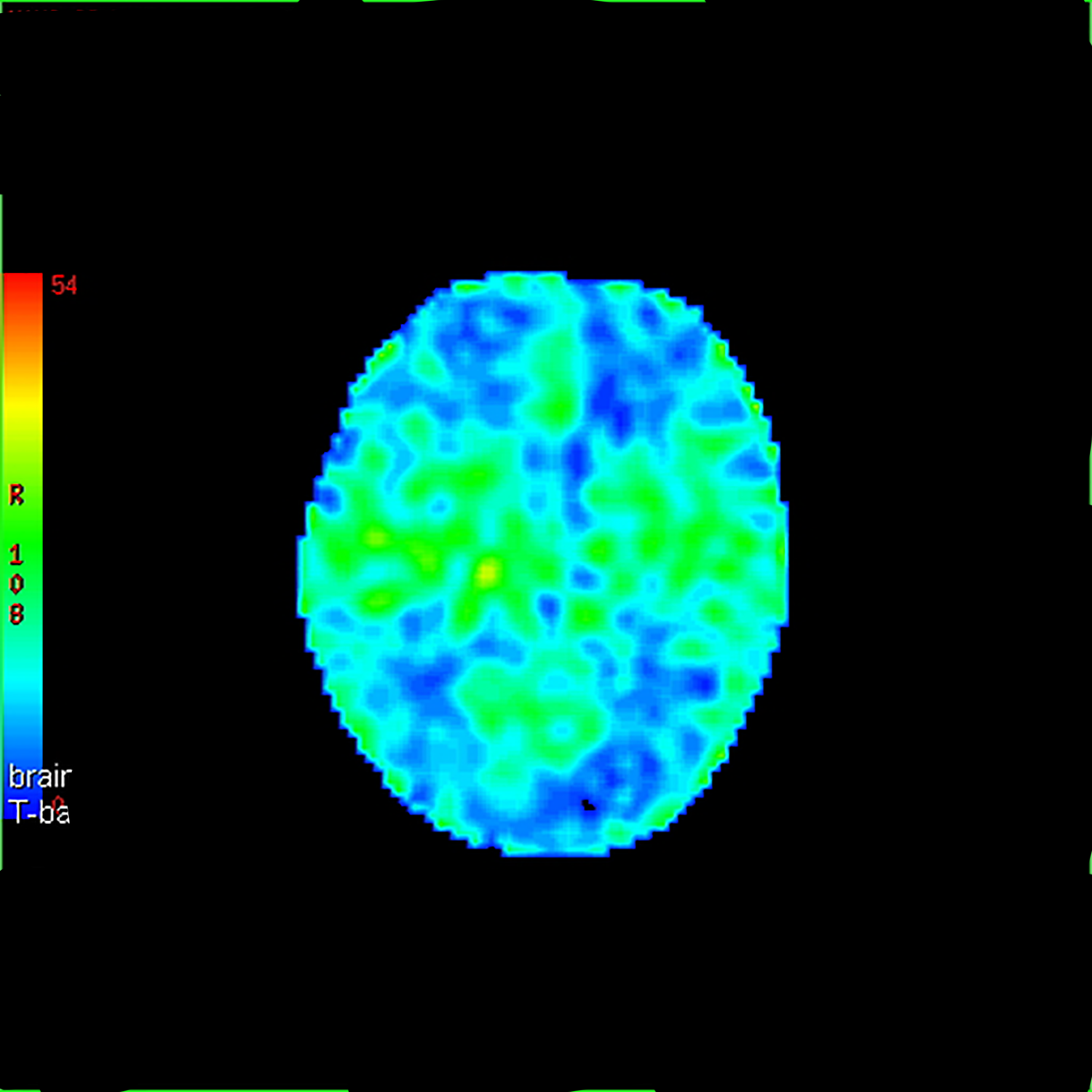

Supplement: S1 File — (ZIP) [file pone.0219284.s001.zip › patient CBF map/HIE30.tif]

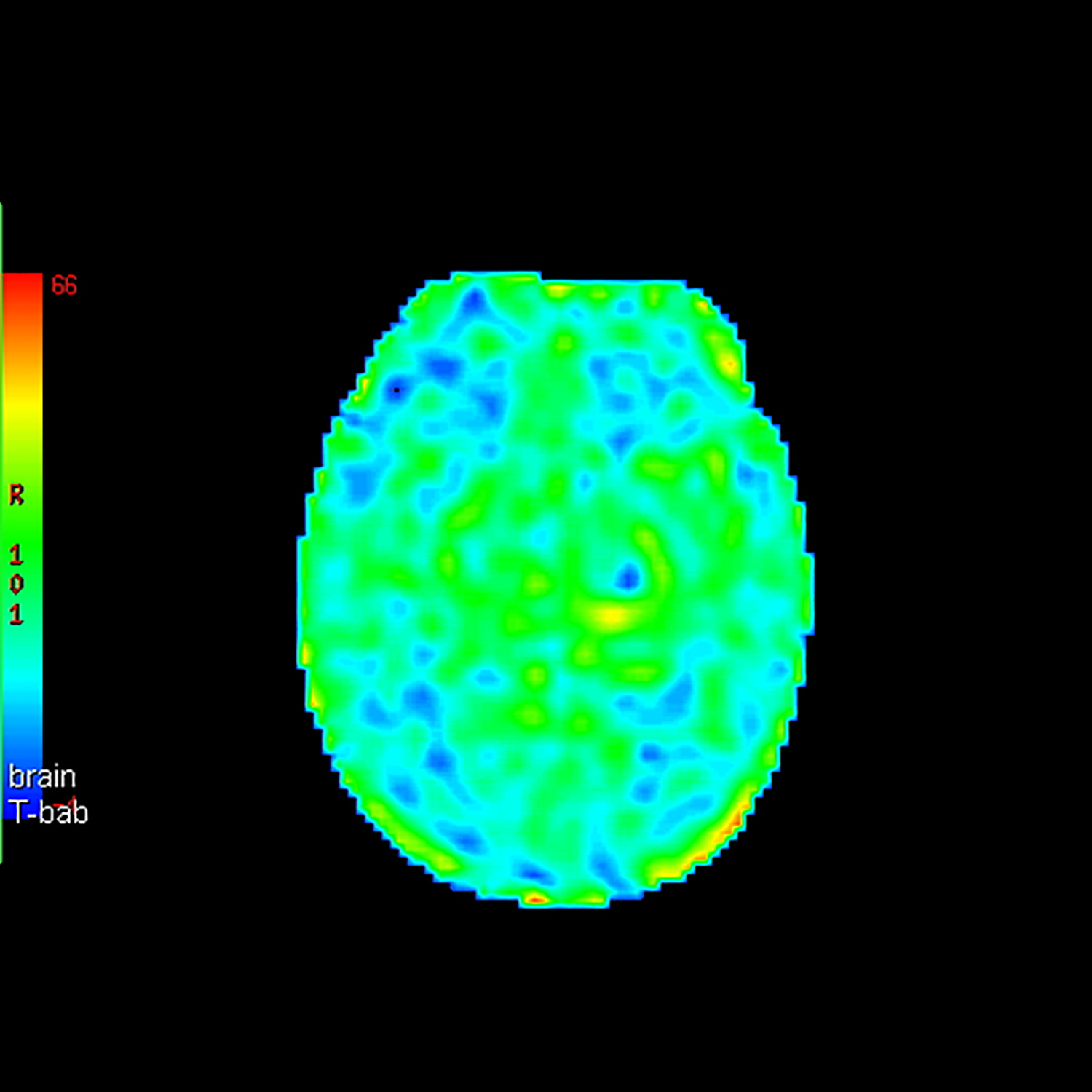

Supplement: S1 File — (ZIP) [file pone.0219284.s001.zip › patient CBF map/HIE31.tif]

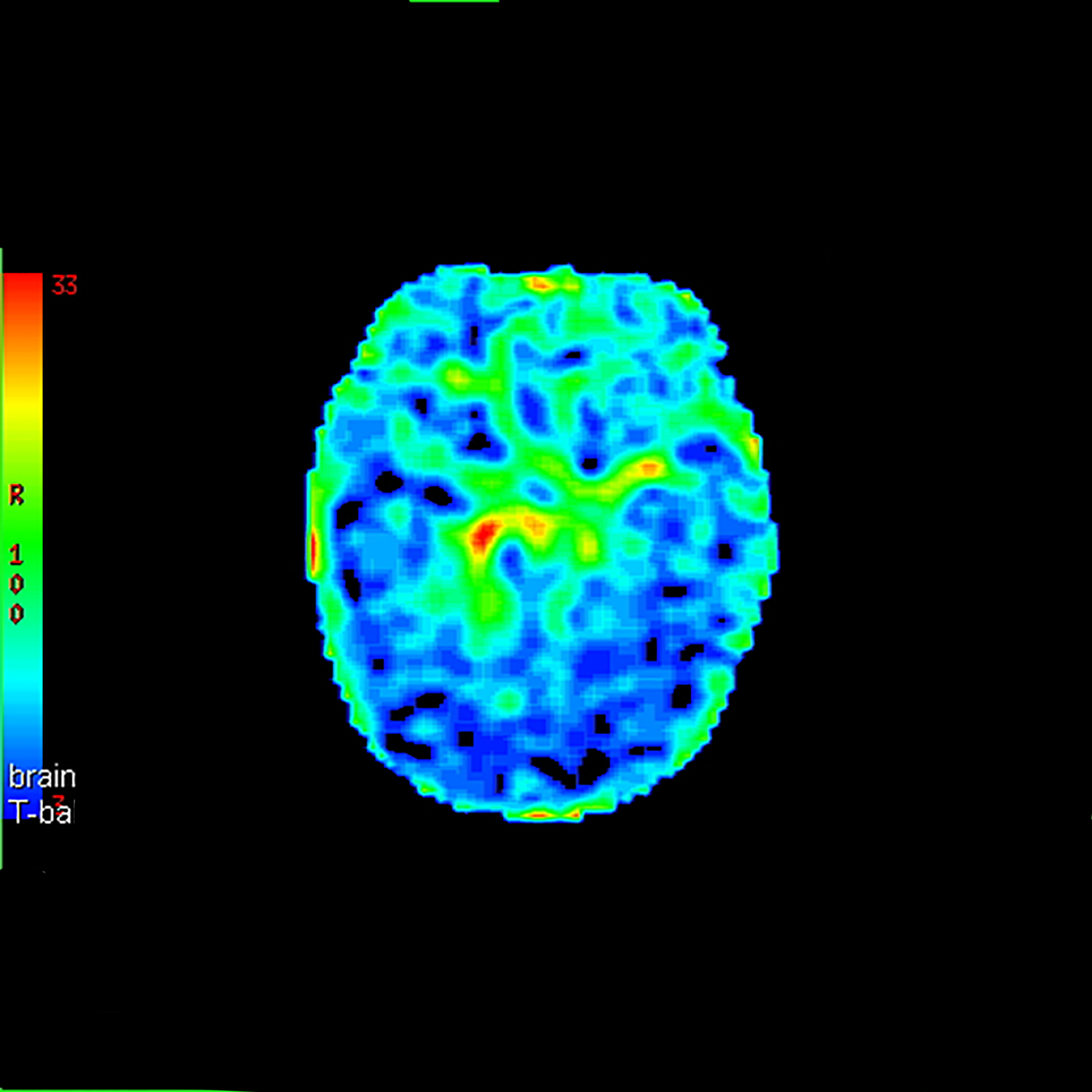

Supplement: S1 File — (ZIP) [file pone.0219284.s001.zip › patient CBF map/HIE32.tif]

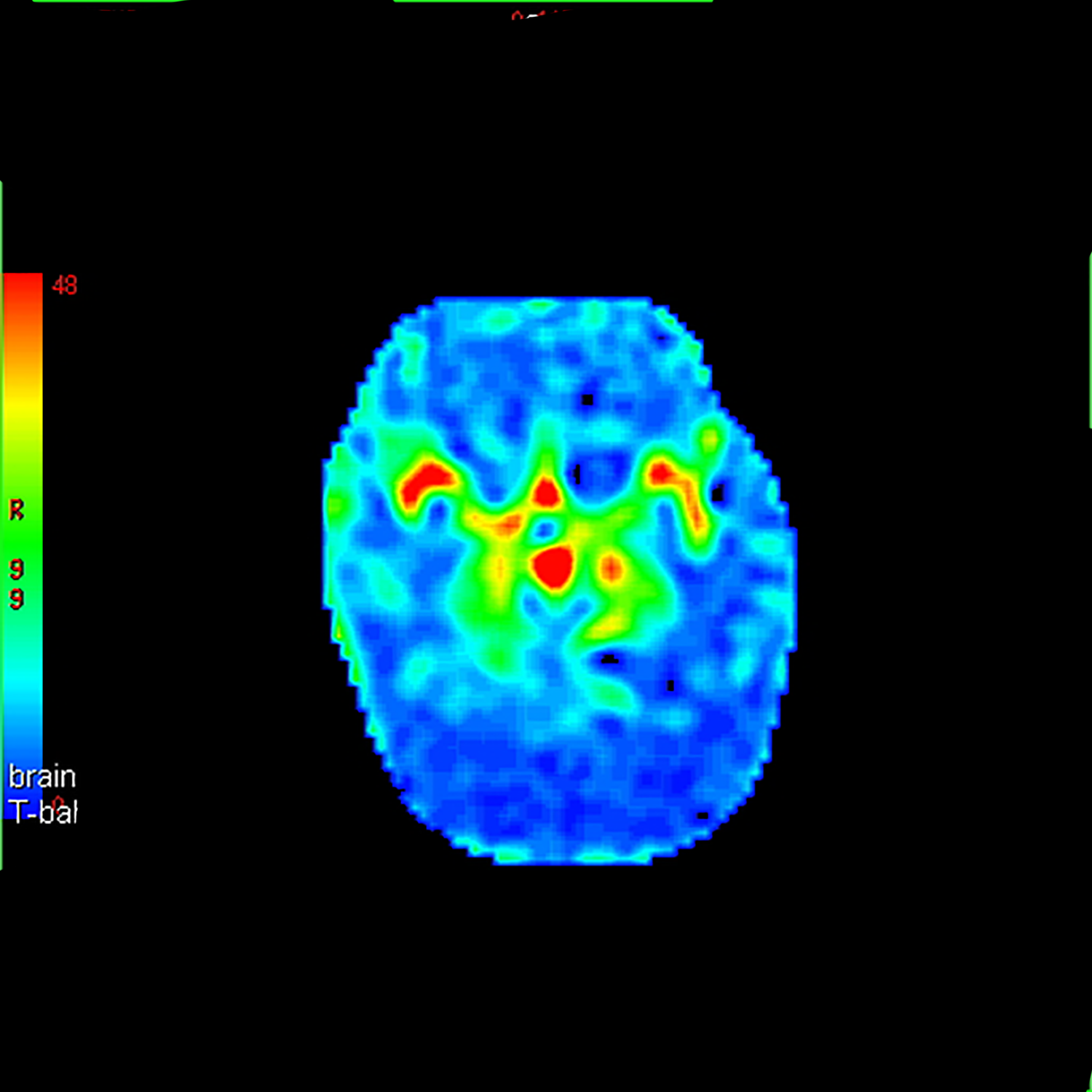

Supplement: S1 File — (ZIP) [file pone.0219284.s001.zip › patient CBF map/HIE33.tif]

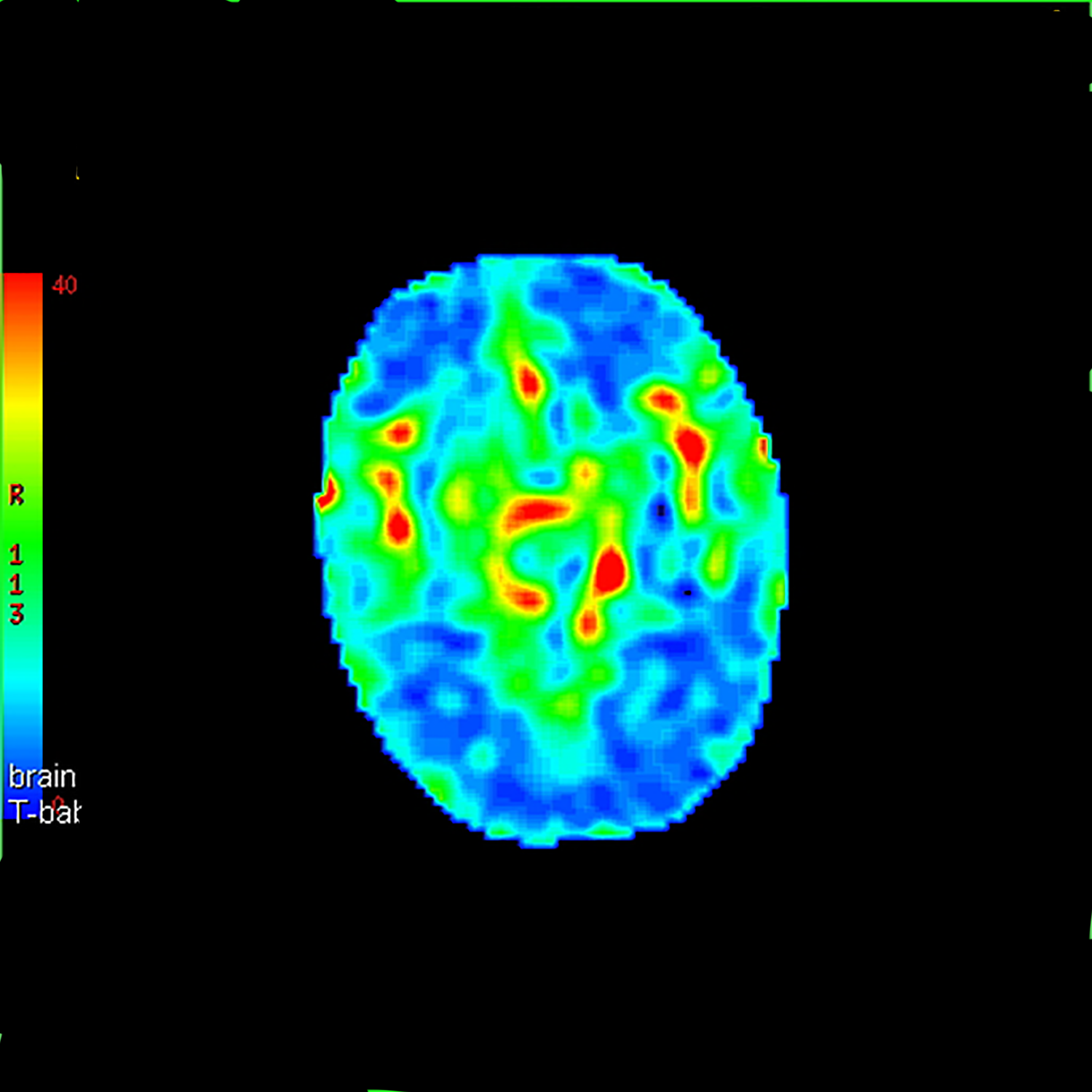

Supplement: S1 File — (ZIP) [file pone.0219284.s001.zip › patient CBF map/HIE34.tif]

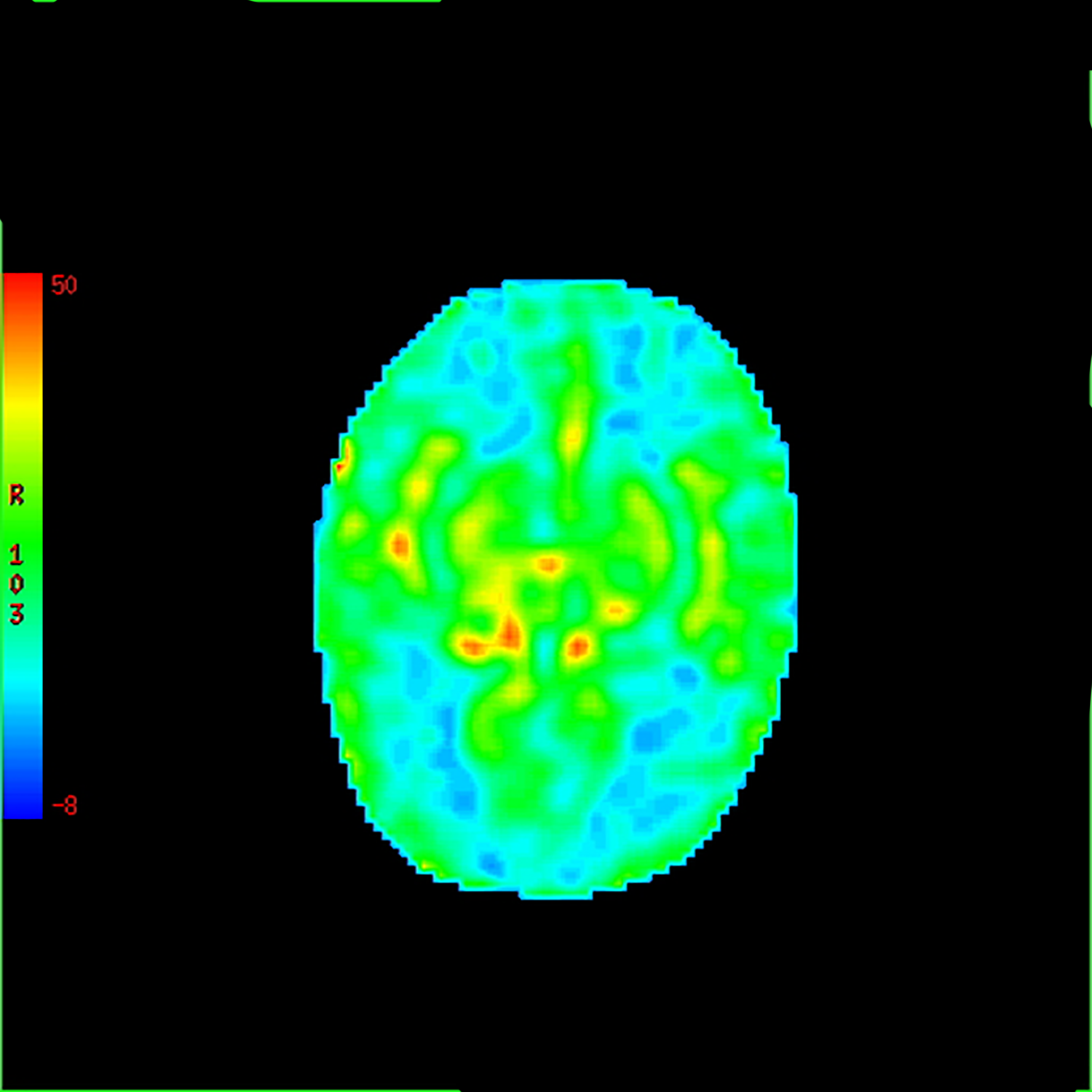

Supplement: S1 File — (ZIP) [file pone.0219284.s001.zip › patient CBF map/HIE35.tif]

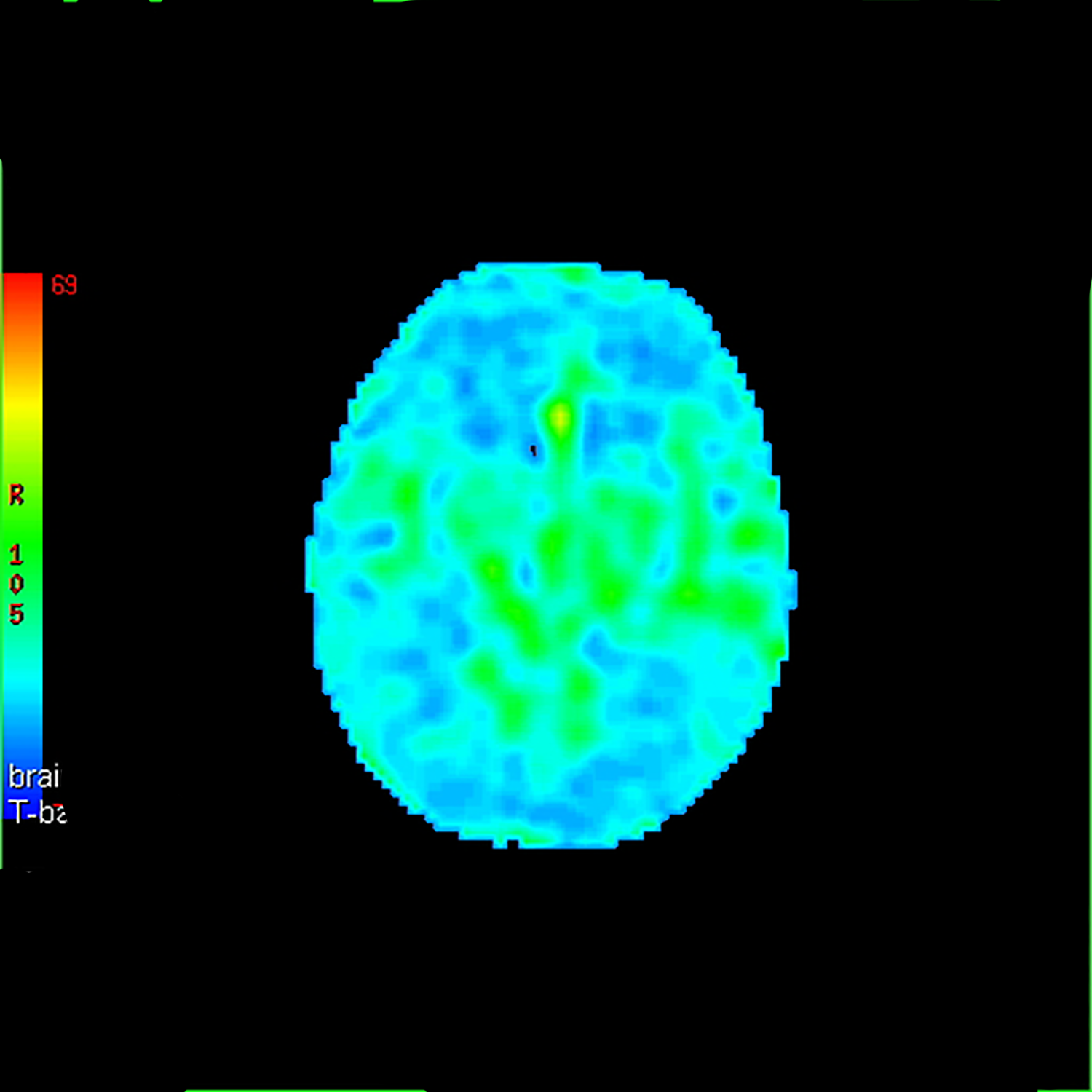

Supplement: S1 File — (ZIP) [file pone.0219284.s001.zip › patient CBF map/HIE36.tif]

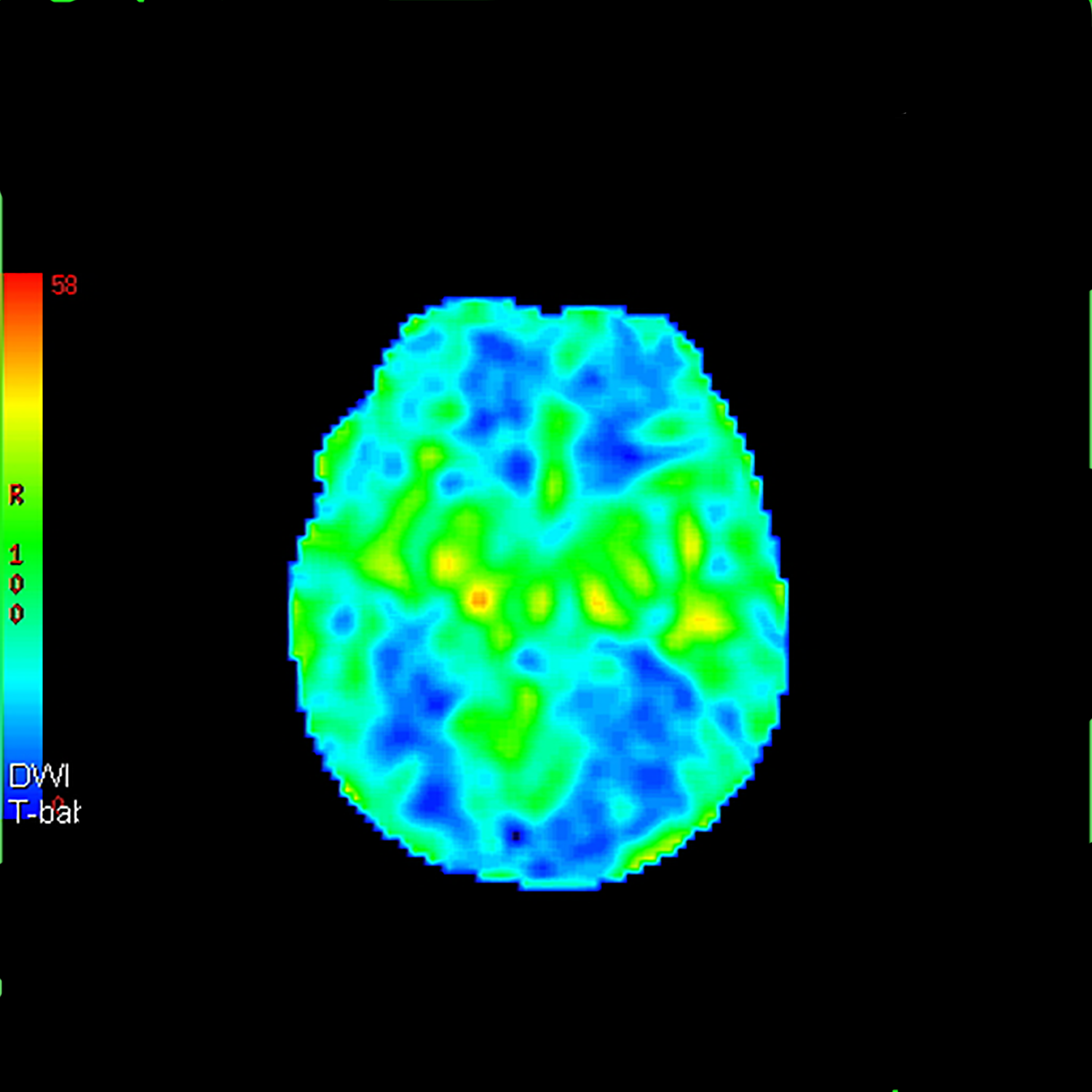

Supplement: S1 File — (ZIP) [file pone.0219284.s001.zip › patient CBF map/HIE37.tif]

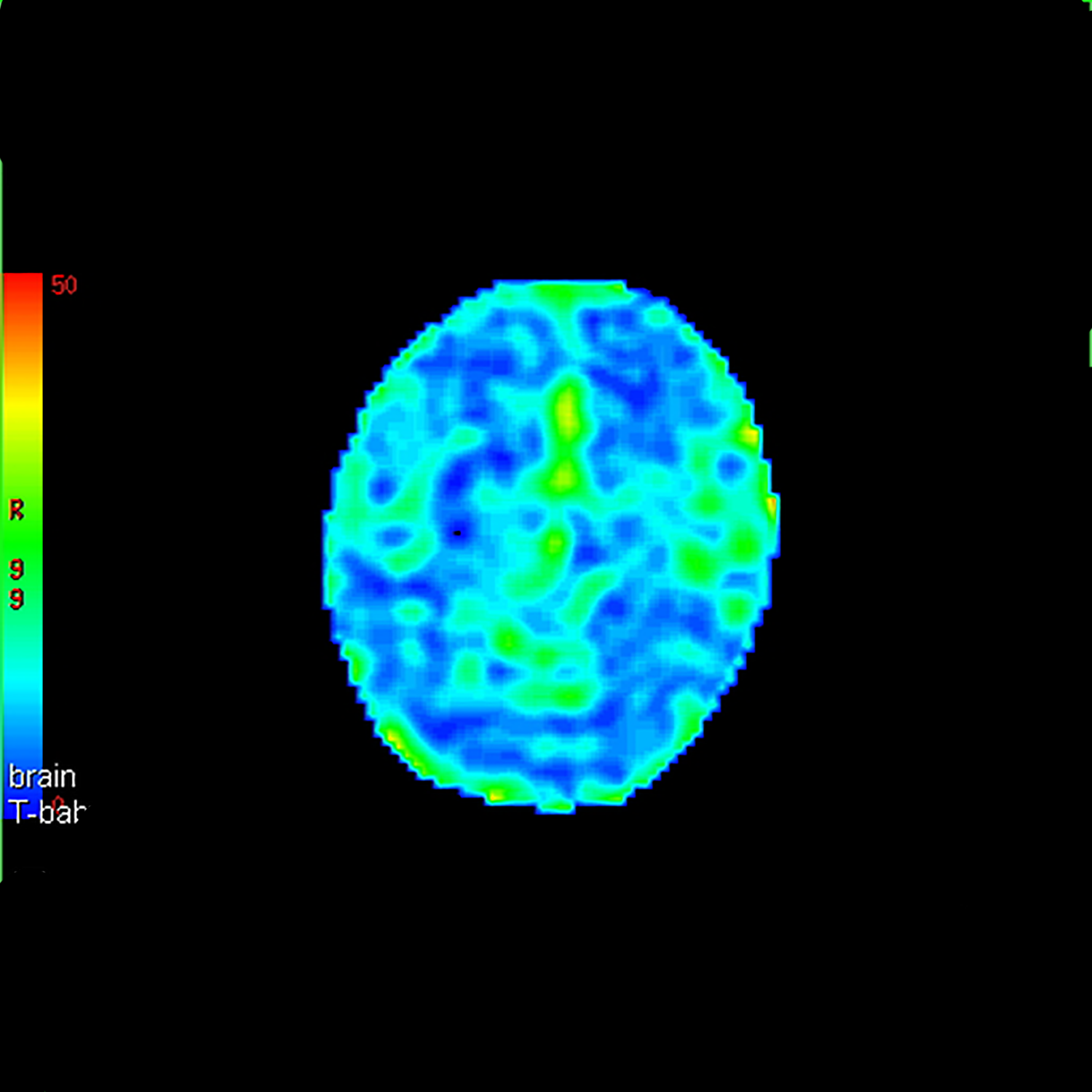

Supplement: S1 File — (ZIP) [file pone.0219284.s001.zip › patient CBF map/HIE38.tif]

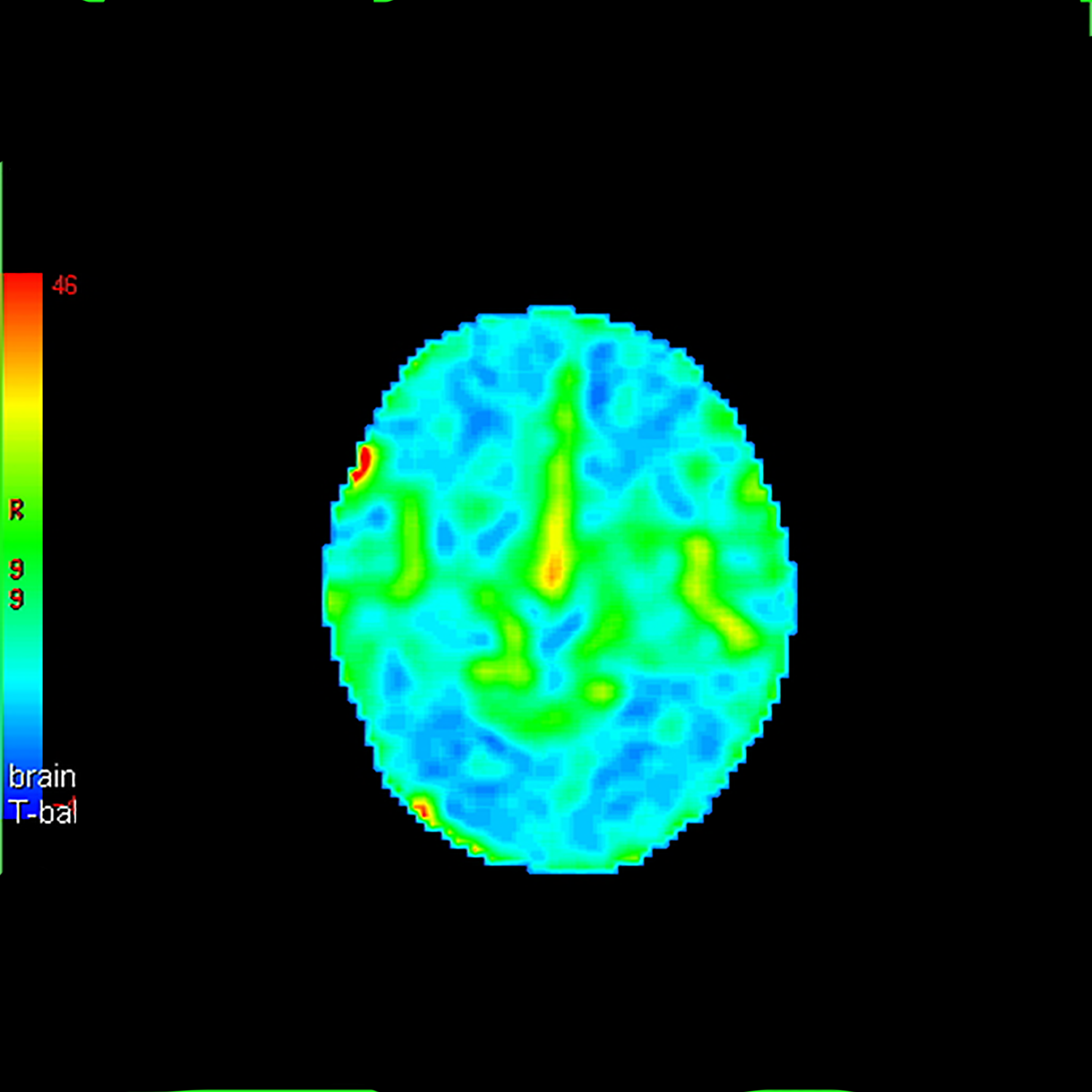

Supplement: S1 File — (ZIP) [file pone.0219284.s001.zip › patient CBF map/HIE39.tif]

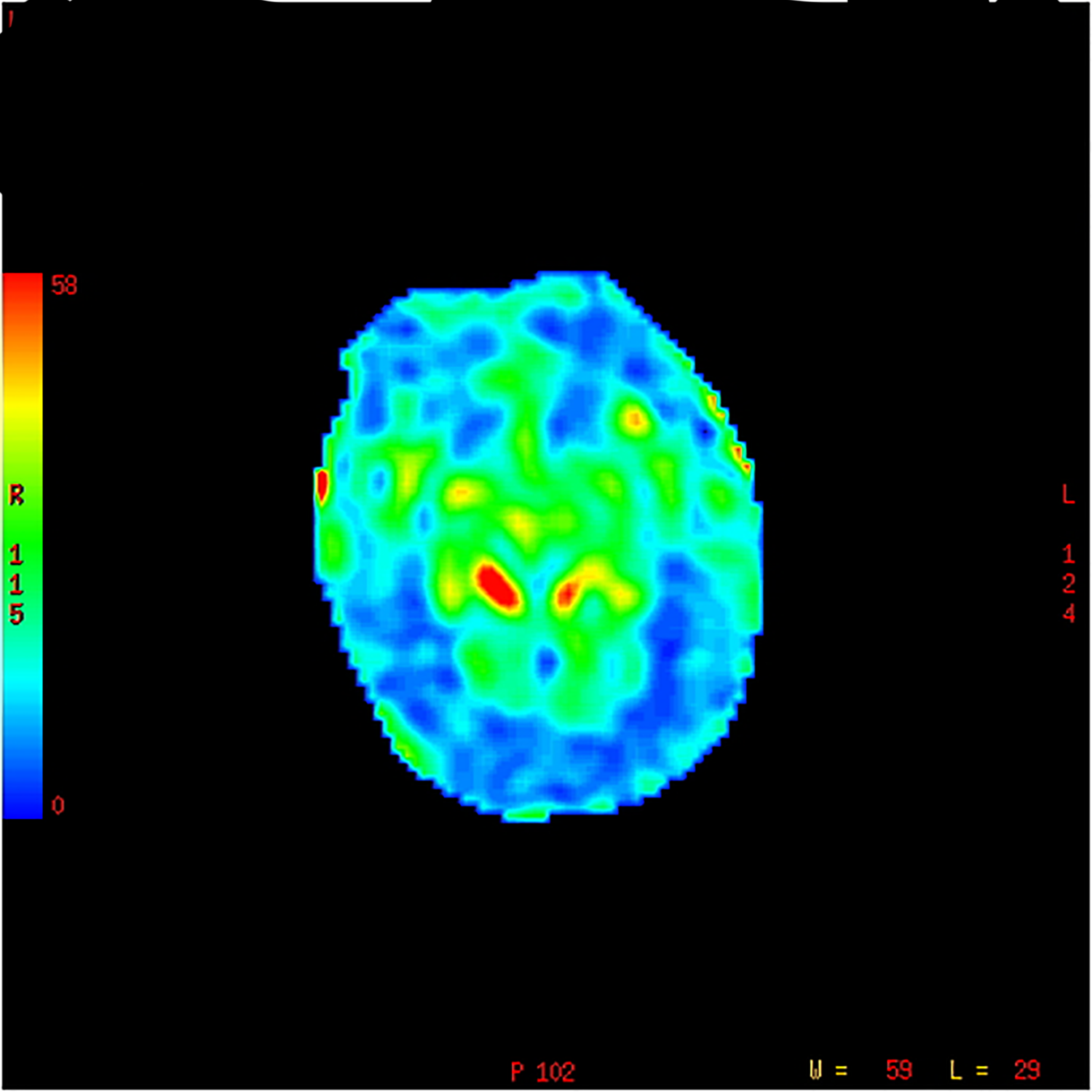

Supplement: S1 File — (ZIP) [file pone.0219284.s001.zip › patient CBF map/HIE4.tif]

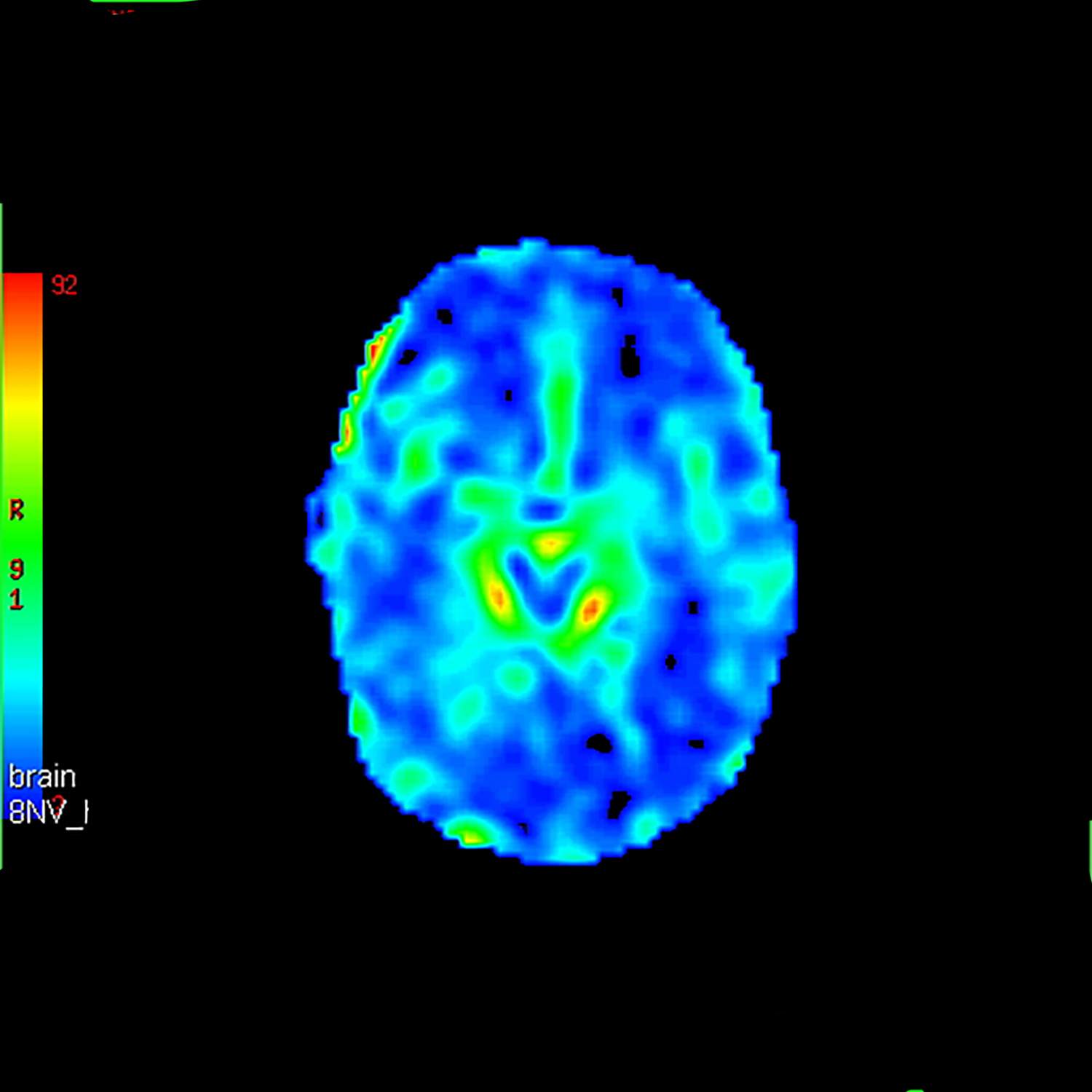

Supplement: S1 File — (ZIP) [file pone.0219284.s001.zip › patient CBF map/HIE40.tif]

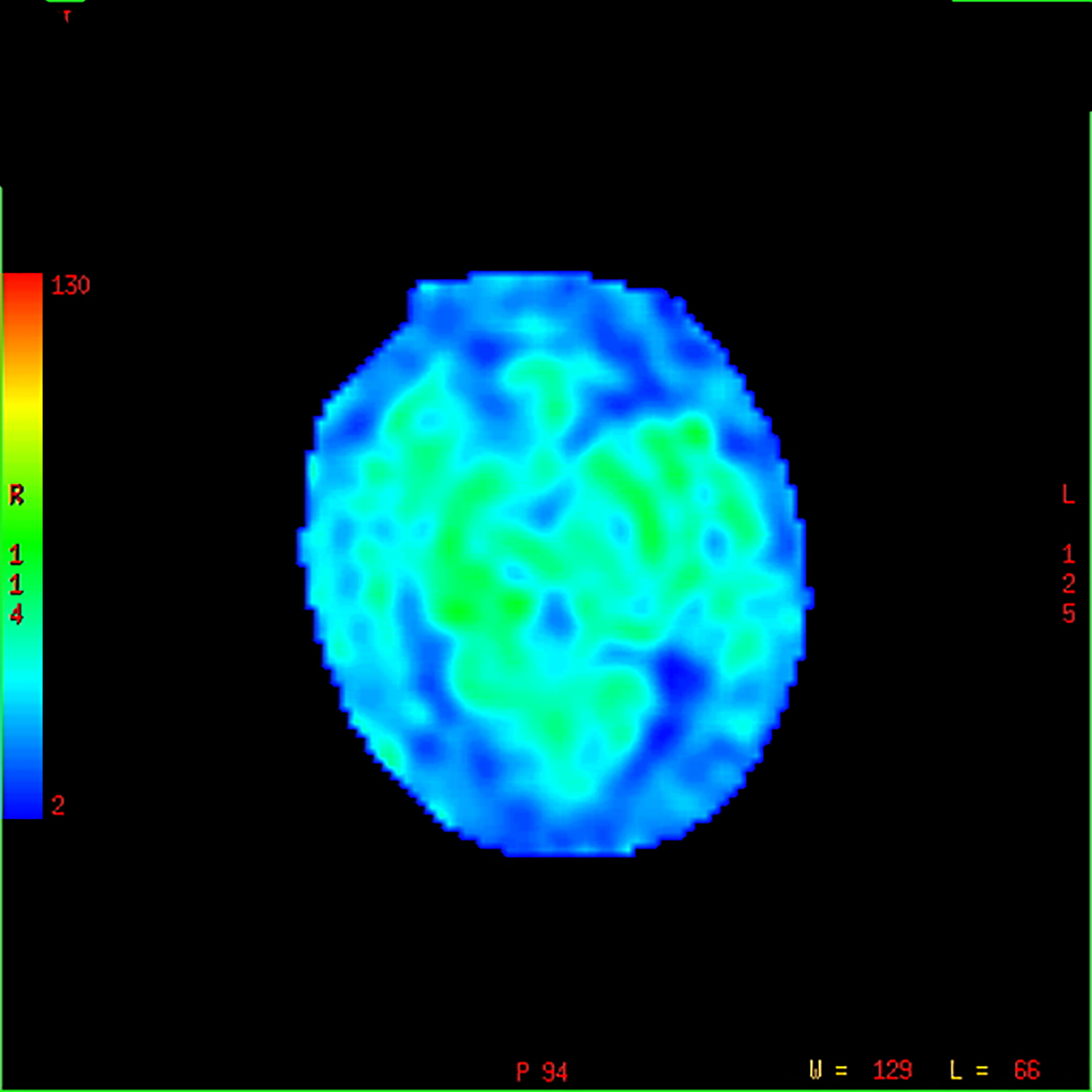

Supplement: S1 File — (ZIP) [file pone.0219284.s001.zip › patient CBF map/HIE5.tif]

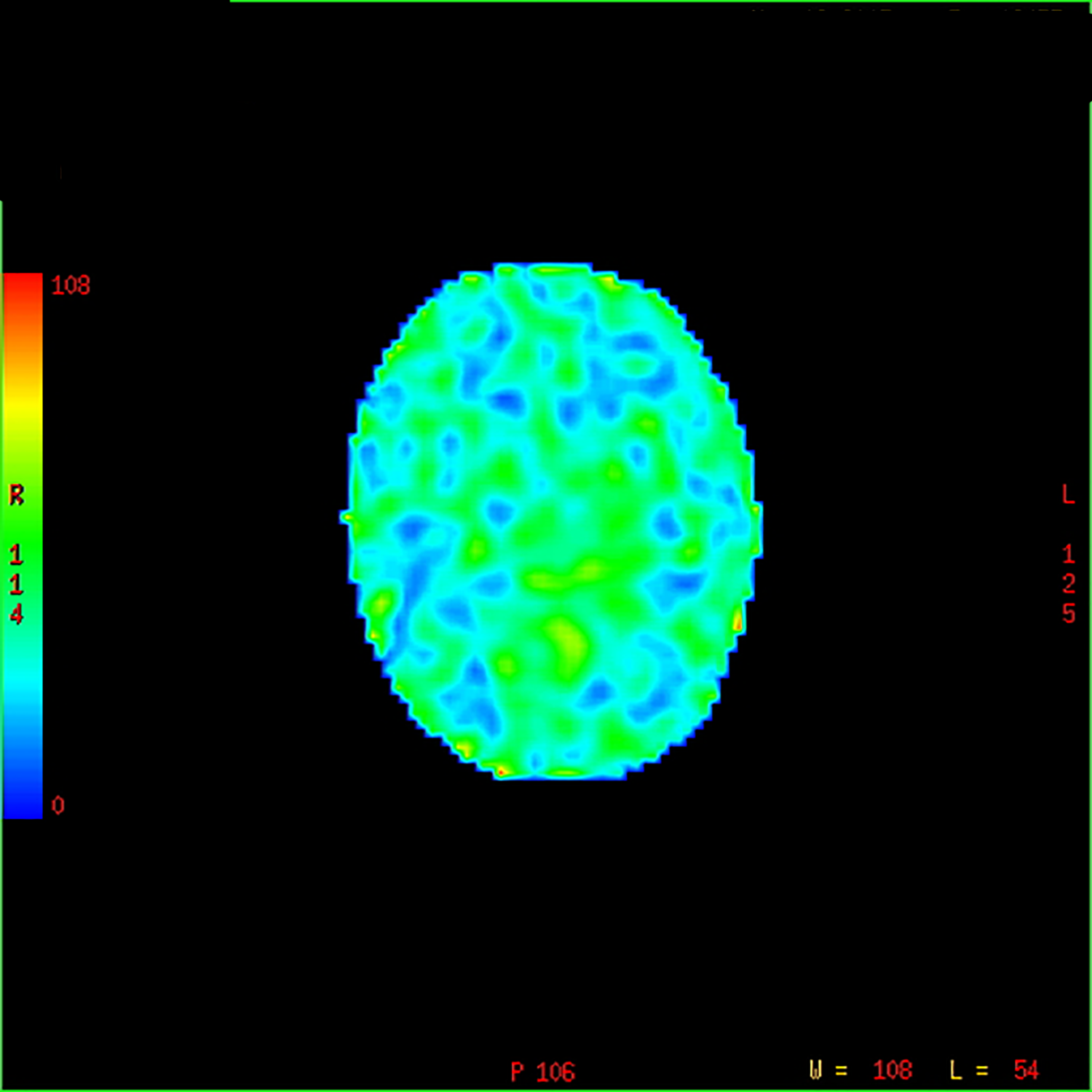

Supplement: S1 File — (ZIP) [file pone.0219284.s001.zip › patient CBF map/HIE6.tif]

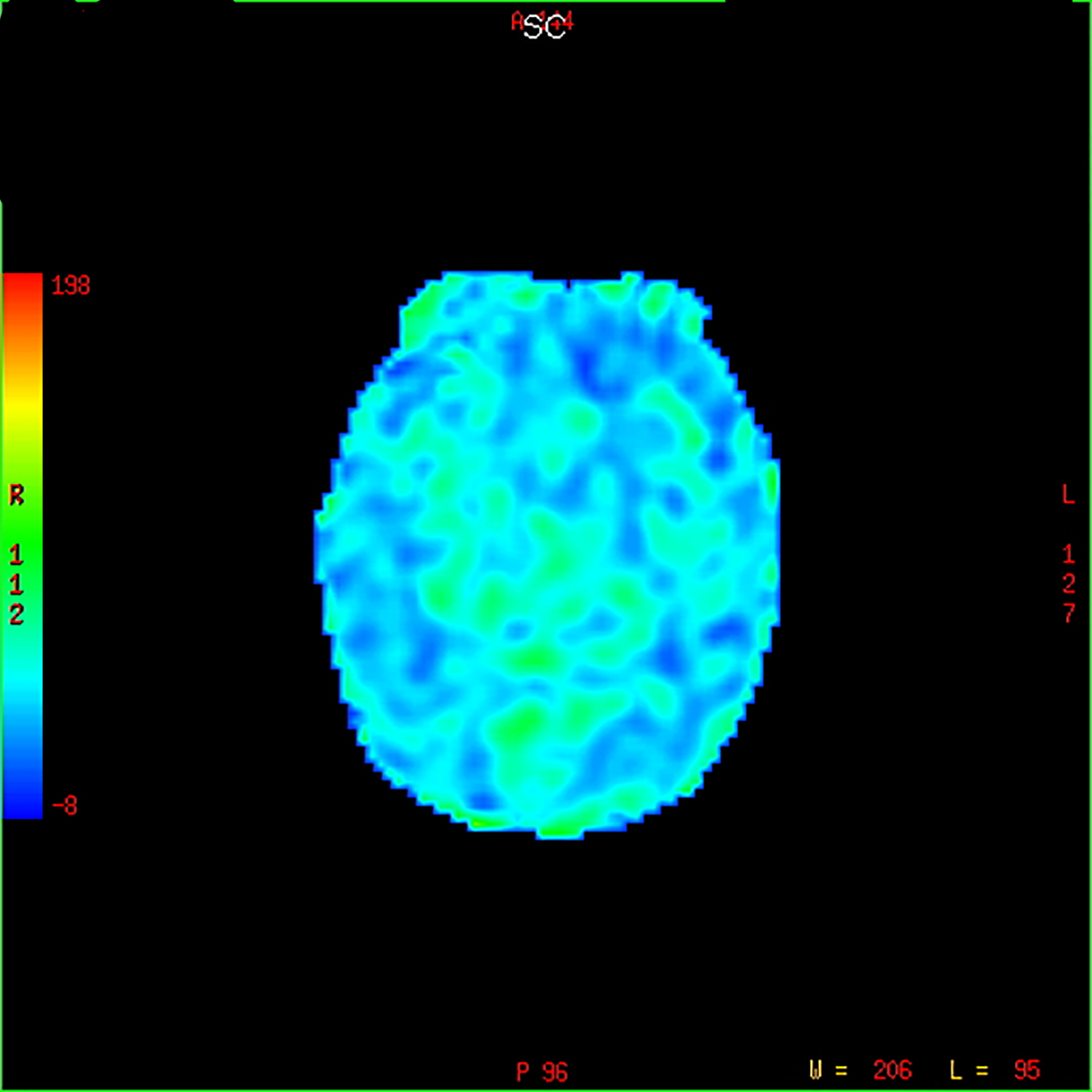

Supplement: S1 File — (ZIP) [file pone.0219284.s001.zip › patient CBF map/HIE7.tif]

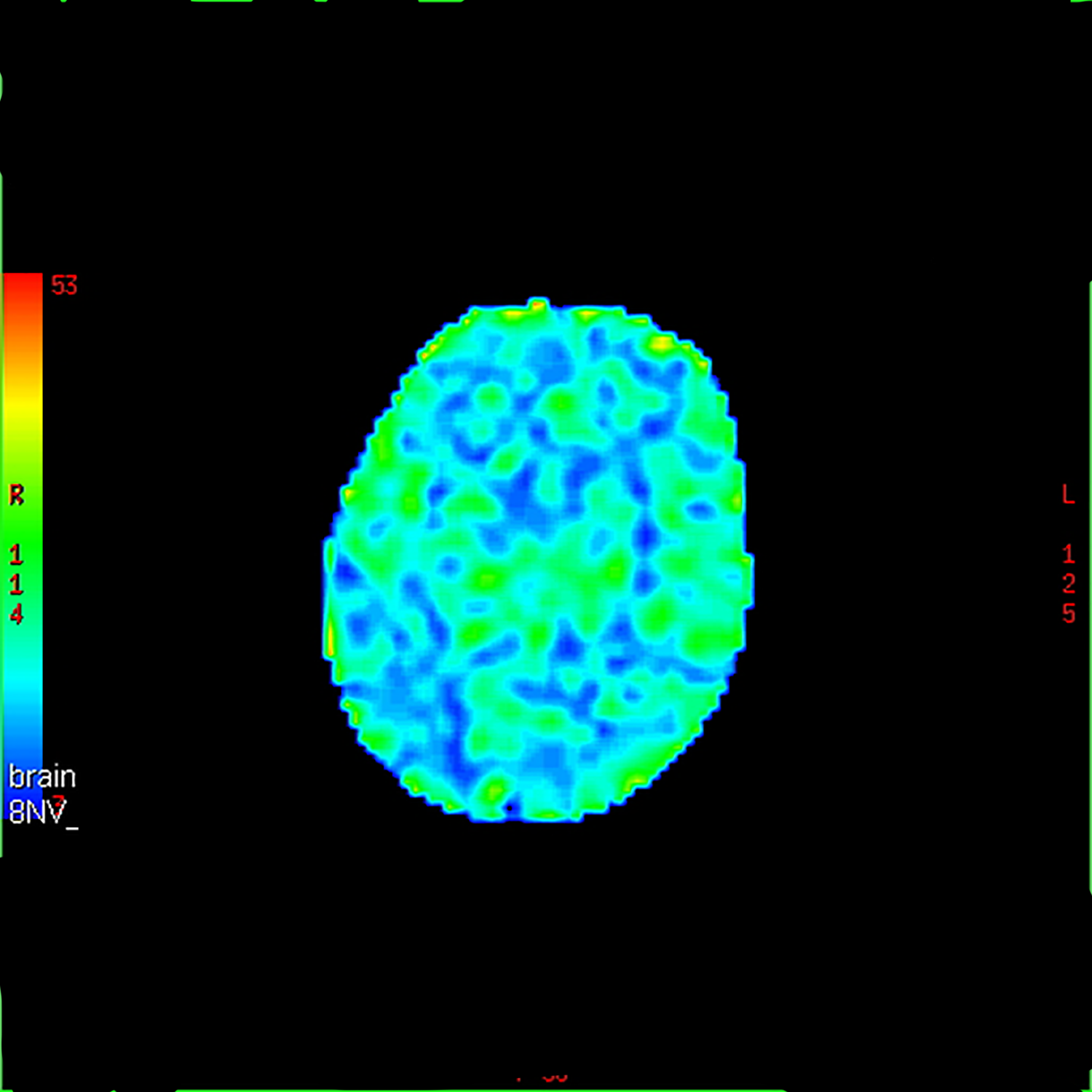

Supplement: S1 File — (ZIP) [file pone.0219284.s001.zip › patient CBF map/HIE8.tif]

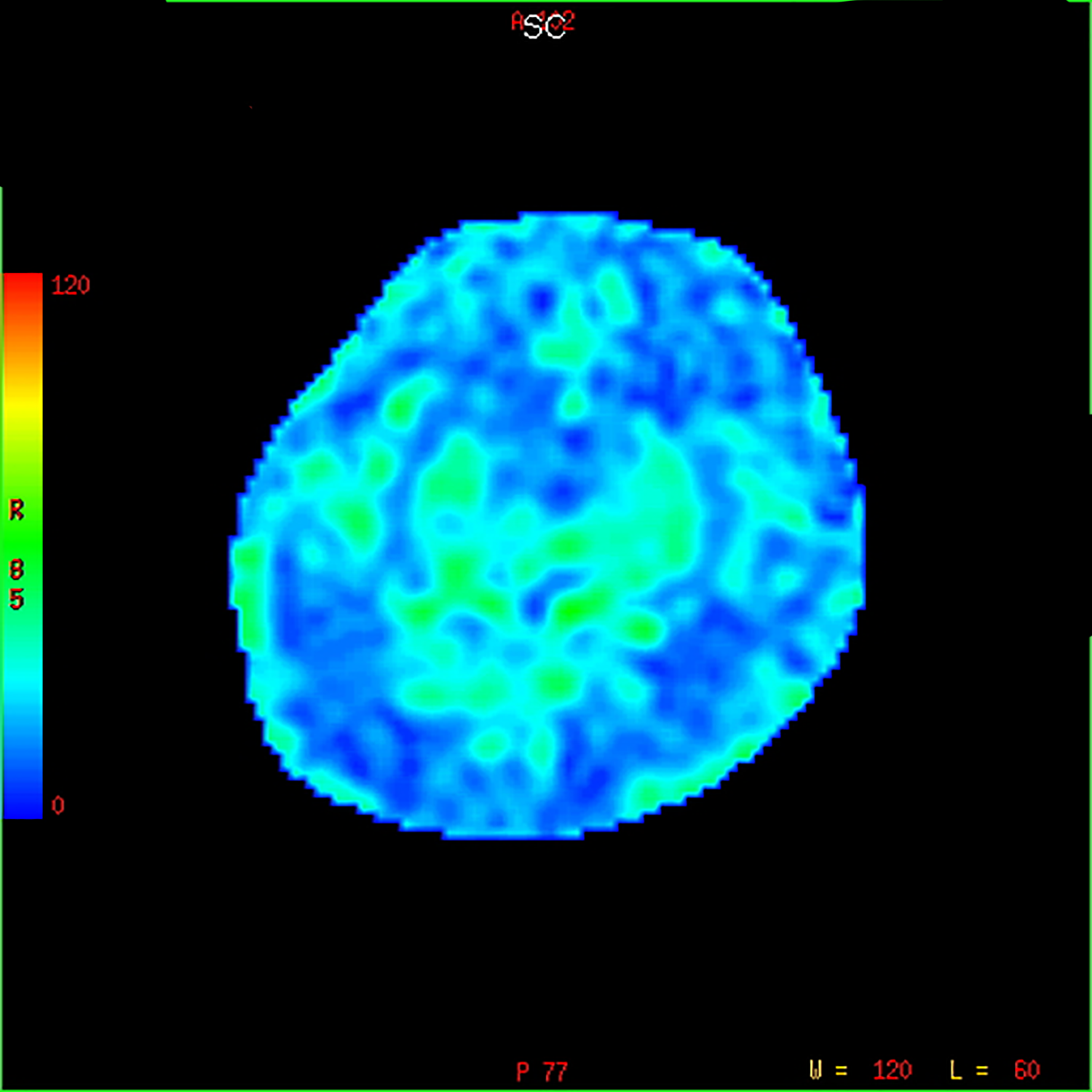

Supplement: S1 File — (ZIP) [file pone.0219284.s001.zip › patient CBF map/HIE9.tif]

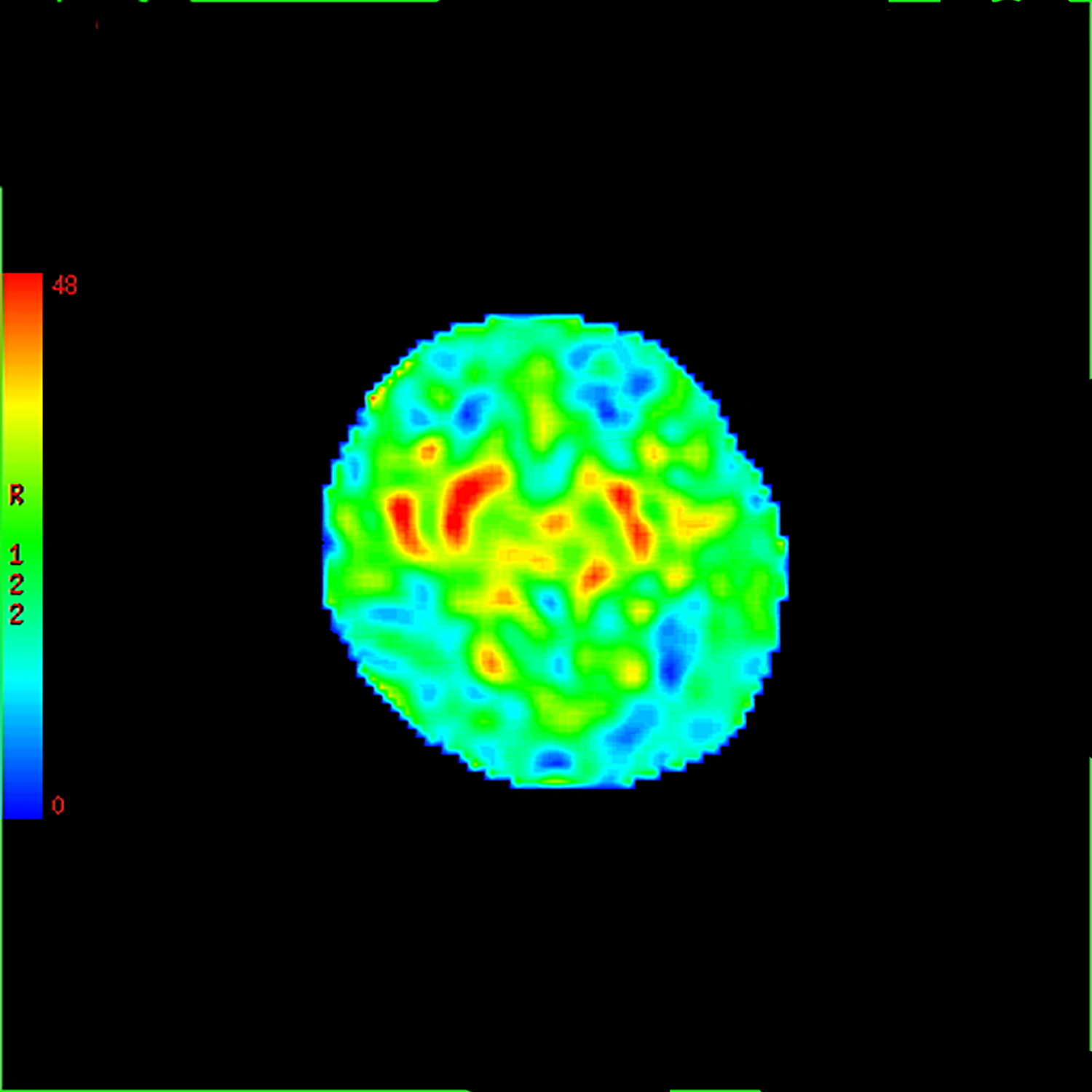

Supplement: S1 File — (ZIP) [file pone.0219284.s001.zip › patient CBF map/Healthy neonates1.tif]

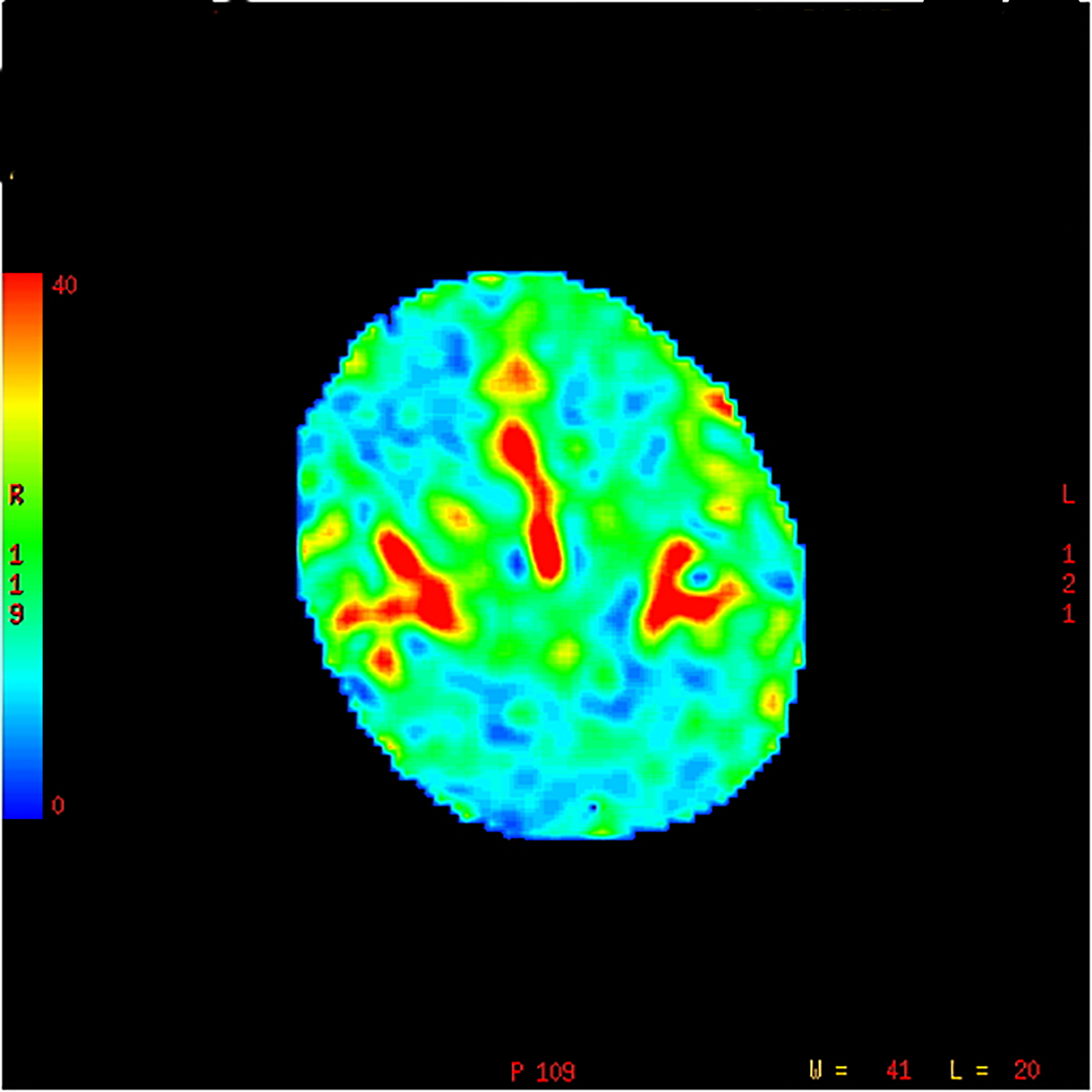

Supplement: S1 File — (ZIP) [file pone.0219284.s001.zip › patient CBF map/Healthy neonates2.tif]

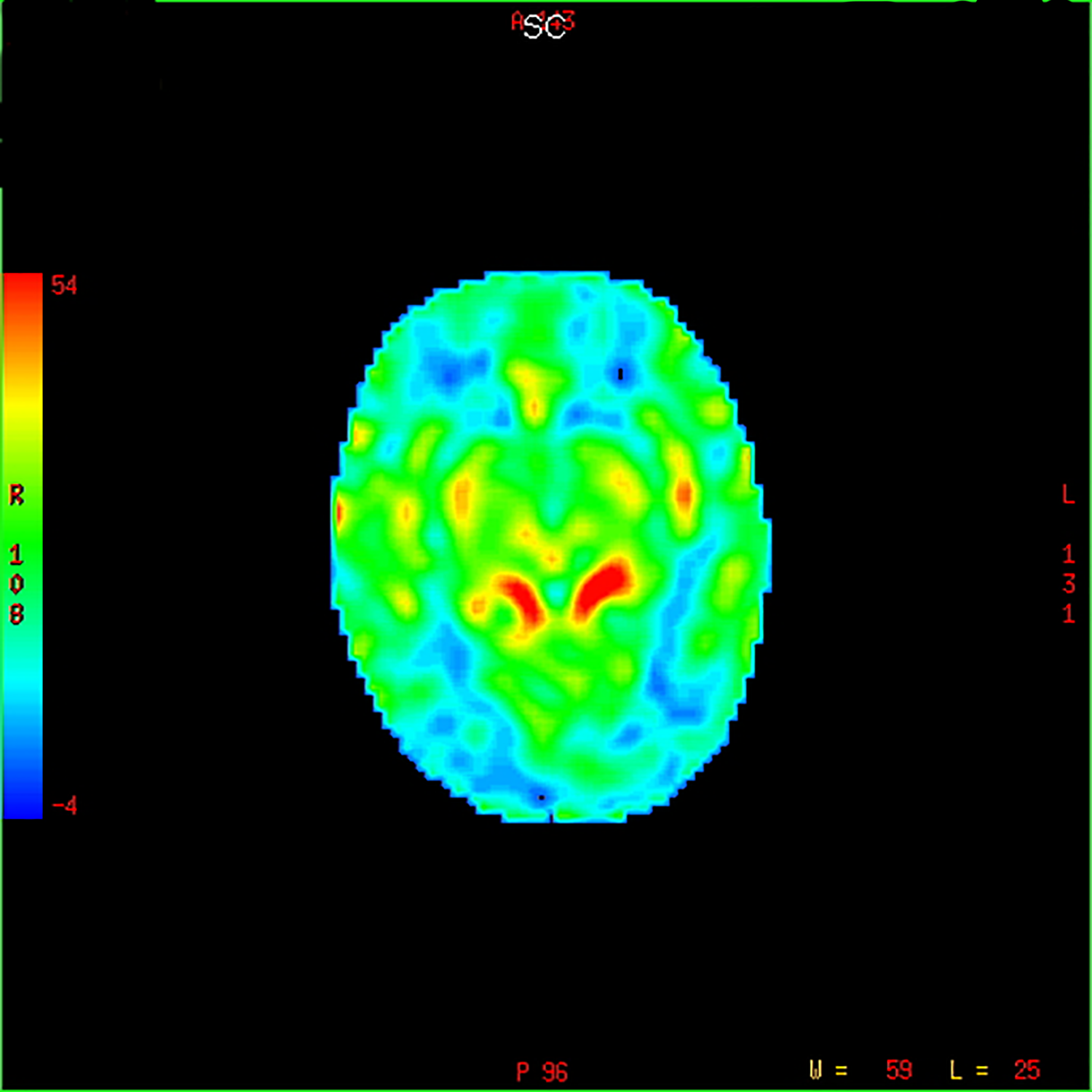

Supplement: S1 File — (ZIP) [file pone.0219284.s001.zip › patient CBF map/Healthy neonates3.tif]

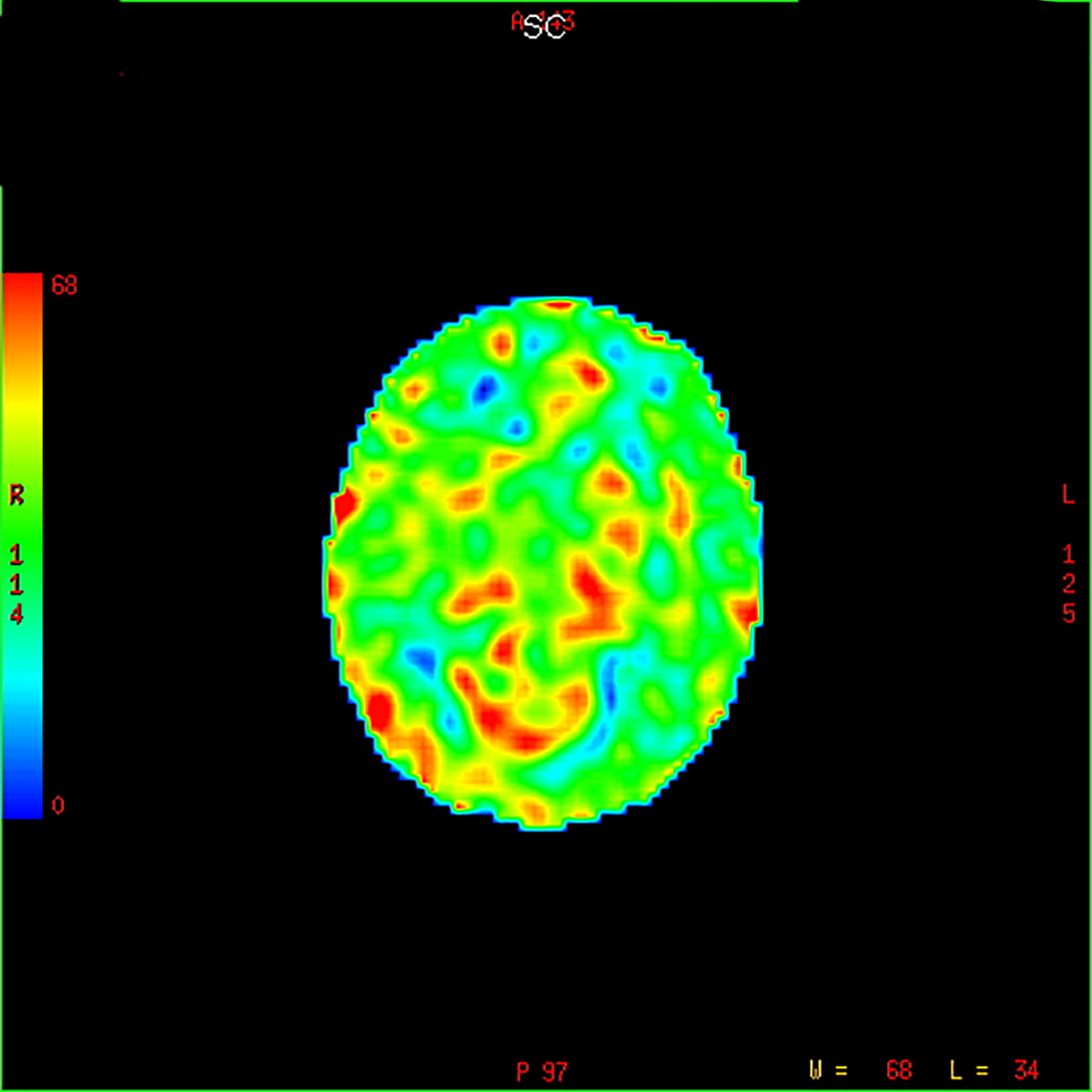

Supplement: S1 File — (ZIP) [file pone.0219284.s001.zip › patient CBF map/Healthy neonates4.tif]

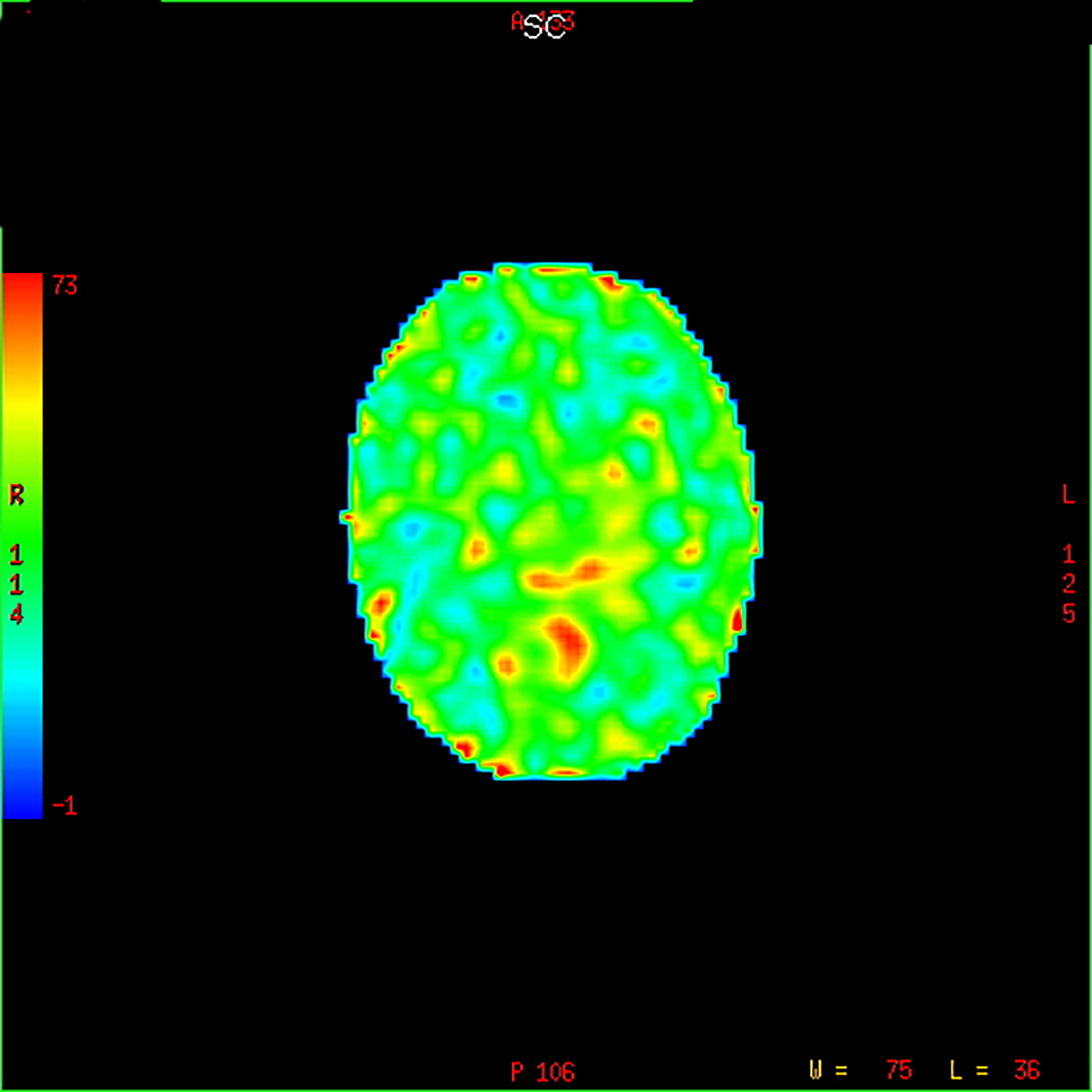

Supplement: S1 File — (ZIP) [file pone.0219284.s001.zip › patient CBF map/Healthy neonates5.tif]

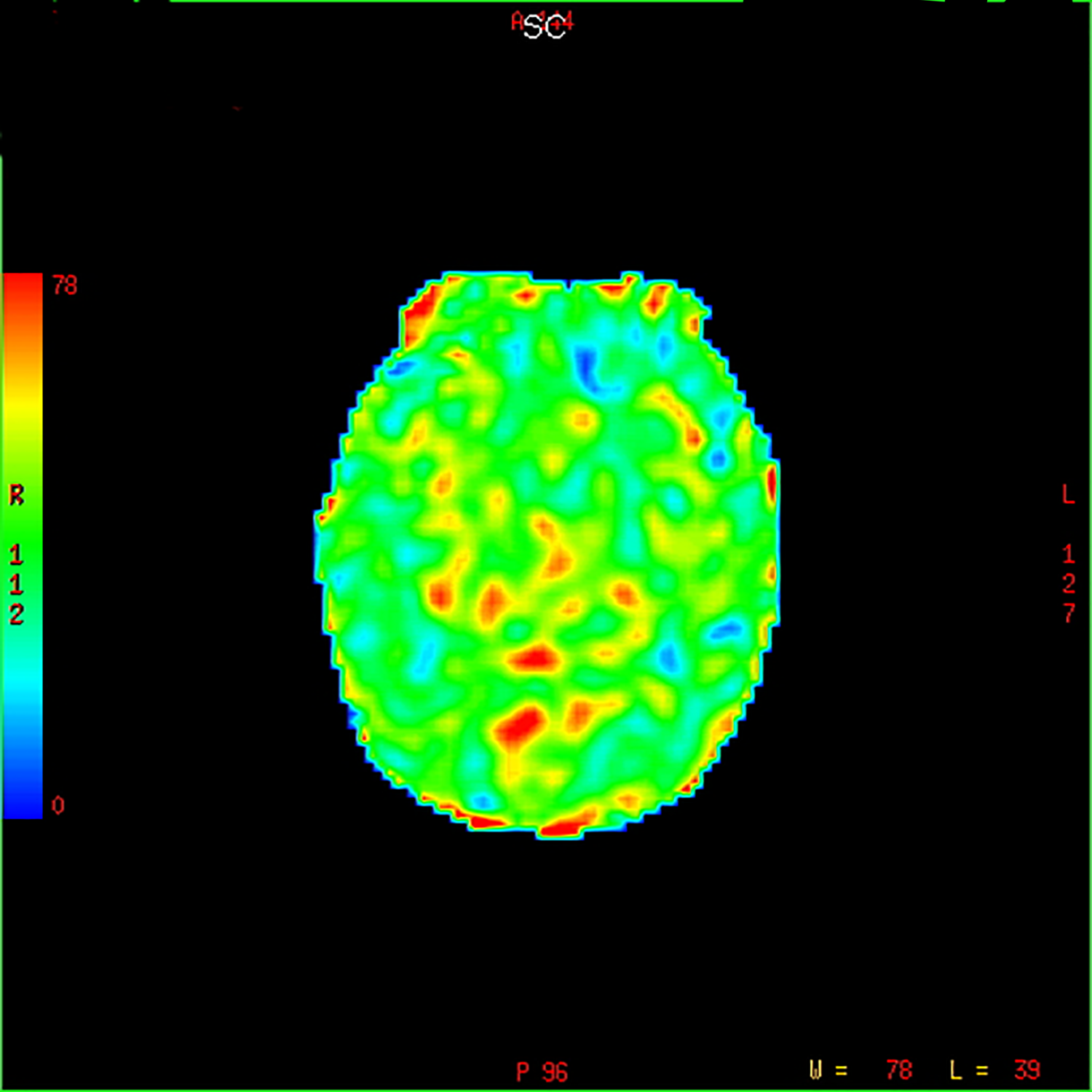

Supplement: S1 File — (ZIP) [file pone.0219284.s001.zip › patient CBF map/Healthy neonates6.tif]

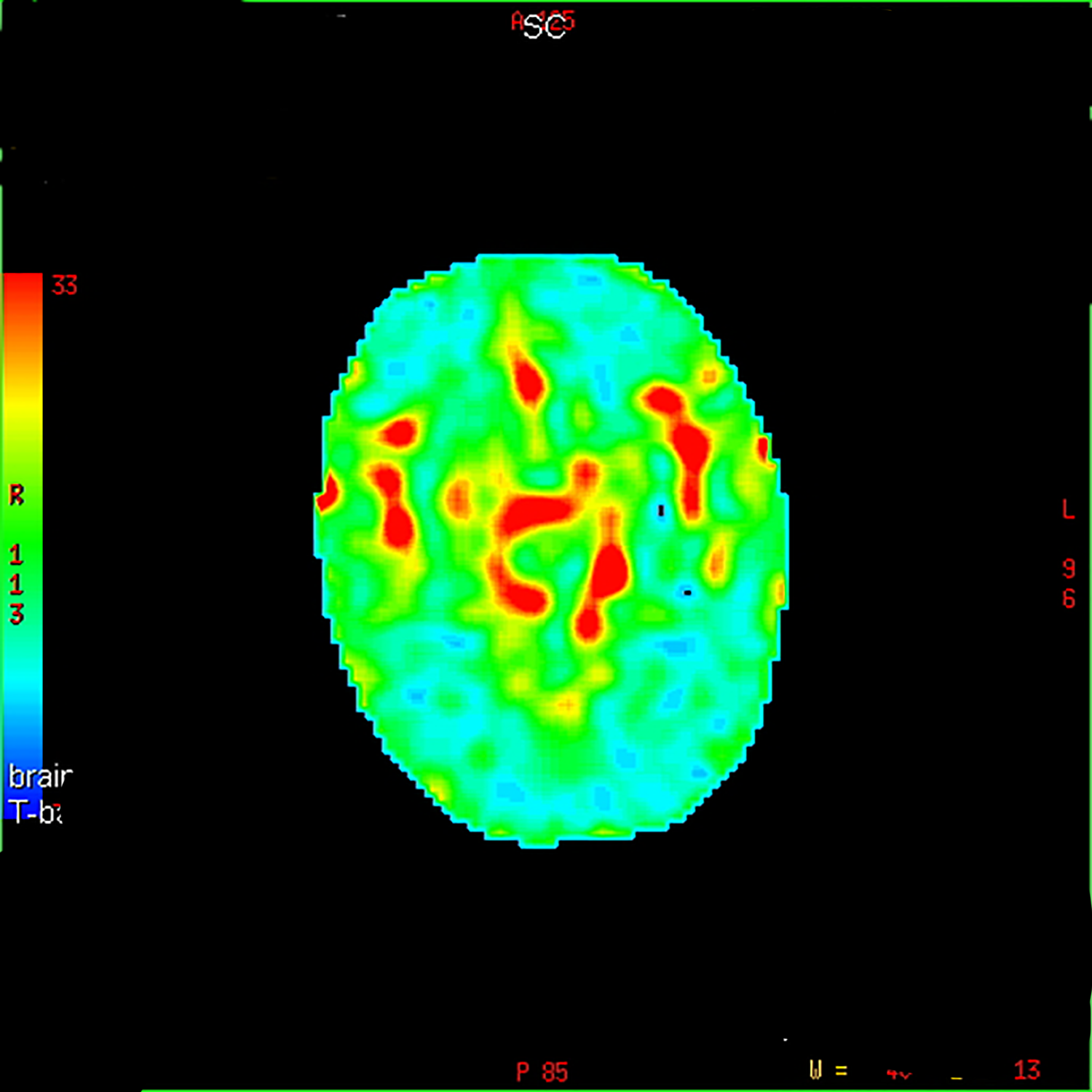

Supplement: S1 File — (ZIP) [file pone.0219284.s001.zip › patient CBF map/Healthy neonates7.tif]

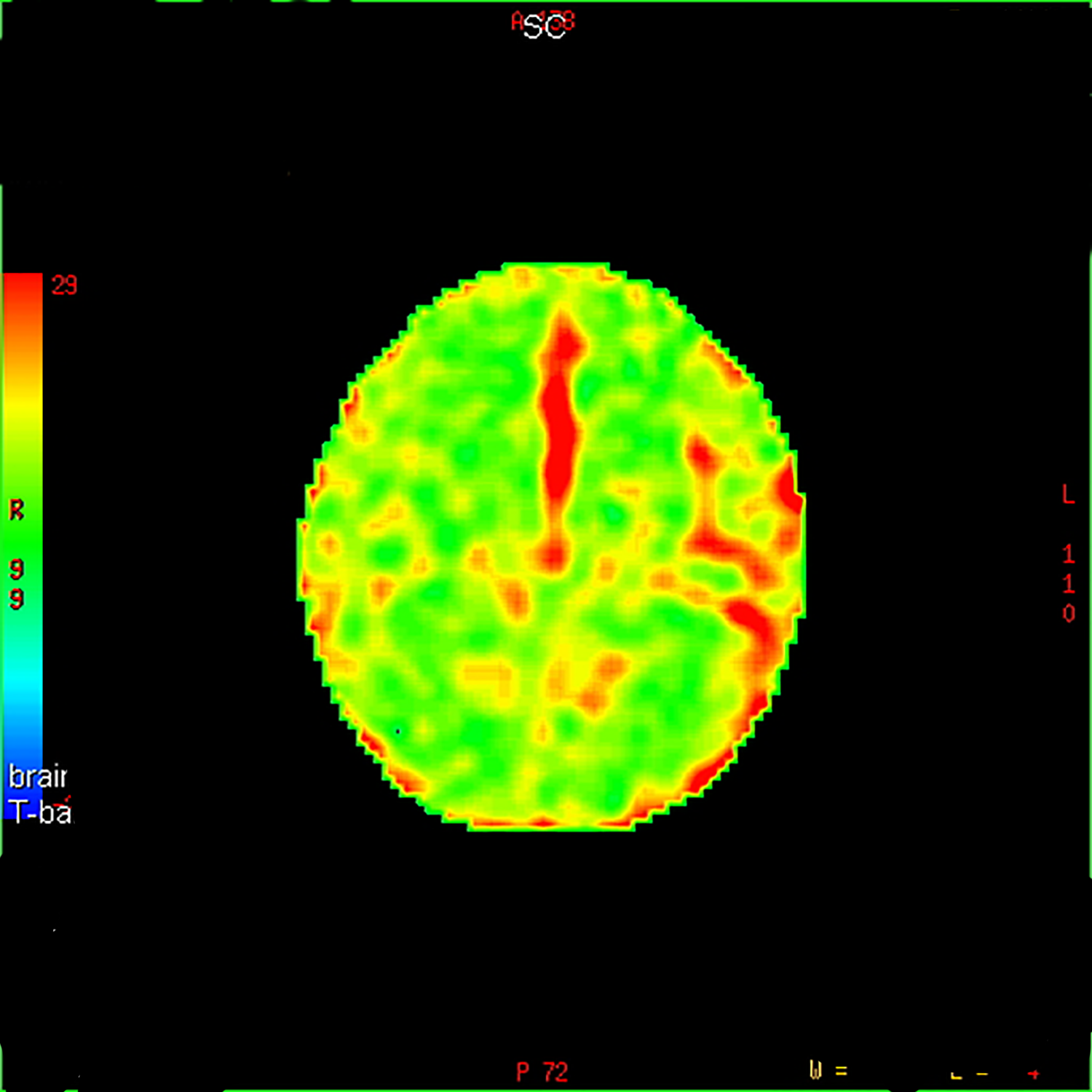

Supplement: S1 File — (ZIP) [file pone.0219284.s001.zip › patient CBF map/Healthy neonates8.tif]
